# Magnify is a universal molecular anchoring strategy for expansion microscopy

**DOI:** 10.1038/s41587-022-01546-1

**Published:** 2023-01-02

**Authors:** Aleksandra Klimas, Brendan R. Gallagher, Piyumi Wijesekara, Sinda Fekir, Emma F. DiBernardo, Zhangyu Cheng, Donna B. Stolz, Franca Cambi, Simon Watkins, Steven L. Brody, Amjad Horani, Alison L. Barth, Christopher I. Moore, Xi Ren, Yongxin Zhao

**Affiliations:** 1 Department of Biological Sciences, Carnegie Mellon University, Pittsburgh, PA, USA; 2 Department of Biomedical Engineering, Carnegie Mellon University, Pittsburgh, PA, USA; 3 Department of Neuroscience, Brown University, Providence, RI, USA; 4 Carney Institute for Brain Science, Brown University, Providence, RI, USA; 5 Department of Cell Biology, Center for Biologic Imaging, University of Pittsburgh, Pittsburgh, PA, USA; 6 Veterans Administration Pittsburgh, Pittsburgh, PA, USA; 7 Department of Neurology/PIND, University of Pittsburgh, Pittsburgh, PA, USA; 8 Department of Medicine, Washington University School of Medicine, St. Louis, MO, USA; 9 Department of Pediatrics, Washington University School of Medicine, St. Louis, MO, USA; 10 Department of Cell Biology and Physiology, Washington University School of Medicine, St. Louis, MO, USA

## Abstract

Expansion microscopy enables nanoimaging with conventional microscopes by physically and isotropically magnifying preserved biological specimens embedded in a crosslinked water-swellable hydrogel. Current expansion microscopy protocols require prior treatment with reactive anchoring chemicals to link specific labels and biomolecule classes to the gel. We describe a strategy called Magnify, which uses a mechanically sturdy gel that retains nucleic acids, proteins and lipids without the need for a separate anchoring step. Magnify expands biological specimens up to 11 times and facilitates imaging of cells and tissues with effectively around 25-nm resolution using a diffraction-limited objective lens of about 280 nm on conventional optical microscopes or with around 15 nm effective resolution if combined with super-resolution optical fluctuation imaging. We demonstrate Magnify on a broad range of biological specimens, providing insight into nanoscopic subcellular structures, including synaptic proteins from mouse brain, podocyte foot processes in formalin-fixed paraffin-embedded human kidney and defects in cilia and basal bodies in drug-treated human lung organoids.

## Editor’s Summary

Expansion microscopy with gel-anchored molecules enables simultaneous retention of nucleic acids, proteins and lipids in diverse tissue types.

A comprehensive understanding of a biological system requires precise knowledge of the spatial arrangement of components across length scales, from tissue-level organization to individual biomolecules. Expansion microscopy (ExM)[1, 2] is a technique enabling nanoscale imaging using only a diffraction-limited fluorescent microscope. ExM magnifies cells and tissues physically and isotropically: biomolecules are linked covalently to a dense and swellable polyelectrolyte hydrogel and are physically expanded away from each other in water after chemical processing. Since development of the technique[1], ExM protocols have been used for nanoscale imaging of proteins[3, 4, 5, 6, 7, 8, 9, 10], nucleic acids[7, 11, 12, 13] and lipids[8, 9, 14], while new hydrogel chemistries facilitate linear expansion factors (EFs) of around tenfold or larger in either a single[15, 16] or iterative[6, 10] expansion steps. To perform expansion, molecular handles are first covalently attached to specific biomolecules and/or labels that anchor them to a swellable hydrogel network that is subsequently synthesized throughout the specimen[2]. These handles are either conjugated antibodies or reactive chemicals prone to quick hydrolysis in water[2, 17, 18], with one exception that contains a mixture of formaldehyde and acrylamide for use with unfixed specimens[5]. Proteinase K (ProK) digestion, which destroys endogenous epitopes, is commonly used to enable expansion. Most protocols have demonstrated only around fourfold expansion in tissues[3, 4, 5, 6, 8, 9, 12, 13] with a mechanically delicate hydrogel, providing an effective resolution of only around 70 nm with a 1.15 numerical aperture (NA; roughly 280-nm diffraction limit) objective lens, which is insufficient to resolve fine features such as molecular organization within individual synapses. Protocols for larger expansion[6, 15, 16] have been developed, but have not been demonstrated beyond cultured cells and brain tissue sections.

**Fig. 1:**
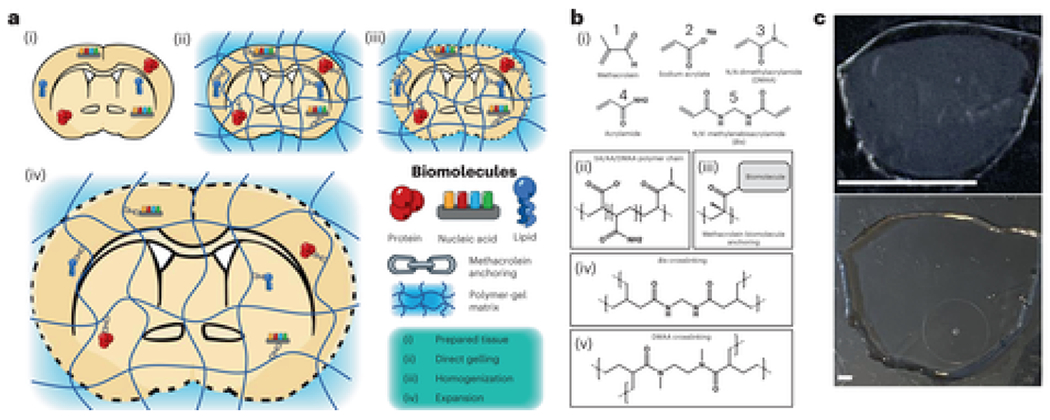

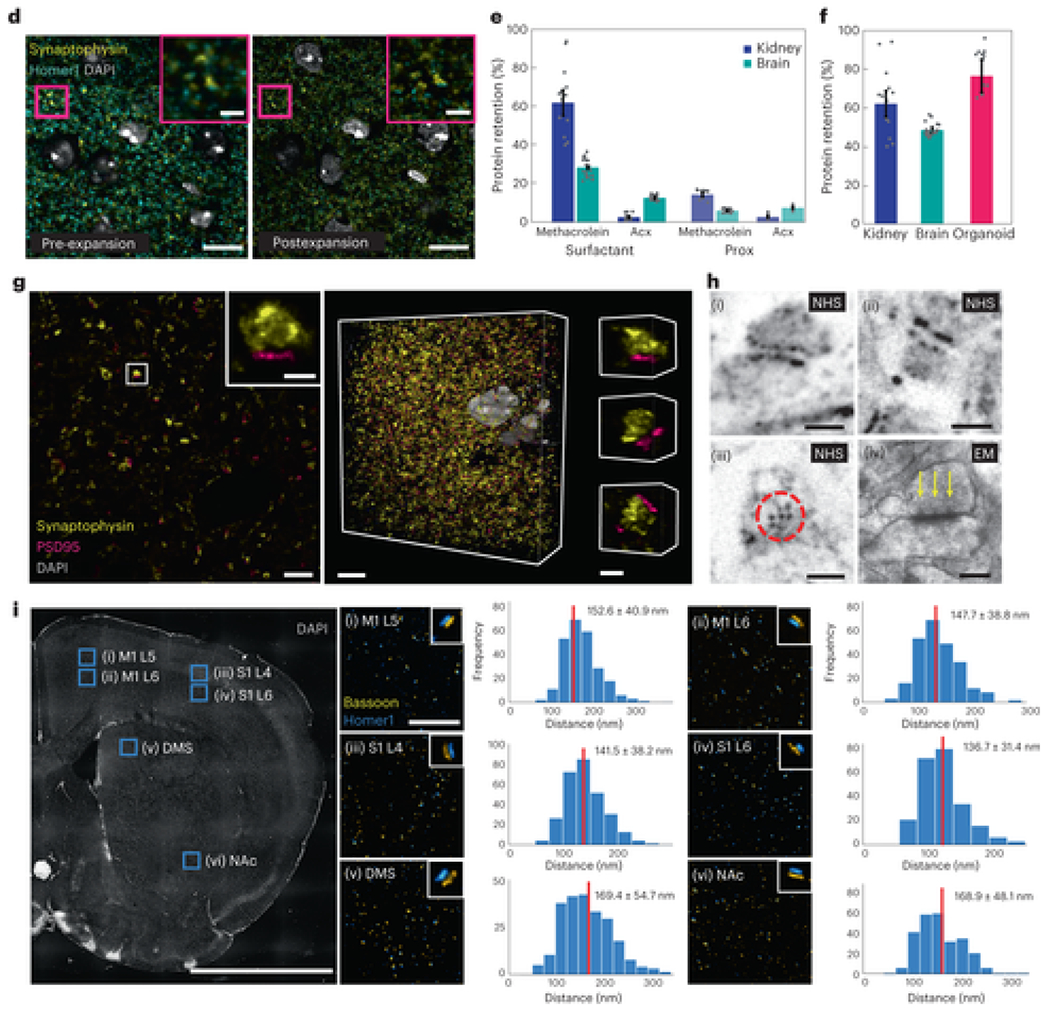
Design and validation of the Magnify protocol. **a**, Magnify protocol. **b**, Magnify gel chemistry. (i), Species participating in free radical polymerization. (ii)–(v), Example interactions of gel monomers, **c**, Magnify expanded mouse brain section. Top, after incubation and polymerization (as in **a**, (ii)). Bottom, after hot surfactant homogenization and full expansion in ddH_2_O (as in **a**, (iv)). The tissue has expanded uniformly and without distortion; a small piece (top right of the gel) was lost during liquid transfer. EF = 10.5×. **d**, Fluorescent signal is retained after proteolytic digestion. Insets, zoom ins of boxed regions. EF = 3.1× in PBS. **e**, Comparison of protein retention in FFPE human kidney sections (blue) and PFA-fixed mouse brain sections (green) for different anchoring and homogenization strategies. **f**, Comparison of protein retention across tissue types for the Magnify framework. **g**, Postexpansion immunostaining with Magnify. Left, synapses in the mouse striatum immunostained after Magnify processing with homogenization in surfactant solution. Inset, a single synapse in boxed region. Middle, 3D reconstruction of the same FOV shown in the left panel. Right, 3D reconstructions of individual synapses EF = ~11× in ddH_2_O. **h**, Magnify enables visualization of nanoscopic synaptic architecture. (i),(ii), Synapses in the mouse brain labeled for total protein content with a fluorescent NHS ester dye. EF = ~10× in ddH_2_O. (iii), A hexagonal lattice of dense projections in mouse brain tissue expanded with Magnify. EF = ~11× in ddH_2_O. (iv), Electron micrograph of a synapse from a separate mouse brain sample with visible dense projections (arrows). **i**, Measurement of homer-bassoon synaptic pair distances across the mouse brain with Magnify. Left, regions marked with blue squares. (i),(ii), Primary motor cortex layers 5 (M1 L5) and 6 (M1 L6). (iii),(iv), Primary somatosensory cortex layers 4 (S1 L4) and 6 (S1 L6). (v), DMS. (vi), NAc. EF = 3.6× in 1× PBS. Right, (i)–(vi), zoom ins of boxed regions; insets, representative synapses. Pair distance (center to center) was measured in each region. Scale bars, **c**, 5 mm; **d**, 10 μm; inset, 2 μm; **g**, left, 1 μm, left inset, 250 nm, middle, 5 μm, right, 250 nm; **h**, (i),(ii), 200 nm, (iii), 100 nm, (iv), 200 nm; **i**, tissue overview, 2 mm, zoom ins, 5 μm. Scale bars are all in biological scale.

An ideal ExM protocol would (1) be easy to use; (2) provide tenfold or greater expansion with minimal distortion; (3) be capable of conserving a comprehensive array of biomolecule classes that can be labeled after expansion and (4) be simultaneously compatible with a broad range of tissue types (including mechanically tough tissues such as kidney) and fixation methods. So far, no documented ExM method can achieve all these features. Here, we present Magnify, a variant of ExM that meets all the requirements above. Magnify uses a hydrogel formula that retains a spectrum of biomolecules, thus eliminating the need for a separate, molecule-specific anchoring step. Additionally, Magnify can expand conventionally preserved tissues up to about 11-fold, providing an effective resolution of around 25 nm for an around 280-nm diffraction-limited 1.15 NA objective lens (~280/11) (Fig. 1a–c and Supplementary Fig. 1). Magnify can also retain proteins, nucleic acids and lipids, enabling postexpansion labeling in a broad range of specimens. When combined with super-resolution optical fluctuation imaging (SOFI)[19], a computational postprocessing method employing independent temporal fluctuations of fluorophores to distinguish emitters, Magnify–SOFI can achieve a lateral effective resolution of around 15 nm with a 1.15 NA objective lens on a conventional spinning disk confocal microscope.

## Magnify: biomolecule-anchorable hydrogel with 11 times expansion

While ExM allows nanoscale imaging without expensive, specialized hardware, current iterations have critical limitations that minimize potential applications. For example, high (greater than tenfold) EF ExM methods have not been demonstrated successfully beyond cell culture or brain tissue, restricting many tissues to around 70 nm resolution. Although some ExM protocols can retain both nucleic acids and lipids directly within the gel, they rely on custom anchoring agents that are not available commercially, thus hindering widespread adoption. Therefore, there is a pressing need to develop a broadly applicable yet easily adoptable expansion method. To address this, we implemented Magnify by (1) eliminating the need for a standalone anchoring step; (2) formulating a mechanically robust hydrogel with a large EF and (3) developing a flexible homogenization strategy for an array of tissue types preserved with conventional fixation methods.

First, we asked whether it is possible to directly anchor biomolecules to the polymeric gel during gelation. Inspired by paraformaldehyde (PFA) fixation—known to preserve a wide range of biomolecules in tissue[20, 21]—we used methacrolein (**1**), a small molecule used in classic fixation protocols[20, 22], to incorporate biomolecules to the gel (Fig. 1a,b). Methacrolein remains stable in the monomer solution and modifies biomolecules in a similar way to formaldehyde while providing an isopropenyl functional group that participates in the in situ polymerization step. We found that tissue sections gelled with methacrolein-containing monomer solution can expand after strong protease digestion using ProK, while pre-expansion fluorescent markers (fluorescent proteins or immunolabels) persist (Fig. 1d, Supplementary Fig. 2 and Supplementary Note 1) as in previous ExM protocols[3]. We compared protein retention after protease digestion between tissues treated with our protocol and those treated with the protein retention ExM (proExM) protocol, which relies on Acryloyl-X, SE (AcX) to anchor biomolecules, and found over 380% higher retention in formalin-fixed paraffin-embedded (FFPE) kidney slices (Fig. 1e, Supplementary Fig. 2 and Supplementary Note 1). To preserve protein epitopes postexpansion, we treated gel-embedded tissues with a denaturant-rich solution at 80 °C for at least 1 h (optimized time varies by tissue type; Supplementary Table 1 and Supplementary Note 1) and observed crack-free expansion in mouse brain slices, a range of sectioned human tissues and human bronchial basal stem-cell-derived lung organoids with high protein retention using our protocol (Fig. 1e–f, Supplementary Fig. 2 and Supplementary Table 2). However, gel-embedded human kidney sections anchored with AcX showed distortions, including crack-like artifacts and incomplete expansion, after heat denaturation under the same conditions (Supplementary Fig. 2). We also found that the high protein retention facilitated by Magnify (530% higher retention in mouse brain slices as compared with proExM; Fig. 1e) enables effective delivery of antibodies postexpansion (Fig. 1g, Supplementary Fig. 3 and Supplementary Tables 4 and 5). This combined anchoring and gelling step along with homogenization with a hot denaturant-rich solution underlies the main concepts of the Magnify framework.

We sought to find an improved gel chemistry capable of providing a larger EF in a single round compared with the traditional fourfold of ExM (Supplementary Note 2). Previously, the X10 protocol[6] reported a hydrogel consisting of sodium acrylate (**2**) (SA) and N,N-dimethylacrylamide acid (**3**) (DMAA) that allows for EFs of around tenfold in cultured cells and mouse brain slices. However, X10 requires rigorous degassing to remove oxygen before gelling and reportedly cannot expand mechanically tough tissue types even with strong protease digestion[6]. We show that heavily formalin-fixed human tissues such as FFPE kidney sections did not expand evenly with the X10 gel and could only achieve around sixfold expansion (Supplementary Figs. 2 and 4) using heat denaturation. We then investigated a series of hydrogels by varying concentrations of the monomers DMAA, SA, acrylamide (**4**) (AA) and the crosslinker N,N’-methylenebisacrylamide (**5**) (Bis) (Supplementary Note 2 and Supplementary Tables 6–10). We discovered a hydrogel formula that is mechanically sturdy (Supplementary Fig. 4 and Supplementary Table 9), composed of 4% (w/v) DMAA, 34% (w/v) SA, 10% (w/v) AA and 0.01% (w/v) Bis (Supplementary Table 10), capable of expanding FFPE human kidney tissue sections by over 8.5-fold in water and freshly preserved mouse brain slices up to 11-fold after heat denaturation (Fig. 1c and Supplementary Table 6). Using a 1.4 NA ×60 oil immersion objective (around 200 nm Rayleigh limit), we demonstrated a resolving power of around 18 nm (200/11) in an 11-fold expanded mouse brain section stained for total protein content postexpansion with a fluorescently conjugated N-hydroxysuccidimidyl ester (NHS). Under these conditions, we observed well-resolved postsynaptic densities and presynaptic dense projections[23], which have been shown through electron microscopy (EM) to be around 56 nm in diameter and spaced roughly 20 nm apart edge-to-edge[24], as well as the hexagonal lattice these projections form when viewed on their face (Fig. 1h). The high EF of the Magnify gel allowed measurement of the distance between bassoon and homer—synaptic scaffolding proteins in the pre- and postsynaptic compartments, respectively—in mouse brain tissue expanded around fourfold in PBS using a 1.2 NA ×60 water immersion objective lens. We measured bassoon–homer pair distances in six brain regions (Fig. 1i): primary motor cortex layers 5 (M1 L5) and 6 (M1 L6), primary sensory cortex layers 4 (S1 L4) and 6 (S1 L6), dorsomedial striatum (DMS) and nucleus accumbens (nAc). While the measured pair distances for each region are in line with measurements taken with stochastic optical reconstruction microscopy[25], we noted distinct interregion variability, with cortical synapses having both a lower average pair distance and narrower distribution than synapses in either the DMS or nAc. Additionally, we measured synaptophysin and PSD95, synaptic proteins with a smaller edge-to-edge distance than homer and bassoon, in a fully expanded mouse brain slice using a 1.15 NA ×40 water immersion objective lens. Measuring from the edge of the synaptophysin signal (representing the presynaptic active zone) to the center of the postsynaptic density, we found a mean distance of 64.3 nm (Supplementary Fig. 3), consistent with previous reports[26].

**Fig. 2:**
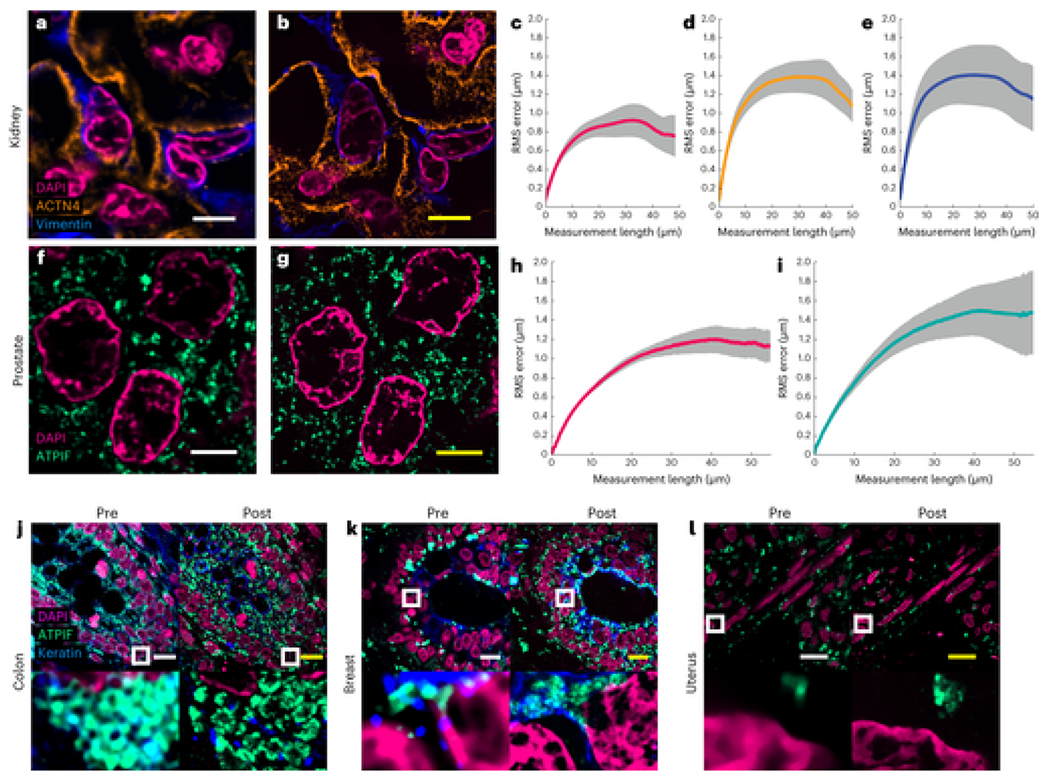

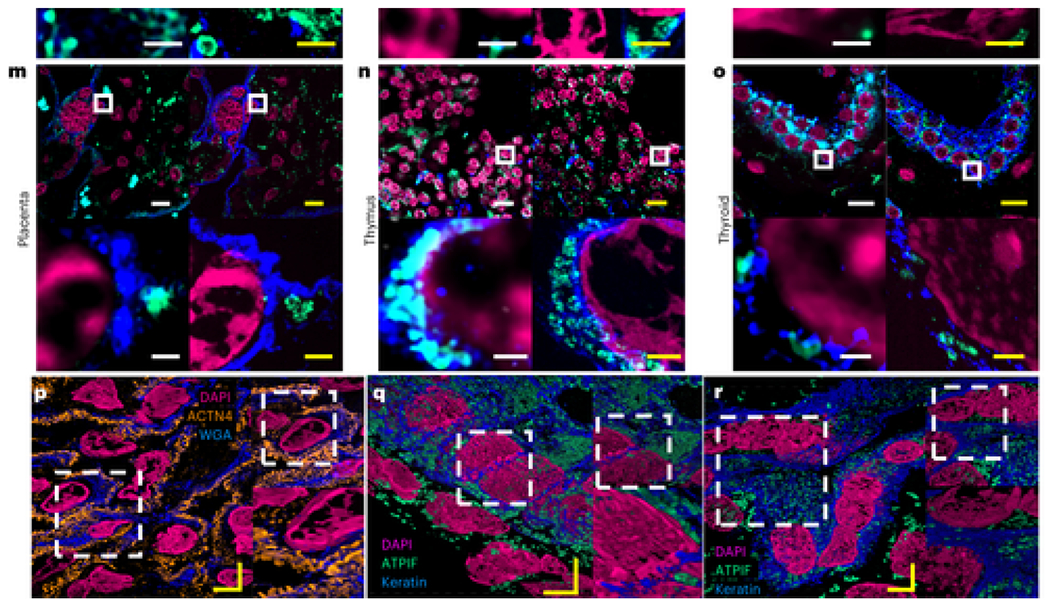
Validation of Magnify in several tissue types. **a,b**, Example of pre-expansion images of human kidney imaged at ×60 and processed with SOFI (**a**) compared with the same FOV postexpansion with Magnify taken at ×40 (**b**). Postexpansion images are maximum intensity projected over 25 frames in *z*. **c–e**, RMS length measurement error as a function of measurement length for pre-expansion versus postexpansion images for DAPI (**c**), ACTN4 (**d**) and Vimentin (**e**). Solid line, mean of channel; shaded area, s.e.m.; *n* = 5 technical replicates; average EF = 8.64× (s.e.m. 0.24). **f,g**, Example images of human prostate imaged as in **a** and **b**. Postexpansion images maximum intensity projected over three frames. **h,i**, RMS length measurement error as a function of measurement length for pre-expansion versus postexpansion images of DAPI (**h**) and ATPIF (**i**). Solid line, mean of channel; shaded area, s.e.m.; *n* = 4 technical replicates; average EF = 10.38× (s.e.m. 0.57). **j–o**, Validation of Magnify across several human tissue types. FFPE samples of human tissue were imaged at ×40 (top left). Images were taken at ×60 and processed with SOFI (bottom left). The white box indicates the FOV of the higher magnification images. The samples were then processed with the Magnify protocol, and the same FOVs were imaged postexpansion in water at ×10 (top right) and ×40 (bottom right). Postexpansion images were projected over 4–17 *z* slices. EFs in water were colon 8.85× (**j**), breast 9× (**k**), uterus 8× (**l**), placenta 8.75× (**m**), thymus 10.00× (**n**) and thyroid 10.59× (**o**). **p–r**, Example 3D images of human tissues: kidney (EF = 8.68×) (**p**), colon (EF = 9.67×) (**q**) and uterus (EF = 8×) (**r**). Dashed white boxes, zoomed in regions. Scale bars (yellow, postexpansion images). **a**, 5 μm; **b**, 5 μm (physical scale postexpansion, 40.75 μm; EF = 8.15×); **f**, 5 μm; **g**, 5 μm (physical scale postexpansion: 51.9 μm; EF = 10.38×); **j–o**, top, 10 μm; bottom, 1 μm; **p–r**, 5 μm. Scale bars are all in biological scale.

We confirmed the low distortion obtained by the Magnify protocol on several tissue types using SOFI pre-expansion[19] (Supplementary Fig. 5) and confocal microscopy postexpansion (Fig. 2 and Supplementary Fig. 4). We found no substantial morphological changes between pre-expansion images and postexpansion images of cell nuclei and protein markers in either macroscopic or subdiffraction levels (Fig. 2a–i and Supplementary Figs. 4, 6 and 7). The distortion levels were calculated in a range of 2–3.5% over length scales of tens to hundreds of microns in FFPE samples (Fig. 2c–e,h–i ) and 2.5% for human embryonic kidney 293FT cells (HEK-293FT) cells (Supplementary Fig. 4 ), consistent with previous reports[5, 6]. Analysis of the ultrastructure of microtubules in U2OS cells and cilia in human lung organoids (Supplementary Fig. 6 and Supplementary Note 6 ) showed average peak-to-peak distances of 22.68 nm ± 0.71 nm and 24.72 nm ± 0.72 nm (mean ± s.e.m.) respectively. The results agree with EM images of cilia in human lung organoids, which had an average peak-to-peak distance of 22.09 nm ± 0.34 nm (mean ± s.e.m.). The linkage errors determined by the affinity agent are smaller than 5 nm in both cell culture and organoids, most probably due to the smaller relative size of the antibody labels compared with the width of microtubule postexpansion[27]. We also found that the slightly higher distortion level with some labels compared with previously reported ExM protocols is due to the increased number of labeled features during postexpansion staining with Magnify (Supplementary Fig. 3).

FFPE specimens are among the most important biopsy preparations used for clinical examination, pathology research and diagnostic/drug development. Due to the heavy formaldehyde-induced peptidyl crosslinks, FFPE specimens are challenging to expand with preserved epitopes since current protocols require aggressive proteolytic digestion[7, 28]. We tested Magnify on several FFPE tissues and tissue microarrays containing tissue sections from various human organs and corresponding tumors, including breast, brain and colon (Fig. 2j–o and Supplementary Fig. 7 ). In all cases, we obtained EFs of around 3.5–4.5× in PBS, with an average EF ranging from 8.00× to 10.77× in water depending on tissue type with 3.9% intraclass variation (Supplementary Table 1). We observed that methacrolein concentration and homogenization time need to be adjusted for different tissue types and preparations (Supplementary Table 1). FFPE tissue sections required longer homogenization times and higher methacrolein concentrations to achieve full expansion than those with freshly preserved specimens due to higher crosslinker content caused by formalin fixation. The difference in tissue preparations, methacrolein concentration and homogenization time may contribute to the 10% interclass variation in EFs. In FFPE specimens, we observed that postexpansion staining with Magnify reveals more detailed structures with some antibodies. For example, the structure of foot processes of podocytes labeled by an anti-alpha-actinin 4 (ACTN4) antibody using the Expansion Pathology (ExPath) protocol is visible only in frozen samples[7], but not in FFPE kidney tissue sections. Similarly, expansion protocols have been used to image foot processes in PFA-fixed mouse[29, 30] and human samples[30]. However, these features and finer structures of the foot processes are readily apparent in the postexpansion staining with Magnify-processed FFPE kidney tissue sections (Fig. 2p and Supplementary Video 1). Additionally, we observed that conventional antigen retrieval protocols are not needed to achieve such an improvement as the denaturant-rich solution used during homogenization provides superior antibody labeling pre-expansion compared with conventional antigen retrieval buffers (Supplementary Fig. 3). This suggests expansion with Magnify may lead to improved accessibility of antibodies to epitopes, consistent with a previous report[31] (Fig. 2p–r, Supplementary Fig. 3 and Supplementary Videos 2–4).

**Fig. 3:**
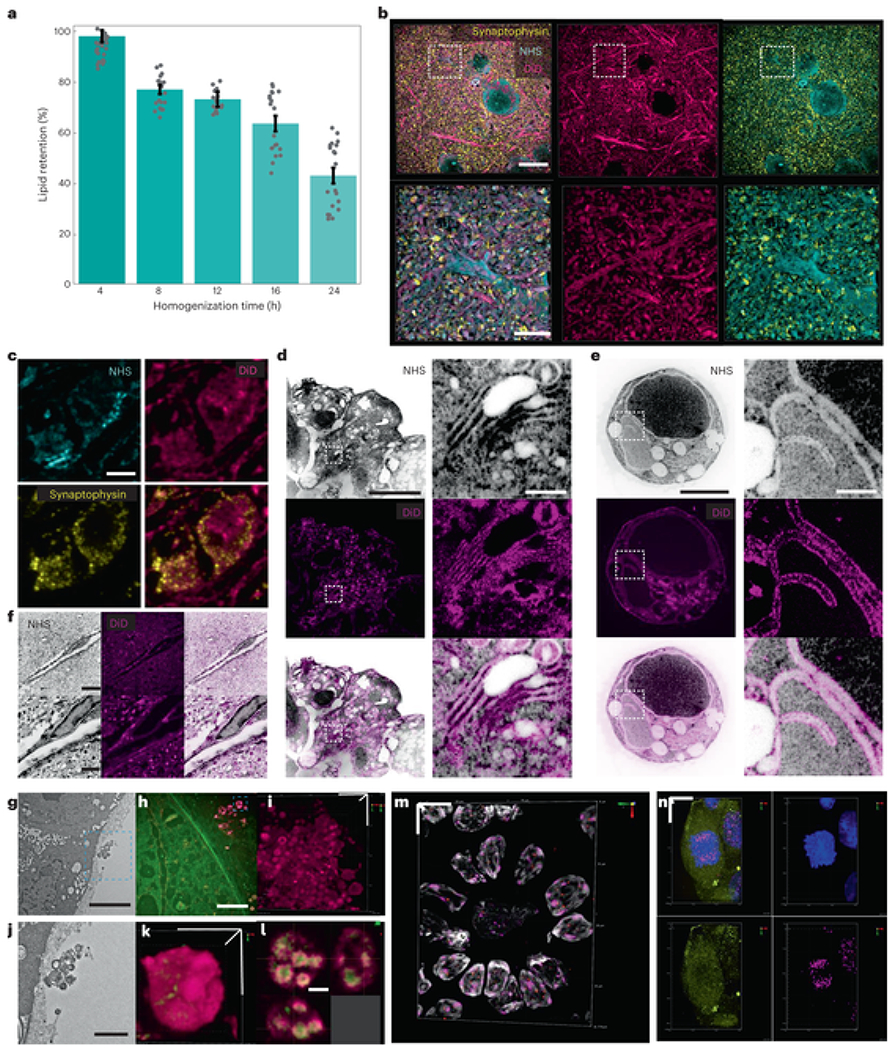
Imaging of proteins, nucleic acids and lipids in biological specimens with Magnify. **a**, Dotted bar chart showing lipid retention rate as a function of homogenization time in hot denaturing buffer. Error bars, s.e.m. **b**, Visualization of lipids in fully expanded Magnify-processed mouse brain. Top row, fully expanded mouse cortical neuron. Bottom row, zoom in of boxed region in top row. **c**, Visualization of a mitochondrion in Magnify-processed mouse brain with the lipophilic dye DiD. EF = 11×. **d**, Lipophilic dye staining of Golgi membranes in HEK-293FT cells expanded by Magnify. Right column, zoom ins of boxed area highlighting the Golgi body, **e**, Similar Magnify image of HEK-293FT cells as in **d**, but showing nuclear membrane labeling by DiD. EF = 9.22×. **f**, Lipophilic dye staining of blood vessels in mouse brain expanded by Magnify. EF = 10× in ddH_2_O. **g**, Electron micrograph of extracellular vesicles in human stem-cell-derived lung organoid, **h**, Two-color Magnify image of extracellular vesicles in the fully expanded human lung organoid with inverted look-up table. EF = 10.2×. Stain, green, Alexa Fluor 488 NHS ester; magenta, DiD. **i**, 3D reconstruction of **h. j**, Zoomed in view of boxed region in **g. k**, 3D reconstruction of the selected extracellular vesicle in **h** as indicated by the dashed blue box. **l**, Orthogonal view of the extracellular vesicle in **k**, showing complex internal structure. **m**, 3D reconstruction of confocal images of expanded human lymph node tissue labeled with DNA FISH probes against AKT1, Telomere (TelC) and human satellite 2. EF = 3.5× in 1× PBS. Gray, DAPI; green, AKT1; red, TelC; magenta, human satellite 2. **n**, 3D reconstruction of confocal images of expanded HEK-293FT cells. EF = 2.8× in 1× PBS. Blue, DAPI; green, RNA FISH probe against GAPDH; magenta, DNA FISH probe against human satellite 2. Scale bars (in biological scale), **b**, top, 5 μm, bottom, 2 μm; **c**, 250 nm; **d,e**, left, 5 μm, right, 1 μm; **f**, top, 5 μm, bottom, 2 μm; **g,h**, 3 μm; **i,j**, 1 μm; **k**, 500 nm. **l**, 200 nm; **m**, x, y and z: 7 μm; **n**, x, y and z: 10 μm.

ExM methods for imaging proteins[3, 4] and nucleic acids[11] have been increasingly adapted to accommodate imaging more types of biomolecules. While two ExM variants for visualizing lipids have been reported recently[9, 32], both require specimens labeled with lipid intercalating probes bearing an acrylamide functional group before in situ polymerization. We asked whether endogenous lipids can be retained by Magnify and found that paraldehyde-preserved mouse brain sections homogenized with denaturant-rich solution at 80 °C can be costained with traditional fluorescent lipophilic stains (such as DiO, DiI and DiD) and NHS esters of fluorescent dyes. We estimated up to 93% retention of lipids by comparing fluorescence intensity of DiD in mouse brain tissues both before and after Magnify processing at varying homogenization times (Fig. 3a, Supplementary Fig. 2 and Supplementary Note 1). Despite the relatively harsh homogenization conditions, we found the ultrastructure of lipids is conserved in neural processes (Fig. 3b ) mitochondria of the mouse brain (Fig. 3c), organelles in HEK-293FT cells (Fig. 3d–e), blood vessel membranes (Fig. 3f) and the myelin sheath of axons (Supplementary Fig. 3 ) of mouse brain. Using a ×40 (1.15 NA) water immersion objective lens, layer-like patterns can be partly resolved in some myelinated axons. Additionally, Magnify allows visualization of extracellular vesicles in human bronchial basal stem-cell-derived lung organoids (Fig. 3g–l), revealing complex internal structure (Fig. 3k–l ). It should be noted that the ability of Magnify to resolve any lipid-rich structure is dependent on the fixation method and the downstream sample preparation procedure. For example, the alcohol and xylene used in the dehydration step for embedding tissue in paraffin strip off lipids, precluding their imaging postexpansion with Magnify. Our results suggest that Magnify enables nanoscale observation of lipid membrane structures and their associated proteins using conventional confocal microscopes, indicating that Magnify can be a useful tool for the study of membrane biology and pathology in a wide range of biological specimens.

DNA fluorescent in situ hybridization (FISH) is commonly used to access gene amplification in tissues. We explored whether postexpansion DNA FISH is possible in expanded tissues processed with the Magnify protocol. We previously showed that the large size of traditional bacterial artificial chromosome-based FISH probes precludes efficient delivery to expanded, gel-embedded samples and thus small DNA oligo FISH probes are required[7]. Therefore, we synthesized and applied DNA FISH oligo probes (Supplementary Table 12) targeting *AKT1, CDH1* genes and several satellite sequences including telomere sequences[33] and centromere protein B box[34] to gel-embedded human lymph node and lung tissue sections homogenized with denaturant-rich solution at 80 °C for 48 h. We observed that all designed probes diffused into Magnify in both processed tissue types and hybridized with chromosomal DNA within 2 h at 45 °C (Fig. 3m and Supplementary Fig. 8). In addition, we observed that, with ProK homogenization, FFPE tissue sections can be processed, gel-embedded, homogenized and stained with DNA FISH probes and lectin stain within 8 h (Supplementary Fig. 8 and Supplementary Video 5 ), which could be useful in time-sensitive applications such as histopathological diagnoses. We also found that RNA can be preserved postexpansion with milder homogenization conditions. We demonstrated simultaneous DNA and RNA FISH in HEK-293FT cells homogenized with brief heat denaturation at 70 °C for 1.5 h (Fig. 3n). Thus, Magnify enables super-resolution FISH imaging of nucleic acids on tissues and cells.

**Fig. 4:**
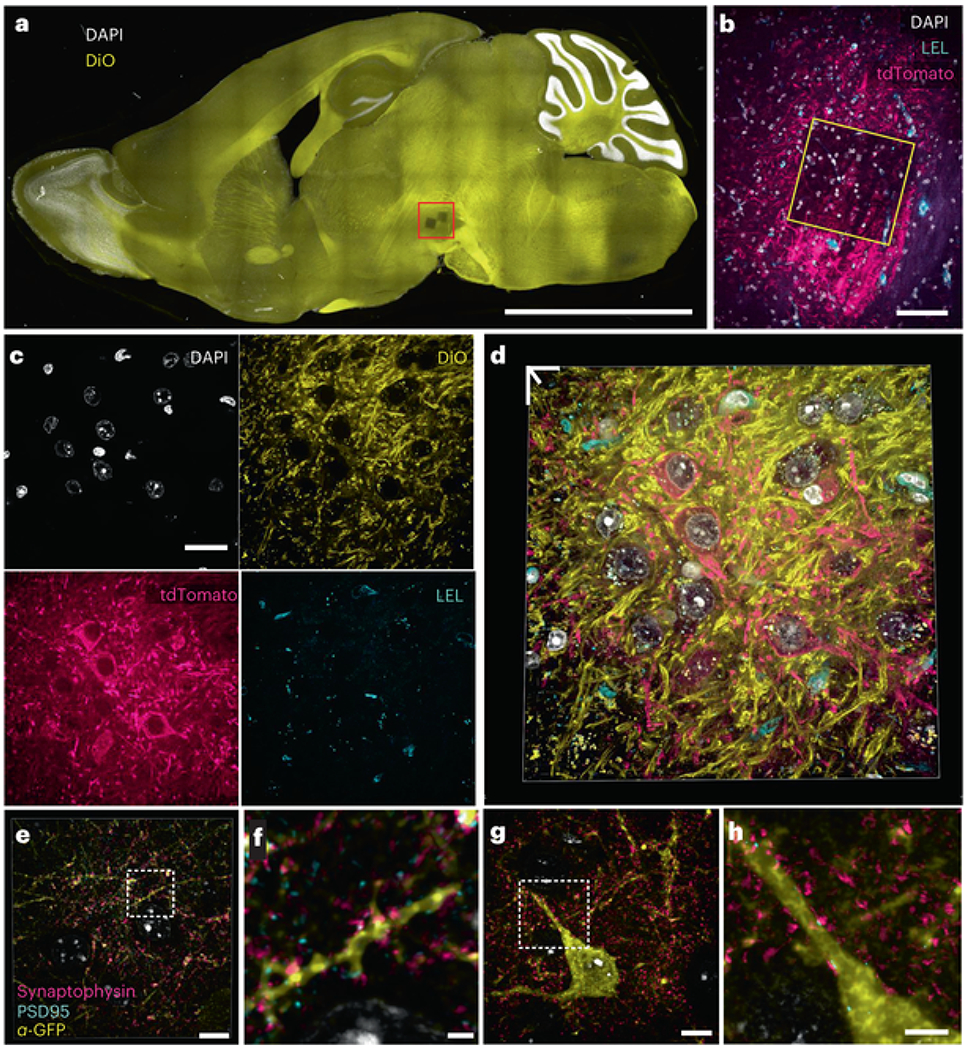
Visualization of endogenous fluorophores with Magnify. **a**, Maximum intensity projection of a sagittal mouse brain section expanded with Magnify-ProK. Yellow, DiO; White, DAPI. EF = 4.5× in PBS. **b**, Zoom in of boxed region in **a** showing imaged field in subsequent panels. Endogenous tdTomato can be seen in dopaminergic neurons in the ventral tegmental area. Cyan, *Lycopersicon esculentum* Lectin; magenta, crimson-tdTomato; white, DAPI. **c**, Zoom in of boxed region in **b** showing individual channels. **d**, 3D reconstruction of merged panels from **c. e**, 3D reconstruction of an SST cell in a fully expanded mouse cortex expanded with Magnify and homogenized with hot surfactant solution. Endogenous SST–GFP signal was recovered with an anti-GFP antibody applied postexpansion. Yellow, anti-GFP; cyan, PSD95; magenta, synaptophysin; white, DAPI. Synaptic markers close to GFP signal have been highlighted. EF = 9× in ddH_2_O. **f**, Zoom in of boxed region in **e** showing synapses on dendritic spines. **g**, Single *z* plane of a fully expanded SST neuron in mouse cortex from the same sample as **e. h**, Zoom in of boxed region in **e** showing synapses onto SST dendrite. Scale bars, **a**, 2.5 mm; **b**, 50 μm; **c**, 20 μm; **d**, 13 μm; (**e,g**, 5 μm; **f,h**, 2 μm. Scale bars are all in biological scale.

Transgenic animals expressing fluorescent proteins (FPs), as well as animals expressing FPs after viral gene delivery, are essential models used to visualize and study proteins and cells in intact tissues and whole organisms. To determine the compatibility of FP-expressing tissue with the Magnify gel framework, we first applied ProK to mouse brain (DAT-cre, injected with AAV5-Syn-FLEX-rc(ChrimsonR-tdTomato)) expanded with the Magnify gel, as is standard in other ExM protocols. Similar to the proExM protocol[3], FP tags were sufficiently preserved even after harsh protease treatment. Notably, endogenous lipids were also preserved (Fig. 4a–d). Although FP signals are preserved, the use of ProK destroys most endogenous proteins, making postexpansion staining of other proteins impossible. Conversely, treatment with a hot denaturant-rich buffer that preserves biomolecules results in a loss of FP signal. To explore the compatibility of the Magnify framework with FP-expressing transgenic animals, we used anti-FP antibodies to label FP-expressing cells in an SST-cre/Ai3 mouse brain after heat denaturation. We also immunostained synaptic pairs of synaptophysin and PSD95 to indicate excitatory synapses. Using a ×40 (1.15 NA) water immersion objective lens on a conventional confocal microscope, we can observe ultrafine dendritic spine morphology and partly resolved synaptic vesicles onto the postsynaptic densities of genetically targeted neurons after expansion (Fig. 4e–h and Supplementary Videos 6–8).

## Sub-20 nm resolution imaging enabled by Magnify–SOFI

As the Magnify framework is designed to allow for resolution of nanoscale structures without the need for specialized super-resolution imaging systems, a possible extension is the implementation of computational methods to further improve effective resolution. A relatively recent addition to the super-resolution arsenal, SOFI[19], is well positioned to be paired with ExM (Supplementary Note 4). SOFI can be used with a conventional microscope to improve resolution by relying on the independent temporal fluctuations of fluorophores to distinguish emitters. By using second -order cross-correlations, SOFI can improve the resolution by twofold beyond the diffraction limit with around 100 frames or fewer[19], as compared with tens of thousands with single-molecule localization microscopy (SMLM). By combining SOFI and 11-fold expansion, Magnify–SOFI can theoretically resolve around 13 nm features for a 1.15 NA diffraction-limited objective lens of around 280-nm (around 280/11/2), without any special modifications. Additionally, as background fluorescence is uncorrelated with the fluorescent signal over time, the signal-to-background ratio can be improved with SOFI over 100-fold[19]—a great benefit in expanded samples, as the expansion process dilutes the concentration of fluorophores.

**Fig. 5:**
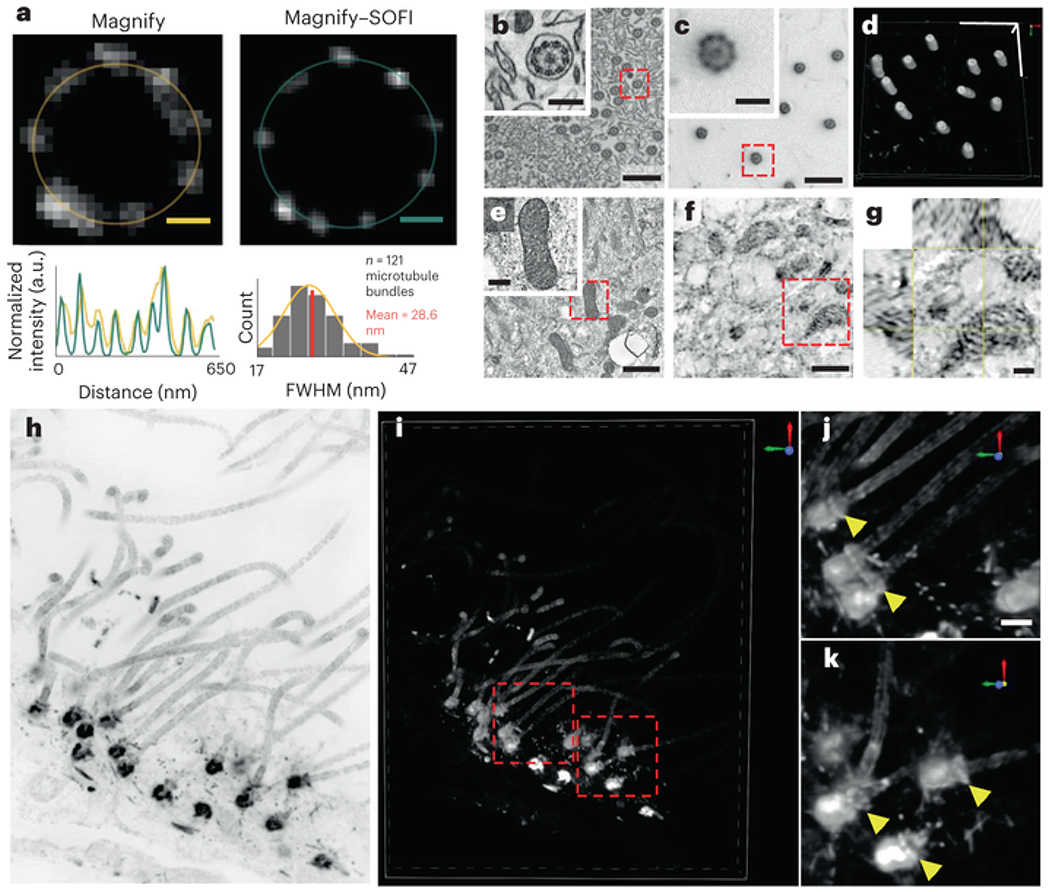
Magnify–SOFI visualizes ultrafine structures of cellular components. **a**, Comparison between Magnify and Magnify–SOFI. Top, Cross-section of a basal body in human bronchial basal stem-cell-derived lung organoid processed with Magnify (left) and Magnify–SOFI (right). Bottom left, radial intensity profiles of basal bodies indicated by yellow and green circles. Bottom right, histogram of microtubule bundle peak-to-peak distances. a.u., arbitrary units, **b**, Electron micrograph of cilia in human stem-cell-derived lung organoid; inset, zoom in view (red box). **c**, Confocal image of cilia from the same type of tissue as **b**, expanded by Magnify–SOFI and stained with Alexa Fluor 488-conjugated NHS ester. **d**, 3D reconstruction of cilia in **c. e**, Electron micrograph of mitochondria in the same organoid as in **b. f**, Confocal image of mitochondria from the same expanded organoid as in **e. g**, Orthogonal view of a mitochondria indicated by the red box in **e**. EF = 10× **h**, Maximum intensity projection of a Magnify–SOFI image stack of ependymal cilia and basal bodies from the ependymal cell lining in the adult mouse brain. **i**, 3D reconstruction of **h. j,k**, Zoomed in images of individual ependymal cilia in 3D as indicated by the dashed red boxes in **i**. Yellow arrows indicate distal appendages. EF = 10.5×. Scale bars, **a**, 50 nm; **b,c**, 800 nm, Inset, 200 nm; **d**, *x,y*, 1 μm, **z**, 410 nm; **e,f**, 800 nm, Inset, 200 nm; **g**, 200 nm; **h,i**, 500 nm; **j,k**, 250 nm; scale bars are all in biological scale.

To demonstrate the improved effective resolution, we used Magnify–SOFI to image nanoscale cellular machinery of human lung organoids derived from human bronchial basal stem cells. The hollow structure of cilia and basal bodies were well resolved (Fig. 5a,d) along with mitochondria cristae (Fig. 5e–g). The outer ring of cilia and basal bodies have been shown through EM to be composed of nine bundles of microtubules. While these subunits were often resolved with Magnify, it alone was insufficient to consistently resolve all nine bundles, particularly for slightly out-of-plane cilia and basal bodies. In contrast, Magnify–SOFI fully resolves these structures, and the distinctive ninefold symmetry is observed (Fig. 5a). Depending on the wavelength of fluorophore used, we estimated an effective resolution of around 14–17 nm using a parameter-free algorithm based on decorrelation analysis of Magnify–SOFI images[35] (Supplementary Fig. 5 and Supplementary Note 5). Additionally, Magnify–SOFI can be used to generate full three-dimensional (3D) reconstructions of these structures in tissue (Fig. 5h–k and Supplementary Video 9), which is challenging to achieve with EM.

## Optical evaluation of ciliopathies

Ciliopathies are a group of complex human genetic diseases characterized by both dysfunctional primary and motile cilia. Primary cilia are present on the apical surface of nearly every mammalian cell, and motile ciliopathies affect a broad range of important organs such as the airway, brain, middle ear, sperm and fallopian tubes[36]. The identification of factors regulating ciliogenesis and efficient characterization of cilia structure during ciliopathy can provide information critical for proper clinical diagnosis of cilia disorders and therapeutic development. The current methods for studying ciliogenesis include transmission electron microscopy (TEM), which is technically demanding, time-consuming and unable to easily provide 3D visualization of the full-length axoneme ultrastructure.

**Fig. 6:**
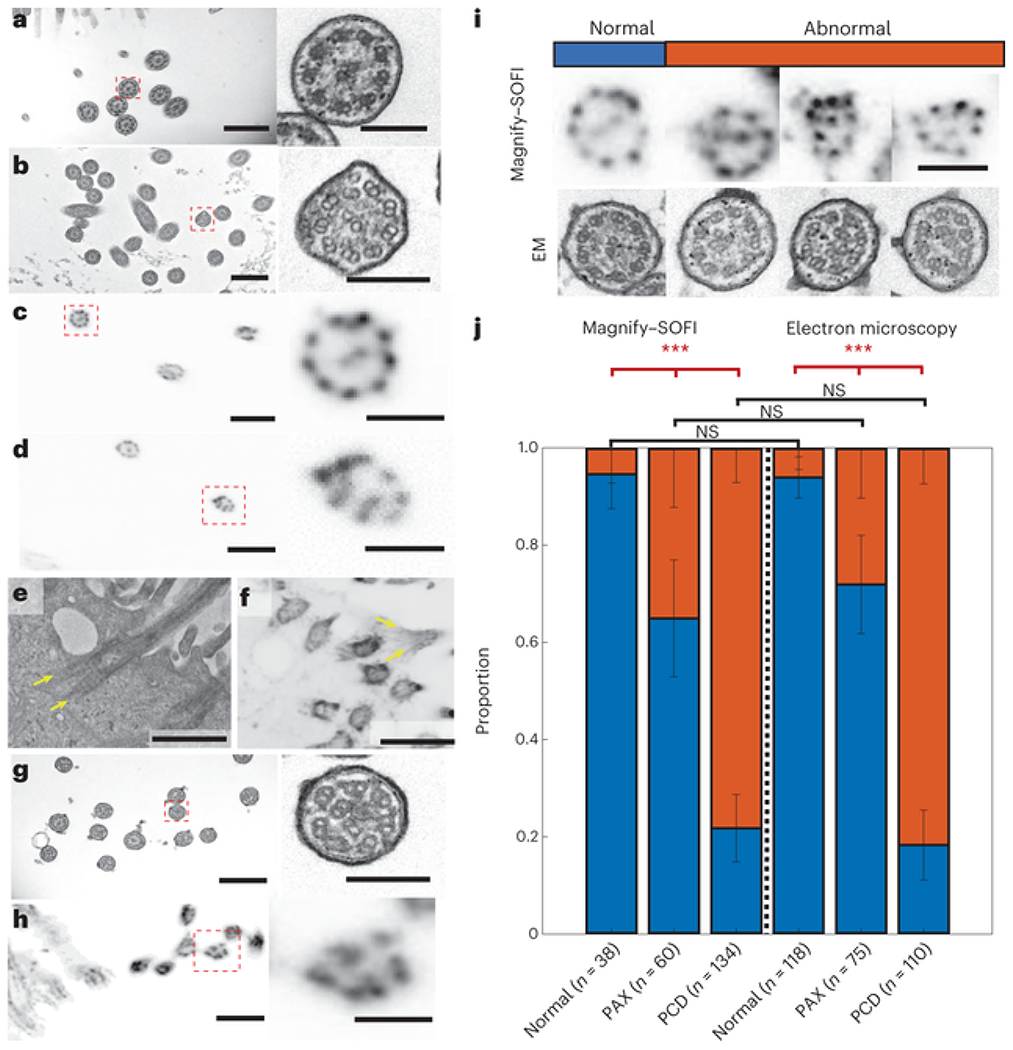
Magnify–SOFI visualizes subtle nanoscale drug-induced changes. **a**, Electron micrograph of cilia in normal human stem-cell-derived lung organoid. Right, zoomed in image as indicated by dashed red line boxes, **b**, similar to **a**, except the lung organoid was treated with Paclitaxel. **c**, Magnify–SOFI image of cilia from the same type of tissue as **a**, stained with Cy3-conjugated NHS ester. Right, zoomed in image as indicated by dashed red line boxes. **d**, Similar to **c**, except the lung organoid was treated with Paclitaxel. **e**, Electron micrograph of a basal body with prominent rootlets. **f**, Confocal image of corresponding basal body from the same type of tissue as **e**, expanded by Magnify and processed with SOFI. **g**, Electron micrograph of cilia in human stem-cell-derived lung organoid from CCDC39 mutation-bearing human bronchial basal stem-cell-derived lung organoids. Right, zoomed in image as indicated by dashed red line boxes. **h**, Magnify–SOFI image of cilia in similar tissue as **g. i**, Side-by-side comparison between Magnify–SOFI images (top) and electron micrographs (bottom) of cilia with and without defects. **j**, Stacked bar chart of proportions of normal and abnormal cilia in normal, taxol-treated (denoted as PAX samples), and CCDC39 mutation-bearing human bronchial basal stem-cell-derived lung organoids (denoted as PCD samples). Three bars on the left were based on Magnify–SOFI images while the three on the right were based on electron micrographs. Error bars, s.e.m. Scale bars, **a**, 600 nm; **b,c**, 100 nm; **d**, 800 nm; **e,f**, 100 nm; **g,h**, 800 nm; **i**, 200 nm; same for both Magnify–SOFI and EM images. Scale bars are all in biological scale.

Taxol is a chemotherapeutic agent that can bind to β-tubulin subunits to stabilize microtubules, preventing their dissociation via Ca^2+^ or cold temperature in vitro, halting microtubule dynamics that play am important role in mitosis, motility and cell growth and survival[37, 38, 39]. Taxol can also cause structural abnormalities in cilia, including supernumerary microtubules, disorganized microtubule doublets and inhibition of basal body migration towards the cell surface[40, 41]. Compared with the normal state (Fig. 6a), human lung organoids treated with 20 μM Taxol showed malformed ciliary structures through EM imaging, with supernumerary and misplaced microtubules (Fig. 6b ). We asked whether Magnify–SOFI could be used to identify these malformed ciliary structures on a conventional optical microscope. We found that, in contrast to control human lung organoids, which exhibited consistent ninefold symmetry (Fig. 6c), organoids treated with Taxol exhibited deformities such as misplaced microtubule doublet subunits that were seen with EM (Fig. 6d). We also observed basal bodies with two split rootlets in both EM and Magnify images of Taxol-treated organoids. (Fig. 6e,f).

We asked whether Magnify–SOFI could characterize structural defects of cilia under various pathophysiological conditions, which provides key information for understanding the fundamental mechanisms of ciliopathy and further demonstrates the utility of Magnify for studying disease pathogenesis. We performed Magnify–SOFI imaging on cilia of apical-out airway organoids[42] engineered from healthy stem cells with and without 20 mM Taxol treatment and patient stem cells carrying variants in the *CCDC39* gene (Fig. 6g,h). These variants cause inner dynein arm defects and axonemal disorganization in cilia[43, 44]. They are associated with primary ciliary dyskinesia (PCD)[45, 46]—a group of genetic disorders with abnormal motile cilia ultrastructure and function. Despite lower resolution than EM, Magnify–SOFI captured malformed ciliary structures with only a diffraction-limited confocal microscope (Fig. 6i). Further quantification of normal and defective cilia ultrastructure revealed distinct patterns in populations under three conditions: normal, Taxol-treated and *CCDC39* gene variants. There was no statistical difference between Magnify–SOFI and EM (Fig. 6j). Additionally, Magnify–SOFI showed similar performance to EM in capturing statistically significant intraclass variability among the three conditions (Fig. 6j ). Thus, Magnify–SOFI has the potential to deliver insights into the biology and disruptions to the cilia structures responsible for cilia dysfunction and to broaden our knowledge of the pathogenesis of human lung diseases to improve lung health.

## Discussion

We developed a simple and versatile ExM method that preserves biomolecules and enables volumetric imaging of their nanoscale organization in a wide range of intact biological specimens. Magnify does not require a special fixation method to anchor biomolecules[5], dedicated anchoring[6, 7, 11, 28] or custom linkers to preserve biomolecules such as lipids[8, 9] and is capable of expanding tissues inaccessible by other enzyme-free ExM methods[5, 10]. The hydrogel-tissue hybrids preserve the nanoscopic features and cellular connectivity after up to 11-fold expansion, which can be imaged directly using commercial labeling agents applied either before or after expansion. Magnify preserves protein epitopes and other biomolecules allowing for post-Magnify staining, which substantially reduces the linkage errors caused by the size of labeling agents. For example, tubulin measurements taken with Magnify (Supplementary Fig. 6) are closer to EM than unexpanded stained samples[47]. For new applications, validation of labeling agents is still recommended. When combined with SOFI, Magnify achieves effective resolutions approaching top performers of super-resolution light microscopy, yet does not require special equipment or fluorophores, and is easy to implement. While we did not attempt to demonstrate all possible combinations of biomolecules in the same tissue here, we did demonstrate imaging of four different molecules (DNA, FPs, endogenous lipid and carbohydrates) in the same sample (Fig. 4c,d), as well as dual protein-lipid staining in both PFA-fixed tissue and cell culture. With optimization, it is possible to image four different biomolecules in one PFA-fixed sample with sub-20 nm resolution using Magnify–SOFI. This could be used to study how higher-order chromatin structures are influenced by the nuclear envelope in health and disease states, which requires nanoscale imaging of DNA, histone proteins and lipids.

Due to the small sizes and fast diffusion rate of monomers, Magnify may have future applicability to thick tissues and whole organisms. Magnify would be readily adaptable to generating nanoscale whole organ datasets, which currently rely on either lower resolution tissue clearing methods (CLARITY, CUBIC-X, iDISCO, and so on), or serial block-face EM. Additionally, even larger EFs could be achieved if integrated with other strategies such as iterative expansion microscopy (iExM)[15] or semi-interpenetrating polymer networks[10]. With large-volume imaging modalities and objectives of 6–8 mm working distance (for example, the Olympus ×25 0.9 NA and the Leica ×25 1.0 NA), tissues up to 0.8 mm thick can be fully expanded with Magnify and imaged. Adjusting the size of samples based on external ionic concentrations allows macroscale mapping to be performed in the shrunken state. Expansion in a lower-salinity buffer can allow exploration of smaller scale details all on a conventional imaging system, enabling researchers to generate tissue atlases without the need for specialized equipment. This would decrease imaging time as regions of interest can be identified on the same system before fully expanding samples. The use of fiducial markers to realign images along with custom perfusion systems to expand and shrink samples can facilitate easy coregistration of images.

Because Magnify is a chemical strategy that does not rely on complex optics, it offers a flexible framework that can be adapted to a range of imaging modalities, gel chemistries and other ExM strategies. ExM methods have already demonstrated compatibility with existing super-resolution techniques including structured illumination microscopy[48], stimulated emission depletion microscopy[49] and Single-molecule localization microscopy[47, 50]. Magnify-processed samples could also be implemented in multiplexing strategies, such as DNA barcoding[51] and serial immunostaining[52]. Signal amplification methods, such as FRACTAL[52] or immunoSABER[53], may be applied to image sparse biomolecules and compensate signal dilution from expansion. Magnify may also enable nanoscale imaging with other modalities, such as stimulated Raman scattering and various mass-spectrometry-based imaging techniques. Finally, Magnify may be implemented in high-content imaging systems, where large datasets can be generated to explore the effect of drug treatments and disease-associated changes on the nanoscale configuration of biomolecules in culture cells and tissue models.

## Methods

### Reagents and antibodies

Detailed information for reagents and antibodies is shown in Supplementary Tables 4, 5 and 11.

### Human samples

The human pathology specimens were purchased from US Biomax catalog nos. HuFPT072 (normal human kidney cortex), UNC242 (universal control tissue array), LY241g (lymph node and tonsil tissue array), MC245c (multiple organ tumor array), Lc241I (lung cancer tissue array) and HuFPT210 (lymph node).

### Mouse brain samples

All mouse brain expansion experiments were performed using C57BL/6 mice except where otherwise noted. C57BL/6 mice were deeply anesthetized with ketamine/xylazine before transcardial perfusion with 20 ml 4% PFA in 1× PBS. Brains were harvested and postfixed in 4% PFA in 1× PBS overnight at 4 °C. Tissue was cryoprotected by incubating in 30% (w/v) sucrose in 1× PBS at 4 °C until the brain sank (usually 16 h/overnight). Brains were sectioned in 30-μm slices (either coronally or sagittally) using a freezing microtome and stored at 4 °C in glycerol solution (30% (v/v) glycerol and 30% (v/v) ethylene glycol in 1× PBS). Figure 4a–d was generated using DAT-cre mice with C57 black background implanted with headposts and injected with two viruses (AAV2/5-hSynapsin1-FLEx-axon-GCaMP6s and AAV5-Syn-FLEX-rc(ChrimsonR-tdTomato)) in the ventral tegmental area. DAT-cre mice were deeply anesthetized with isoflurane before transcardial perfusion with 20 ml 4% PFA in 1× PBS. Brains were harvested and postfixed in 4% PFA in 1× PBS overnight at 4 °C. Tissue was cryoprotected by incubating in 30% (w/v) sucrose in 1× PBS at 4 °C until the brain sank (usually 16 h/overnight). Brains were sectioned in 80-μm slices (either coronally or sagittally) using a cryostat and stored at 4 °C in glycerol solution (30% (v/v) glycerol and 30% (v/v) ethylene glycol in 1× PBS). Figure 4e–h was generated using somatostatin (SST)-cre/Ai3 mouse brain tissue (SST-Cre mouse line, Jax 013044, crossed to Ai3 (Jax 007903)).

### Culture of airway basal cells

Bronchus-derived human airway basal stem cells (hABSCs) were purchased from Lonza. Additional hABSCs were obtained from surgical excess of deidentified tissues of healthy lung donors or donors carrying variants in *CCDC39* gene (c.830_831delCA (p.Thr277Argfs*3) and c.1871_1872delTA (p.Ile624Lysfs*3), with permission of the Institutional Review Board at Washington University in Saint Louis (IRB ID 201103213). The hABSCs were cultured in 804G-conditioned medium coated culture vessels in bronchial epithelial cell growth medium (BEGM): 1 μM A8301, 5 μM Y27632, 0.2 μM of DMH-1 and 0.5 μM of CHIR99021 (ref. [54]) at 37 °C with 5% CO_2_.

### Differentiation of airway basal cells into airway organoids

A 96-well tissue culture plate was coated with 40% (v/v) growth factor reduced (GFR) Matrigel in PneumaCult-ALI Maintenance Medium. The NHBEs were resuspended in 40% (v/v) GFR Matrigel in PneumaCult-ALI Maintenance Medium and added to the coated wells; 100 μl PneumaCult-ALI Maintenance Medium was placed in the wells and changed every other day. The cultures were maintained at 37 °C with 5% CO_2_ for 21 days.

### Treating airway organoids with paclitaxel

The 21- to 28-day-old organoids were treated with paclitaxel (Taxol), diluted in PneumaCult-ALI Maintenance Medium to a final concentration of 20 μM, for 24 h. The control groups were treated with an equal concentration of dimethyl sulfoxide (DMSO) diluted in PneumaCult-ALI Maintenance Medium.

### Culture of HEK-293FT cells

HEK-293FT cells (ThermoFisher Scientific, catalog no. 51-0035) were grown in Dulbecco’s modified Eagle medium supplemented with 5% fetal bovine serum, 4.5 g l^−1^ d-glucose, 110 mg l^−1^ sodium pyruvate, 6 mM l-glutamine, 0.1 mM nonessential amino acids and 500 μg ml^−1^ Geneticin selective antibiotic. Cells were not authenticated or tested for mycoplasma contamination. All cells were maintained at 37 °C in a humid 5% CO_2_ atmosphere. Cells were grown on no. 1.5 cover glass treated with 1–2.5 μg cm^−2^ fibronectin. Cells were then fixed with 4% PFA and 0.1% glutaraldehyde in 1× PBS for 10 min at room temperature (RT).

### Tissue-section recovery and heat treatment

FFPE clinical samples were washed in the following solutions two times for 3 min each at RT: xylene, 100% ethanol, 95% ethanol, 70% ethanol, 50% ethanol and doubly deionized water. For samples that were stained before gelation, tissue slides were placed in 20 mM sodium citrate solution (pH 8) or 1% w/v SDS, 8 M Urea, 25 mM EDTA, 2× PBS (pH 9) at 100 °C. The container was transferred to a 60 °C incubator for 30–45 min.

### Pre-expansion immunostaining of FFPE samples

After heat treatment, samples were blocked with SuperBlock Blocking Buffer in PBS for 1 h at 37 °C followed by incubation with primary antibodies diluted to approximately 1 μg ml^−1^ in staining buffer (9× PBS/10% TritonX/10 mg l^−1^ heparin) overnight at RT or for 1 h at 37 °C. Samples were washed at least three times with washing buffer (1× PBS/0.1% TritonX-100) at RT for at least 10 min. Samples were then incubated in staining buffer with the corresponding secondary antibodies diluted to approximately 1 μg ml^−1^ together with 300 nM 4,6-diamidino-2-phenylindole (DAPI) for at least 3 h at RT or for 1 h at 37 °C. Samples were then washed at least three times with washing buffer for at least 10 min each. After washing, samples used for pre-expansion imaging for protein retention estimation were stained with NHS-ATTO-488 in 1× PBS for 30 min at RT and then washed with 1× PBS before imaging. NHS-stained samples were not gelled.

### Pre-expansion immunostaining of mouse brain samples

Mouse brain samples were taken from glycerol solution and washed three times for 10 min each in 1× PBS at RT before permeabilization in 1% TritonX-100 in 1× PBS at RT. Samples were then incubated with primary antibodies in 1% TritonX-100 for 15 min in 1× PBS overnight at 33 °C. Samples were washed three times for at least 20 min each in 1× PBS at RT before incubation in 1% TritonX-100 in 1× PBS with the corresponding secondary antibodies for 3 h at 37 °C and were washed with 1× PBS before imaging. Samples used for total protein content estimation were stained with NHS-ATTO-488 or NHS-ATTO-532 diluted to 1:25–1:150 for 3 h at RT in 1× PBS. Samples used for total lipid content estimation were stained with DiO or DiD for 72–96 h in 0.1% TritonX-100 at RT and washed at least three times with 1× PBS.

### Pre-expansion immunostaining of HEK-293FT cells

After fixation, HEK-293FT cells were permeabilized for 10 min in 1% TritonX-100 in 1× PBS at RT followed by blocking with SuperBlock Blocking Buffer in PBS for 10 min at RT. Samples were then incubated in staining buffer with mouse anti-αTubulin to approximately 1 μg ml^−1^ together with 3 h at RT. Samples were then washed at least three times with washing buffer for at least 10 min each followed by staining with AF488 AffiniPure Fab fragment donkey anti-mouse for at least 1 h at RT.

### In situ polymer synthesis of FFPE samples with Magnify

A monomer solution made of 4% DMAA (v/v), 34% SA (w/v), 10% AA (w/v), 0.01% Bis (w/v), 1% NaCl (w/v) and 1× PBS was prepared and stored at 4 °C before synthesis. Immediately before gelation, the chemicals 4HT, APS, TEMED and methacrolein were added to a final concentration of 0.2–0.25% (w/v) APS, 0–0.25% (v/v) TEMED, 0.001% 4HT (w/v) and 0.1–0.25% (v/v) methacrolein. The solution was vortexed, and tissue slides were incubated with the gelling solution for 30 min at 4 °C to allow the monomer solution to diffuse into the tissue while preventing premature gelation. A gelling chamber was then constructed around the tissue, consisting of spacers cut from no. 1.5 cover glass and a glass slide, placed back side down, on top. The samples were incubated overnight in a humidified container at 37 °C to complete gelation.

### In situ polymer synthesis of mouse brain, human stem-cell-derived organoids and HEK-293FT cell samples with Magnify

Magnify gel monomer solution was prepared as described above. Before gelation, samples were placed into custom gelling chamber consisting of two spacers cut from no. 1.5 cover glass adhered to the uncoated back of a microscope slide (four spacers used for HEK-293FT cells). Excess PBS around the tissue was absorbed with a Kimwipe and sections were allowed to air dry partially on the slide. Immediately before gelation, the chemicals 4HT, TEMED, APS and methacrolein were added to the gel monomer solution to a final concentration of 0.25% (w/v) APS, 0.001% 4HT (w/v, mouse brain and organoid only), 0.04% TEMED (v/v) and 0.1% (v/v) methacrolein, adding TEMED and APS last to prevent premature gelation. Tissue was incubated in gelling solution for 30 min at 4 °C to allow the monomer solution to diffuse into the tissue (mouse brain and organoid only). A glass microscope slide was placed back side down over the gelling chamber and the samples were incubated overnight in a humidified container at 37 °C to complete gelation.

### Sample digestion and expansion with Magnify

After gelation, the glass slide cover was removed from the gelling chamber, blank gel surrounding the tissue was trimmed from the samples and the tissue was cut into smaller pieces if necessary. Samples were then incubated in homogenization buffer (1–10% w/v SDS, 8 M Urea, 25 mM EDTA, 2× PBS, pH 7.5 at RT) for 24-72 h at 80 °C with shaking. Homogenized samples were then washed three times with 1× PBS at RT, followed by at least three washes in 1% decaethylene glycol monododecyl ether (C_12_E_10_)/1× PBS or 1% PBST at RT or 60 °C to remove remaining SDS. Expanded brain samples were additionally incubated in 1% decaethylene glycol monododecyl ether (C_12_E_10_)/1× PBS at 60 °C for 1 h. Samples were finally washed an additional three times for at least 10 min each with 1× PBS at RT and stored in 1× PBS containing 0.02% sodium azide at 4 °C.

### Protease digestion of ExPath and Magnify mouse brain samples

After gelation, blank gel surrounding the tissue was trimmed from the samples. Samples were then incubated in the ExPath homogenization buffer (50 mM Tris (pH 8), 25 mM EDTA, 0.5% (w/v) TritonX, 0.8 M NaCl) with Proteinase K diluted by 1:400 (final concentration 2 U ml^−1^). Mouse brain samples were then homogenized at RT for 1–2 h. Homogenized samples were then washed three times with 1× PBS at RT.

### Postexpansion immunostaining of mouse brain samples

Expanded samples were taken from storage in 1× PBS at 4 °C and washed three times for 10 min each in 1× PBS at RT. Samples were then incubated with primary antibodies in 1% TritonX-100 in 1× PBS overnight at RT. Samples were washed three times for at least 20 min each in 1× PBS at RT before incubation in 1% TritonX-100 in 1× PBS with the corresponding secondary antibodies for 3 h at RT. Before imaging, samples were washed with 1× PBS. Samples used for total protein content estimation were shrunk in polyethylene glycol (PEG 200) three times for 10 min each before staining with NHS-ATTO-488 or NHS-ATTO-532 diluted to 1:25–1:150 for 3 h at RT in 1× PBS. Samples used for total lipid content estimation were stained with DiO or DiD diluted to 1:200 for 72–96 h in 0.1% TritonX-100 at RT and washed at least three times with 1× PBS. After staining, samples were washed in water for at least 10 min. This was repeated until the sample was fully expanded—at least three exchanges of water. Samples stained with lipophilic dyes were imaged as close to the time of expansion as possible to prevent dissociation of the dye in water.

### Postexpansion immunostaining of FFPE samples and expansion

After homogenization and washing, kidney samples used for comparing ExM protocols (MAP, X10, ExPath) were stained with 1:1,000 DAPI and 1:250 wheat germ agglutinin 640 in 1× PBS for 3 h at RT and washed three times in 1× PBS followed by staining NHS-ATTO-488 in 1× PBS diluted to 1:250 for 30 min. Magnify-processed samples were stained with respective primary antibodies diluted to approximately 1 μg ml^−1^ in staining buffer (9× PBS/10% TritonX/10 mg l^−1^ heparin) overnight at RT. Samples were then washed three times with washing buffer (1× PBS/0.1% TritonX-100) at RT for at least 10 min. Samples were then incubated in staining buffer with the corresponding secondary antibodies diluted to approximately 1 μg ml^−1^ together with 300 nM DAPI for at least 3 h at RT. Samples were then washed at least three times with washing buffer for at least 10 min. After staining, samples were washed in water for at least 10 min. This was repeated until the sample was fully expanded—at least three exchanges of water.

### Postexpansion immunostaining of HEK-293FT samples and expansion

After homogenization and washing, HEK-293FT cells used for distortion analysis measurements were stained with approximately 1 μg ml^−1^ rabbit anti-αTubulin in staining buffer (9× PBS/10% TritonX/10 mg 1^−1^ heparin) overnight at RT. Samples were then washed three times with washing buffer (1× PBS/0.1% TritonX-100) at RT for at least 10 min. Samples were then incubated in staining buffer with AF488 AffiniPure Fab fragment donkey anti-rabbit diluted to approximately 1 μg ml^−1^ for at least 1 h at RT. Samples were then washed at least three times with washing buffer for at least 10 min. After staining, samples were washed in water for at least 10 min. This was repeated until the sample was fully expanded—at least three exchanges of water. Previously unstained samples used for pan-protein and lipid imaging were first washed three times in 2× SSC (300 mM NaCl, 30 mM sodium citrate, pH 7.0)/1% Tween 20 and then stained with 1:250 Cy3 NHS ester at 4 °C overnight. Samples were then washed at least three times with washing buffer for at least 10 min. Samples were then stained with DiD diluted to 1:200 overnight in 0.1% TritonX-100 at RT and washed at least three times with 1× PBS.

### Postexpansion FISH

For Magnify samples being processed for DNA FISH probing, digested gel samples were placed in hybridization buffer made of 1× PBS, 5% (v/v) TritonX, 10% (v/v) ethylene carbonate containing 10 pM of FISH probes against human gene *AKT1* and *CDH1* (Supplemental Table 12), human satellite 2 (/5ATTO647N/TCGAGTCCATTCGATGAT, Integrated DNA Technologies), human alpha satellite (/5ATTO647N/ATGTGTGCATTCAACTCACAGAGTTGAAC, Integrated DNA Technologies) or telomere TelC (/5ATTO550N/CCCTAACCCTAACCCTAACCCTAACCC, Integrated DNA Technologies) and CENP-B box motif (/5ATTO647N/ATTCGTTGGAAACGGGA, Integrated DNA Technologies) and 1 μg ml^−1^ wheat germ agglutinin conjugated with Alexa Fluor 488. The mixtures were then incubated at 37 °C for 2 h. The samples were washed with stringency wash buffer made of 1× SSC (150 mM NaCl, 15 mM sodium citrate, pH 7.0) at 37 °C for 15 min, followed by washes with 2× SSC at 37 °C three times for 10 min each. Finally, the gel samples were washed with 1× PBS several times at RT (5 min each) before imaging.

### Imaging

Fluorescence imaging was performed using a Nikon Eclipse Ti2 epifluorescence microscope equipped with a CSU-W1 spinning disk confocal module and an Andor v.4.2 Zyla sCMOS camera. The system was controlled by NIS-Elements AR v.5.21.03 64-bit software. Images were taken using the following Nikon objectives: CFI Plan Apo Lambda ×4 (0.2 NA), CFI Plan Apo Lambda ×10 (0.45 NA), CFI Apo LWD Lambda S ×20 WI (0.95 NA), CFI Apo LWD Lambda S ×40 WI (1.15 NA) and CFI Plan Apo Lambda ×60 Oil (1.4 NA).

### SOFI imaging

Samples were fully expanded in ddH_2_O in a glass-bottom six-well plate or custom imaging dish for larger samples. To prevent drift, samples were covered with plastic wrap. Alternatively, imaging glass may be coated in poly-l-lysine solution before placement of the gel. SOFI images were taken with either a CFI Apo LWD Lambda S ×40 WI (1.15 NA), CFI Plan Apochromat VC ×60 C WI (1.2 NA) or CFI Plan Apo Lambda ×60 Oil (1.4 NA) objective, with an optional ×1.5 magnification. Each SOFI image consisted of 50–100 frames per*z* plane with 50–200 ms exposure time per frame.

### SOFI image processing

SOFI images were processed using custom MATLAB code. Images were corrected for drift and intensity, cropped and deconvolved (Lucy-Richardson method) after 3D cross-correlation SOFI. For more information, refer to Supplementary Note 4.

### Measurements of EF for FFPE samples

EFs for comparison of ExPath, X10 and Magnify protocols were estimated using average nuclear surface area of kidney samples. Images of DAPI-stained kidney samples were obtained at ten times magnification before gelation and after homogenization and expansion. Nuclear surface areas were determined using the Analyze Particles tool in FIJI/ImageJ after image thresholding and binarization. To calculate the linear EF, the square root of the ratio of the average postexpansion nuclear surface area to average pre-expansion surface area was calculated. For specimens with pre-expansion images, immunostained FFPE samples were imaged at four times and ten times magnification. After gelling and homogenization, expanded tissue pieces were imaged at four times and ten times magnification. Regions of interest in postexpanded images were matched to pre-expansion regions of interest and the distance measurement tool in NIS-Elements or FIJI/ImageJ was used to measure feature sizes in both pre- and postexpansion images.

### Quantification of protein retention in FFPE samples

After antigen retrieval, FFPE kidney samples stained with NHS-ATTO-488 were imaged at four times and ten times magnification. Due to the long incubation time necessary to homogenize the kidney samples, a separate set of FFPE kidney samples were gelled without NHS ester staining. Samples were homogenized for 60 h at 80 °C, washed in washing buffer and stained with NHS-ATTO-488. After staining, samples were washed in washing buffer and incubated in 10× PBS to achieve an EF closer to the pre-expansion images. Samples were imaged at four times and ten times magnification using the same parameters as the pre-expansion images. Mean fluorescent intensity was calculated over a region of interest (ROI) drawn in NIS-Elements after background subtraction for both pre- and postexpansion images. Mean intensities were averaged over technical replicates, and postexpansion data was scaled by the cubed linear EF to account for volume differences.

### Quantification of protein and lipid retention in mouse brain samples

Mouse brain sections were stained with NHS-ATTO-532 and DiD and were imaged at ten times magnification. One sample was stained with NHS-ATTO-532 and DiD with no homogenization, while the rest of the samples were homogenized for varying times in either ProK or hot surfactant, washed and stained with NHS-ATTO-532 and DiD, while the other was stained with NHS-ATTO-532 and DiD with no homogenization. Samples were then incubated in 10× PBS to achieve an EF closer to the pre-expansion images and were imaged at ten times magnification using the same parameters as the pre-expansion images. Mean fluorescent intensity was calculated over an ROI drawn in NIS-Elements after background subtraction for both pre- and postexpansion images. Mean intensities were averaged over technical replicates, and postexpansion data were scaled by the cubed linear EF to account for volume differences.

### Quantification of protein retention in human lung organoid samples

Human lung organoid samples were stained with NHS CF 555 and placed in homogenization buffer for 5 h and then imaged at four times magnification. Samples were then homogenized for 9 h at 80 °C, washed and then incubated in 10× PBS to achieve an EF closer to the pre-expansion images and were imaged at four times magnification using the same parameters as the pre-expansion images. Mean fluorescent intensity was calculated over an ROI drawn in NIS-Elements after background subtraction for both pre- and postexpansion images. Mean intensities were averaged over technical replicates, and postexpansion data were scaled by the cubed linear EF to account for volume differences.

### Comparison of pre-expansion SOFI images and postexpansion images of FFPE samples

Pre-expansion SOFI images were taken using a CFI Plan Apo Lambda ×60 Oil (1.4 NA) objective. Each SOFI image consisted of 50 frames per *z* plane with 50–200 ms exposure time per frame. Time series of all channels were taken at each *z* plane before moving to the next. SOFI images were processed using custom MATLAB code.

### Measurement error quantification

Error was quantified using previously described methods for distortion vector field calculation and root mean square (RMS) error calculation [1, 7]. Briefly, pre-expansion SOFI images were taken at a single *z* plane at ×60 magnification and several *z* planes for the same fields of view (FOVs) were obtained postexpansion at ×40 magnification as precise matching of *z* planes to pre-expansion images can be challenging. To match postexpansion *z* planes, scale invariant feature transform (SIFT) key points were generated for all possible combinations of pairs of the pre-expansion images and postexpansion *z* projections. Because the sample expands along the *z* axis and different imaging conditions were used, one pre-expansion *z* plane should correspond to one postexpansion *z* projection from 8 to 25 *z* planes. SIFT key points were generated using the VLFeat open-source library and filtered by random sample consensus (RANSAC) with a geometric model that only permits rotation, translation and uniform scaling. The pair of pre-expansion and postexpansion images with the most SIFT key points were then used for image registration by rotation, translation and uniform scaling, as well as calculation of EFs and distortion vector fields. By subtracting the resulting vectors at any two points, distance measurement errors could easily be sampled, and the RMS error for such measurements was plotted as a function of measurement length from at least three technical replicates.

### Transmission electron microscopy

Brain samples were processed and organoids were processed as pellets, as described previously[55, 56]. All images were taken on a JEOL JEM 1400 Flash transmission electron microscope at 80 kV.

### Statistics and reproducibility

All experiments were carried out at least three times independently, unless otherwise noted in the figure legends. All data are expressed as mean ± s.e.m., unless otherwise noted. The following sample sizes were used: Fig. 1e : Kidney samples were anchored with 0.25% methacrolein (*n* = 13 technical replicates from one kidney slice) or AcX (*n* = 14 technical replicates from one kidney slice) and homogenized in hot surfactant for 60 h. Brain samples were anchored with 0.1% mecharolein (*n* = 20 technical replicates from two brain slices) or AcX (*n* = 20 technical replicates from two brain slices) and homogenized for 8 h, respectively. Kidney samples anchored with 0.05% methacrolein (*n* = 12 technical replicates from one kidney slice) or AcX (*n* = 9 technical replicates from one kidney slice) and homogenized in ProK for 3 h and brain samples were anchored with 0.1% mecharolein (*n* = 20 technical replicates from two brain slices) or AcX (*n* = 20 technical replicates from two brain slices) and were homogenized in ProK for 2 h. Figure 1f: FFPE human kidney sections anchored with 0.25% methacrolein (*n* = 13 technical replicates from one kidney slice), mouse brain sections anchored with 0.1% methacrolein (*n* = 20 technical replicates from two brain slices) and human lung organoid samples (*n* = 13 technical replicates from two organoids) anchored with 0.1% methacrolein were homogenized in hot surfactant for 60 h, 4 h and 8 h, respectively. All replicates are technical replicates from the same organs. Figure 1i: Synapse pair distances were taken from five regions from the same expanded section of mouse brain. Number of synaptic measurements: M1 L5, *n* = 265. M1 L6, *n* = 227. S1 L4, *n* = 284. S1 L6, *n* = 274. DMS, *n* = 263. NAc, *n* = 268. Values are reported as mean ± s.d. Figure 2c,d : *n* = 5 technical replicates from the same organ slice. Figure 2h,i : *n* = 4 technical replicates from the same organ slice. Figure 3a : Brain samples were anchored with 0.1% mecharolein and homogenized in hot surfactant for 4 h, 8 h, 12 h and 16 h (*n* = 20 technical replicates from three brain slices for 4 h and two brain slices for all other time points). Figure 6j: Pearson chi-square tests showed no statistical difference in the results of the same condition between the two imaging methods, α = 0.05. *P* values for each chi-square test (Magnify–SOFI versus EM): Normal: 0.87; PAX: 0.38, PCD: 0.50. *P* values for each interclass chi-square test (Magnify): Normal versus PAX: 0.0007; Normal versus PCD: 2.2 × 10^−16^; PAX versus PCD: 4.9 × 10^−9^. *P* values for each interclass chi-square test (EM): Normal versus PAX: 2.2 × 10^−5^; Normal versus PCD: «1 × 10^−16^; PAX versus PCD: 2.2 × 10^−13^. On the *x* axis, *n* is the number of cilia measured from three different organoids. For statistical significance, ***P < 0.007. Scale bars are all in biological scales.

We applied a simple criterion to define cilia defects: to count whether the outer microtubules are nine pairs, and whether the central microtubules are two. If both are true, the cilium are considered normal, otherwise defective. Statistical Pearson chi-square tests were performed to compare Magnify–SOFI images and electron micrographs of cilia with and without defects as well as compare interclass variations. A *P* value < 0.05 was considered statistically significant.

### Animal use ethical statement

All experimental procedures involving animals were conducted in accordance with the National Institutes of Health (NIH) guidelines and were approved by the Institutional Animal Care and Use Committee at Carnegie Mellon University and by Brown University Institutional Animal Care and Use Committee.

## Supplementary Material

Supplemental Video 1 3D rendering of a fully expanded Magnify-processed human FFPE kidney tissue stained with DAPI (magenta), ACTN4 (orange) and WGA (blue) taken at ×40 magnification.

Supplemental Video 2 3D rendering of a fully expanded Magnify-processed human FFPE colon tissue stained with DAPI (magenta), ATPIF (green) and Cytokeratin Pan Type I/II (blue) taken at ×40 magnification.

Supplemental Video 3 3D rendering of a fully expanded Magnify-processed human FFPE placenta tissue stained with DAPI (magenta), ATPIF (green) and Cytokeratin Pan Type I/II (blue) taken at ×40 magnification.

Supplemental Video 4 3D rendering of a fully expanded Magnify-processed human FFPE breast tissue stained with DAPI (magenta), ATPIF (green) and Cytokeratin Pan Type I/II (blue) taken at ×40 magnification.

Supplemental Video 5 3D rendering of an expanded human urinary bladder cancer tissue section stained with DAPI (white), DNA FISH probe against centromere binding protein B box motif CEPN-B (cyan), DNA FISH probe against telomere motif TelC and WGA (yellow) taken at ×40 magnification.

Supplemental Video 6 3D rendering of an SST neuron in Magnify-processed mouse brain stained with DAPI (white), anti-GFP (blue), synaptophysin (magenta) and PSD95 (green) expanded in 1x PBS and taken at ×40 magnification.

Supplementary Material

Supplemental Video 7 3D rendering of SST dendrites in Magnify-processed mouse brain stained with DAPI (white), anti-GFP (blue), synaptophysin (magenta) and PSD95 (green) expanded in 1x PBS and taken at ×40 magnification.

Supplemental Video 8 3D rendering of SST dendrites in Magnify-processed mouse brain stained with DAPI (white), anti-GFP (blue), synaptophysin (magenta) and PSD95 (green) expanded in 1x PBS and taken at ×40 magnification.

Supplemental Video 9 3D rendering of a Magnify–SOFI image stack of fully expanded ependymal cilia and basal bodies from the ependymal cell lining in the adult mouse brain stained with NHS-ATTO-488 taken at ×40 magnification.

## Data Availability

Data used to generate figures can be found at https://github.com/zhao-biophotonics/. All other data are available upon reasonable request to the corresponding author of the paper.
