# Supplementary material for: Magnify is a universal molecular anchoring strategy for expansion microscopy
Source: Nat Biotechnol. Author manuscript; Available in PMC 2023 Jul 1. (PMC10264239; doi:10.1038/s41587-022-01546-1)
Supplement: Supplementary Material [file NIHMS1860505-supplement-Supplementary_Material.docx]

**Magnify: A versatile 11-fold expansion microscopy technique with universal molecular retention**

**Authors**

Aleksandra Klimas^1±^, Brendan R. Gallagher^1±^, Piyumi Wijesekara^2^, Sinda Fekir^3,4^, Emma F. DiBernardo^1^, Zhangyu Cheng^1^, Donna B. Stolz^5^, Franca Cambi^6,7^, Simon Watkins^5^, Steven L Brody^8^, Amjad Horani^9,10^, Alison L. Barth^1^, Christopher I. Moore^3,4^, Xi Ren^2^, Yongxin Zhao^1+^

**Affiliations**

^1^Department of Biological Sciences, Carnegie Mellon University, Pittsburgh, Pennsylvania, USA. ^2^Department of Biomedical Engineering, Carnegie Mellon University, Pittsburgh, Pennsylvania, USA. ^3^Department of Neuroscience, Brown University, Providence, Rhode Island, USA. ^4^Carney Institute for Brain Science, Brown University, Providence, Rhode Island, USA. ^5^Department of Cell Biology, Center for Biologic Imaging, University of Pittsburgh, Pittsburgh, Pennsylvania. ^6^Veterans Administration Pittsburgh, Pittsburgh, Pennsylvania. ^7^Department of Neurology/PIND, University of Pittsburgh, Pittsburgh, Pennsylvania, USA. ^8^Department of Medicine, Washington University School of Medicine, St. Louis, Missouri, United States. ^9^Department of Pediatrics, Washington University School of Medicine, St. Louis, Missouri, United States. ^10^Department of Cell Biology and Physiology, Washington University School of Medicine, St. Louis, Missouri, United States

±These authors contributed equally to this work

Aleksandra Klimas, Brendan R. Gallagher

^+^Correspondence to [yongxinz@andrew.cmu.edu](mailto:yongxinz@andrew.cmu.edu)

Contents

[Supplementary Notes 3](#_Toc115345688)

[Supplementary Note 1: Exploration of Anchoring and Homogenization Buffers 3](#_Toc115345689)

[Supplementary Note 2: Exploration of Gel Chemistry 4](#_Toc115345690)

[Supplementary Note 3: DNA FISH experiment on tissues homogenized in MAGNIFY gel 5](#_Toc115345691)

[Supplementary Note 4: Use of SOFI with MAGNIFY 5](#_Toc115345692)

[Supplementary Note 5: Parameter free resolution estimation of MAGNIFY-SOFI 5](#_Toc115345693)

[Supplementary Note 6: High-quality imaging of tubulin ultrastructure in cell culture. 6](#_Toc115345694)

[Supplementary Figures 8](#_Toc115345695)

[Supplementary Figure 1: The full workflow for MAGNIFY. 8](#_Toc115345696)

[Supplementary Figure 2: Comparison of anchoring, homogenization, and gelling strategies. 10](#_Toc115345697)

[Supplementary Figure 3: Examples of Post Expansion Staining with MAGNIFY 11](#_Toc115345698)

[Supplementary Figure 4: Exploration and characterization of MAGNIFY gel chemistry. 13](#_Toc115345699)

[Supplementary Figure 5: Overview of SOFI 16](#_Toc115345700)

[Supplementary Figure 6: Example images of tubulin structure in cell culture and cilia in human lung organoids. 17](#_Toc115345701)

[Supplementary Figure 7: Example images of MAGNIFY expansion of FFPE tissue. 19](#_Toc115345702)

[Supplementary Figure 8: DNA FISH with MAGNIFY using FFPE human tissue. 20](#_Toc115345703)

[Supplementary Videos 22](#_Toc115345704)

[Supplementary Video 1. 22](#_Toc115345705)

[Supplementary Video 2. 22](#_Toc115345706)

[Supplementary Video 3. 22](#_Toc115345707)

[Supplementary Video 4. 22](#_Toc115345708)

[Supplementary Video 5. 22](#_Toc115345709)

[Supplementary Video 6. 22](#_Toc115345710)

[Supplementary Video 7. 22](#_Toc115345711)

[Supplementary Video 8. 22](#_Toc115345712)

[Supplementary Video 9. 22](#_Toc115345713)

[Supplementary Tables 23](#_Toc115345714)

[Supplementary Table 1: Condition optimization for different tissue types 23](#_Toc115345715)

[Supplementary Table 2: Protein Retention for tissues under different anchoring and homogenization conditions. 24](#_Toc115345716)

[Supplementary Table 3: Lipid retention in mouse brain under different anchoring and homogenization conditions. 25](#_Toc115345717)

[Supplementary Table 4: Validated Primary Antibodies and fluorescent labels for Pre and Post MAGNIFY Staining 25](#_Toc115345718)

[Supplementary Table 5: Secondary antibodies used for pre- and post-MAGNIFY staining. 26](#_Toc115345719)

[Supplementary Table 6: Comparison of expansion factors for different hydrogel chemistries for different tissue types. 27](#_Toc115345720)

[Supplementary Table 7: Exploration of different hydrogel chemistries and their respective expansion factors of blank gels in water. 27](#_Toc115345721)

[Supplementary Table 8: Gelling Conditions for MAGNIFY Gel. 28](#_Toc115345722)

[Supplementary Table 9: Deformation testing of different gel chemistries 28](#_Toc115345723)

[Supplementary Table 10: Gelling Solution Recipe 29](#_Toc115345724)

[Supplementary Table 11: List of Chemicals and Reagents 30](#_Toc115345725)

[Supplementary Table 12: DNA FISH probes design 31](#_Toc115345726)

[References 44](#_Toc115345727)

## Supplementary Notes

### Supplementary Note 1: Exploration of Anchoring and Homogenization Buffers

We gelled deparaffinized and antibody labeled FFPE kidney pieces with the MANGIFY monomer solution containing 0.05% (v/v) methacrolein (**Supplementary Fig. 2a**) allowing the samples to still be homogenized by proteinase K (ProK) digestion, which is typically used to ensure isotropic expansion in most ExM protocols.^1,2^ To quantify protein retention (**Fig. 1e,f**; **Supplementary Fig. 2f**; **Supplementary Table 2**), we compared average fluorescence of N-Hydroxysuccinimide (NHS) ester-dye conjugates, which bind to primary amines, before and after processing with the MAGNIFY protocol. To compare to previous protocols, FFPE kidney samples were prepared following the modified expansion pathology (ExPath) protocol.^1,3^ With this protocol, the samples were gelled in a monomer solution containing 15% (w/v) SA, 5% (w/v) AA, 0.1% (w/v) Bis, and 11.7% (w/v) NaCl along with 0.01% (w/v) 4HT, 0.2% (v/v) TEMED, and 0.2% (w/v) APS after overnight incubation with 0.05 mg/mL AcX. After homogenization with ProK digestion buffer (50 mM Tris (pH 8), 25 mM EDTA, 0.5% w/v TritonX, 0.8M NaCl, 2 units/mL ProK) for 3 hours at 60ºC, only 3.08 ± 0.46% s.e.m. (*n* = 9 technical replicates) of pre-expansion NHS fluorescence was observed (**Fig. 1e**). Meanwhile samples anchored with 0.05% (v/v) methacrolein using the MAGNIFY gel chemistry measured 14.54 ± 1.07% s.e.m. (n = 12 technical replicates) of pre-expansion NHS fluorescence (**Fig. 1e**) when homogenized under the same conditions. Similarly, for PFA fixed mouse brain tissue anchored with 0.1% (v/v) methacrolein or 0.05 mg/mL AcX using the MAGNIFY gel chemistry measured 6.19 ± 0.34% s.e.m. (*n* = 20 technical replicates) and 7.7% ± 0.41% s.e.m. (*n* = 20 technical replicates), respectively, retained fluorescence after 2 hours homogenization with ProK (**Fig. 1e**; **Supplementary Table 2**). Additionally, lipid retention between the two conditions was comparable for PFA fixed mouse brain tissue (**Supplementary Fig. 2f**; **Supplementary Table 3**), where lipid retention measured by retained fluorescence of DiD was 18.13 ± 0.44% s.e.m. (*n* = 20 technical replicates) and 17.06 ± 0.8% s.e.m. (*n* = 20 technical replicates) for 0.1% (v/v) methacrolein anchoring and AcX anchoring, respectively, after 2 hours homogenization with ProK. Lipid retention measurements are a comparison of the same lipophilic dye before and after expansion, and it is a measure of how much of the initial stain is still present after expansion. Therefore, if a particular structure is not stained by the lipophilic dye before expansion (such as the cell membrane) due to fixation, permeabilization, or other reasons, it cannot be revealed by MAGNIFY (**Supplementary Fig. 2e**).

To better preserve protein epitopes during expansion, we sought to replace the strong protease digestion of ProK. Protocols such as magnified analysis of the proteome (MAP)^4^ utilize a non-enzymatic surfactant based tissue denaturing solution, but we found this to be incompatible with both AcX and methacrolein based anchoring (**Supplementary Fig. 2b**). Pulling from the ExPath^1^ protocol and clearing protocols such as CUBIC,^5^ we found that the addition of EDTA and urea, respectively, to a non-ionic surfactant based solution could adequately homogenize tissues and preserve biomolecules (**Supplementary Figure 2c**) while providing isotropic expansion. Indeed, both EDTA and urea were necessary to adequately homogenize samples anchored with methacrolein (**Supplementary Figure 2d**). Both AcX and methacrolein anchored FFPE kidney samples retained fluorescence after homogenizing using a non-ionic surfactant-based buffer (10% (w/v) SDS, 8M urea, 25 mM EDTA, 2x PBS pH 7.5 at RT) for 60 hours at 80 ºC (**Fig. 1e**; **Supplementary Fig. 2f**). AcX anchored samples retained 2.94 ± 0.57% s.e.m. (*n* = 14 technical replicates) NHS fluorescence which was comparable to the ProK homogenized samples. Meanwhile, samples anchored with 0.05% (v/v) methacrolein retained 13.63 ± 1.86% s.e.m. (*n* = 8 technical replicates) under the same conditions. Similarly, PFA fixed mouse brain tissue retained48.44 ± 1.49% s.e.m. (n = 20 technical replicates) and 12.82 ± 0.77% s.e.m. (n = 20 technical replicates) NHS fluorescence for 0.1% methacrolein and AcX anchored samples, respectively, when homogenized for 4 and 8 hours, respectively, at 80ºC (**Fig. 1e; Supplementary Table 2**). Under the same conditions, MAGNIFY showed improved lipid retention, where 0.1% (v/v) methacrolein anchored samples retained 98.10 ± 2.36% s.e.m. (*n* = 30 technical replicates) DiD fluorescence and AcX anchored samples retained 74.50 ± 2.47% s.e.m. (*n* = 20 technical replicates) after homogenizing for 4 and 8 hours, respectively in hot surfactant (**Fig. 3a**; **Supplementary Fig. 2f; Supplementary Table 3**).

To improve biomolecule retention, we found that increasing the methacrolein to 0.25% (v/v) for FFPE kidney samples preserved 58.54% ±7.08% s.e.m. (*n* = 14 technical replicates) of the NHS signal after homogenization for 60 hours at 80 ºC using a surfactant-based buffer (**Supplementary Fig. 2f**). Similar to methacrolein concentration, homogenization time was tissue-type dependent (**Supplementary Table 1**), where mechanically tougher and more heavily fixed tissues such as FFPE kidney took longer to homogenize compared to softer tissue such as PFA fixed mouse brain. We also found the expansion factor was dependent on methacrolein concentration, where both over and under anchoring led to smaller expansion factors (**Supplementary Fig. 4**) and the optimal methacrolein concentration was tissue-type dependent (**Supplementary Table 1**).

### Supplementary Note 2: Exploration of Gel Chemistry

To optimize gel chemistry, we first attempted to replicate the X10 protocol,^6^ where N,N- dimethylacrylamide acid (DMAA) was used to obtain larger expansion factors. We first made a gelling solution of 26.7% (w/v) DMAA and 6.4% (w/v) SA was prepared in water. After dissolving, a 3.6% (w/v) stock solution of KPS was added to a final concentration 0.36% (w/v) KPS. After vortexing, TEMED (100% w/w) was added to a final concentration of 0.4% (v/v). To replicate ExPath gelling conditions, we chose to omit the nitrogen purging step. When applied to FFPE kidney pieces treated with AcX prior to gelling, incomplete expansion and non-isotropic expansion was observed in samples homogenized in a non-ionic surfactant-based buffer (10% (w/v) SDS, 8M urea, 25 mM EDTA, 2x PBS pH 7.5 at RT) for 60 hours at 80 ºC (**Supplementary Fig. 2c**). Additionally, these tissue gels were not mechanically sturdy and only had an expansion factor of ~6X when expanded in water (**Supplementary Table 6**; **Supplementary Fig. 4**).

To provide better expansion in a sturdy gel, we chose to supplement the ExPath gelling solution with a small amount of DMAA. To find the optimal gel composition, we varied DMAA, (SA, N,N′-Methylenebisacrylamide (Bis), and acrylamide (AA) concentrations and prepared blank gels and compared their expansion in water. From the rapid ExPath protocol, we knew increasing SA and AA concentrations provided a sturdier gel to compensate for the lower Bis concentration, which increased the expansion factor.^3^ We ultimately found a hydrogel formula composed of 4% (w/v) DMAA, 34% (w/v) SA, 10% (w/v) AA, and 0.01% (w/v) Bis (**Supplementary Table 7**) provided the optimal expansion factor and sturdiness to expand an array of tissue types. We also varied the initiator, ammonium persulfate (APS), and inhibitor, 4-hydroxy-TEMPO (4HT), concentrations and polymerized the blank gels at different temperatures and found that a slightly higher APS concentration and slightly lower 4HT concentration compared to the ExPath protocol in gels polymerized at 37 ºC provided the best expansion factor (**Supplementary Table 8**). It should also be noted the blank gel expansion factors are slightly smaller than that of the tissue-gel hydrogel due to different components contributed to the hydrogel by the tissue.

To test the mechanical sturdiness of the gel, we compared the MAGNIFY gel chemistry to other gels using the method developed for the Ten-fold Robust Expansion Microscopy (TREx) protocol (**Supplementary Fig. 4**, **Supplementary Table 9**).^7^ The TREx gel is comprised of 11% (w/v) SA, 14.5% (w/v) AA, 0.005-0.015% (w/v) Bis, and 1x PBS along with 0.15% (v/v) TEMED, and 0.15% (w/v) APS. Briefly, blank gels were made in a 2 mL Eppendorf tube and then cut into half cylinders. The gels were then expanded in water and the deformation index was calculated by taking the ratio of radius of deformation to the radius of the gel. For the TREx protocol, higher crosslinker concentrations (150 ppm Bis) compared to the reported values (50 and 90 ppm Bis) were needed to produce a deformation index less than 0.5. Although both the high crosslinker TREx gel and MAGNIFY had similar expansion factors (5.9× in water), MAGNIFY had a much lower deformation index of 0.03 compared to the 0.28 deformation index of the high crosslinker TREx gel.

Interestingly, the crosslinking provided by the DMAA also had an anchoring effect; FFPE kidney samples anchored with 0% (v/v) methacrolein (**Supplementary Fig. 2c**) retained 2.43 ± 0.83% s.e.m. (n = 8 technical replicates) and 4.40 ± 0.64% s.e.m. when homogenized for 60 hours at 80 ºC using a surfactant-based buffer and with ProK homogenization using this gel chemistry.

### Supplementary Note 3: DNA FISH experiment on tissues homogenized in MAGNIFY gel

We applied DNA FISH probes against telomere sequences^8^ and centromere protein B box^9^ to gel-embedded bladder cancer samples homogenized with strong ProK digestion, as ProK will homogenize proteins within tissue without damaging nucleic acids. We observed that these two probes diffused into MAGNIFY processed bladder cancer specimens and hybridized with chromosomal DNA within 2 hours at 37 °C and post-expansion lectin staining could still be performed (**Fig. 3m, Supplementary Video 5**). Using strong ProK digestion, we noticed that FFPE bladder cancer tissue section can be processed and stained with DNA FISH probes and lectin stain within 8 hours, which could be useful in time-sensitive applications such as those used for histopathological diagnoses.

### Supplementary Note 4: Use of SOFI with MAGNIFY

We chose to pair MAGNIFY with SOFI as SOFI can work on standard fluorophores^10^ images can be generated with under 100 time points^10^, and it has been shown to work on 3D datasets^10,11^. SOFI was performed on expanded samples with custom MATLAB code, where images were corrected for drift, intensity and deconvolved (Lucy-Richardson method). The SOFI processing used in MAGNIFY-SOFI is based on CryoSOFI code^12^, but only second-order cross-correlation was performed and the code was modified to function in 3D (Supplemental Fig. 6). We noted that the axial resolution improvement is limited as Z-planes were not captured simultaneously^11^.

### Supplementary Note 5: Parameter free resolution estimation of MAGNIFY-SOFI

To determine the effective resolution achieved by MAGNIFY and MAGNIFY-SOFI, we applied a parameter-free algorithm based on decorrelation analysis to images of human lung organoid (Apical out airway organoids^13^, both normal and with CCDC39 gene mutations), expanded 10.5×. Organoids were stained with NHS-Cy3 and imaged using a CFI Plan Apochromat VC 60×C water immersion (1.2 NA), with a measured resolution of 36.52 ± 0.95 nm (**Supplementary Fig. 5d**). Lucy-Richardson deconvolution was then performed on the dataset, improving the measured resolution to 23.54 ± 1.31 nm. Finally, a resolution of 15.90 ± 1.39 nm was demonstrated on deconvolved MAGNIFY-SOFI images. Values are based on 37 measurements in each condition across 4 technical replicates. Depending on the wavelength of fluorophore, assuming the expansion factor is consistent with this organoid example, the effective resolution of MAGNIFY-SOFI is estimated to be 13~18 nm.

### Supplementary Note 6: High-quality imaging of tubulin ultrastructure in cell culture.

U2OS cells (Gifted from the Lee lab at Carnegie Mellon University, originally purchased from ATCC) were grown in DMEM supplemented with 10% fetal bovine serum, 4.5 g/L D-glucose, 110 mg/L sodium pyruvate, 6 mM L-glutamine, 0.1 mM non-essential amino acids, 50 units/mL of penicillin and 50 µg/mL of streptomycin. Cells were not authenticated or tested for mycoplasma contamination. All cells were maintained at 37°C in a humid 5% CO_2_ atmosphere. Cells were seeded unto #1.5 cover glass treated with poly-l-lysine in a 6 well plate and grown for 24-48 hours before fixation.

There is no standardized fixation protocol for super-resolution microtubule imaging, and thus this protocol requires optimization for new applications. The optimized protocol for high-quality tubulin images with MAGNIFY is based off existing protocols^14–17^ and is as follows: 1 hour before fixation, media was replaced with DMEM containing 0% FBS and the cells were returned to the 37°C incubator. After the 1 hour, we brought the well plate out to a RT bench top, aspirated the media, and quickly extracted the coverslips (< 30 s) with freshly made 37°C PEMT buffer (80 mM PIPES, 5 mM EGTA, 2 mM MgCl_2_ at pH 6.8 with 0.2% TX). Quickly after we aspirated the PEMT buffer, we applied 2 ml of cold -20°C 100% methanol to each well, and then fixed the plate at -20°C for 5 minutes. We then replaced the methanol with RT 1x PBS and performed consecutive washes with PBS for 30 s, 1 min, 5 min, 10 min, and 15 min, using the 2-handed pipette technique to reduce mechanical stress and further dehydration.

MAGNIFY gel monomer solution was prepared as described in the methods section. Prior to gelation, methanol fixed U2OS cell samples were placed into custom gelling chamber consisting of four spacers cut from #1.5 cover glass adhered to the uncoated back of a microscope slide on either side of a piece of cover glass with adherent cells. Excess PBS around the specimen was absorbed with a Kimwipe and the specimen was allowed to air dry partially. Immediately prior to gelation, the chemicals APS, TEMED, and methacrolein were added to a final concentration of 0.25% (w/v) APS, 0.2% TEMED (w/v), and 0.1% (v/v) methacrolein. The solution was vortexed and applied to the U2OS cells. A glass slide was then placed on top of the double-stacked spacers to form a gelling chamber. The samples were incubated overnight in a humidified container at 37 ºC to complete gelation.

After gelation, the glass slide cover was removed from the gelling chamber, blank gel surrounding the tissue was trimmed from the samples, and the U2OS cell containing hydrogel was cut into smaller pieces. Samples were then incubated in homogenization buffer (10% w/v SDS, 8M Urea, 25 mM EDTA, 2× PBS, pH 7.5 at RT) for 36h at 80 ºC with shaking. Homogenized samples were then washed 3 times with 1× PBS at RT, followed by at least 3 washes in 1% decaethylene glycol monododecyl ether (C_12_E_10_)/1xPBS or 1%PBST at RT or 60 ºC to remove remaining SDS.

After homogenization and washing, U2OS cells were stained with approximately 1 µg/mL of a combination of rabbit anti-αTubulin (Abcam ab18251, Proteintech 80762-1-RR, 11224-1-AP), rabbit anti-βTubulin (Proteintech 10094-1-AP), mouse anti-αTubulin (Invitrogen 62204, Proteintech 66031-1-Ig), and mouse anti-βTubulin (Proteintech 66240-1-Ig, Sigma T8328) in staining buffer (5× SSC (750 mM NaCl, 75 mM sodium citrate, pH 7.0)/0.1% Tween 20) for 48h RT. Samples were then washed 3 times with washing buffer (1×PBS/0.1% (C_12_E_10_)/1xPBS) at RT for at least 10 minutes. To prevent disassociation during full expansion in water, samples were stained with both fluorescently conjugated secondaries and biotin-streptavidin labeling. First, samples were incubated in staining buffer with AF488 AffiniPure Fab Fragment Donkey Anti-Rabbit (Jackson ImmunoResearch 711-547-003) , AF488 AffiniPure Fab Fragment Donkey Anti-Mouse (715-547-003), Biotin-SP AffiniPure Fab Fragment Goat Anti-Rabbit (111-067-003), and Biotin-SP AffiniPure Fab Fragment Goat Anti-Mouse (115-067-003) diluted to approximately 1 µg/mL in staining buffer for at least 1 hour at RT. Samples were then washed at least 3 times with washing buffer for at least 10 minutes. Samples were then incubated with AF488 conjugated streptavidin (Invitrogen S11223) diluted to approximately 1 µg/mL in staining buffer for at least 20 minutes at RT. Samples were then washed at least 3 times with washing buffer for at least 10 minutes. After staining, samples were washed in water for at least 10 minutes. This was repeated until the sample was fully expanded, at least three exchanges of water.

## Supplementary Figures

**
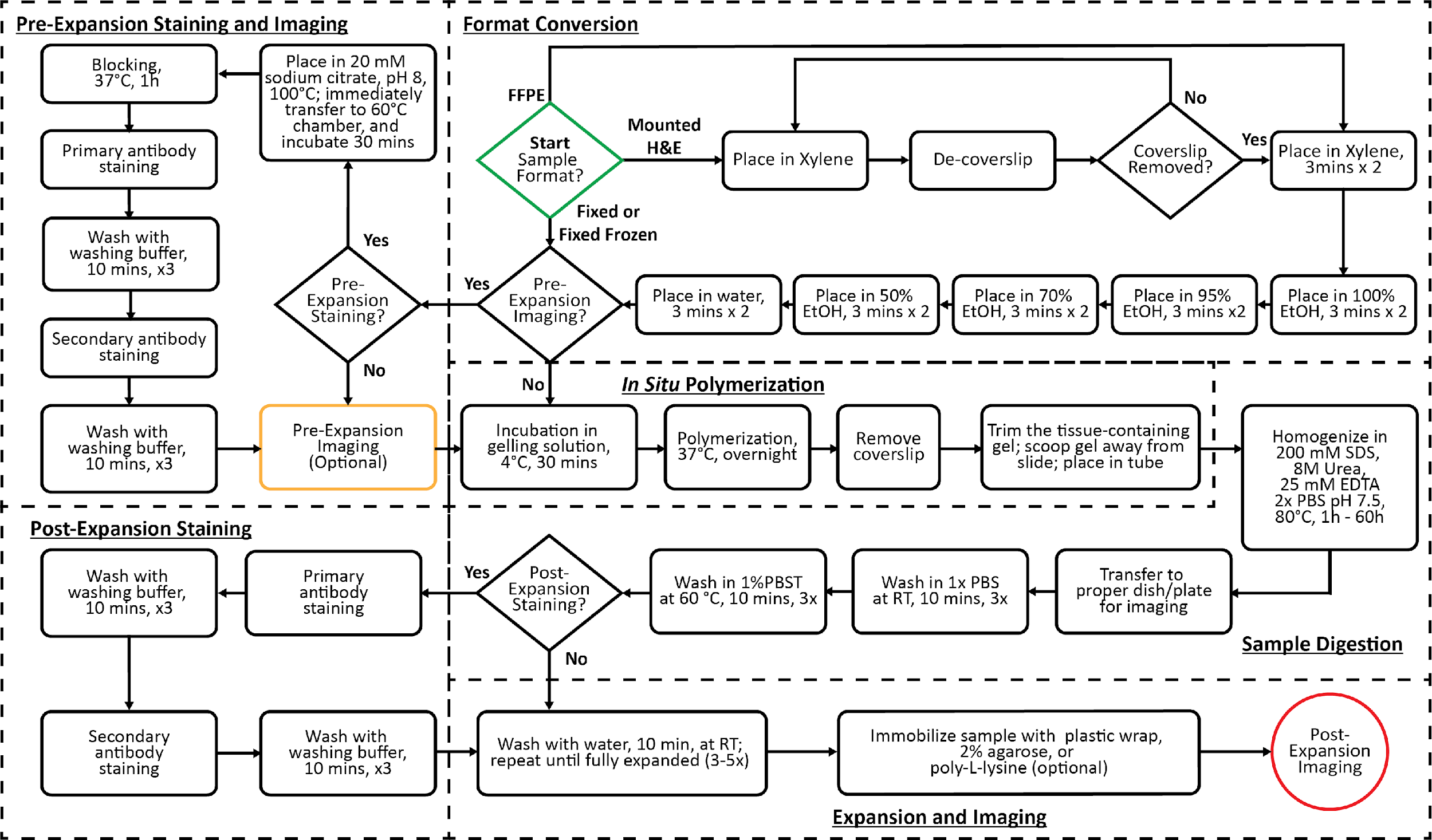
**

### **Supplementary Figure 1:** The full workflow for MAGNIFY.


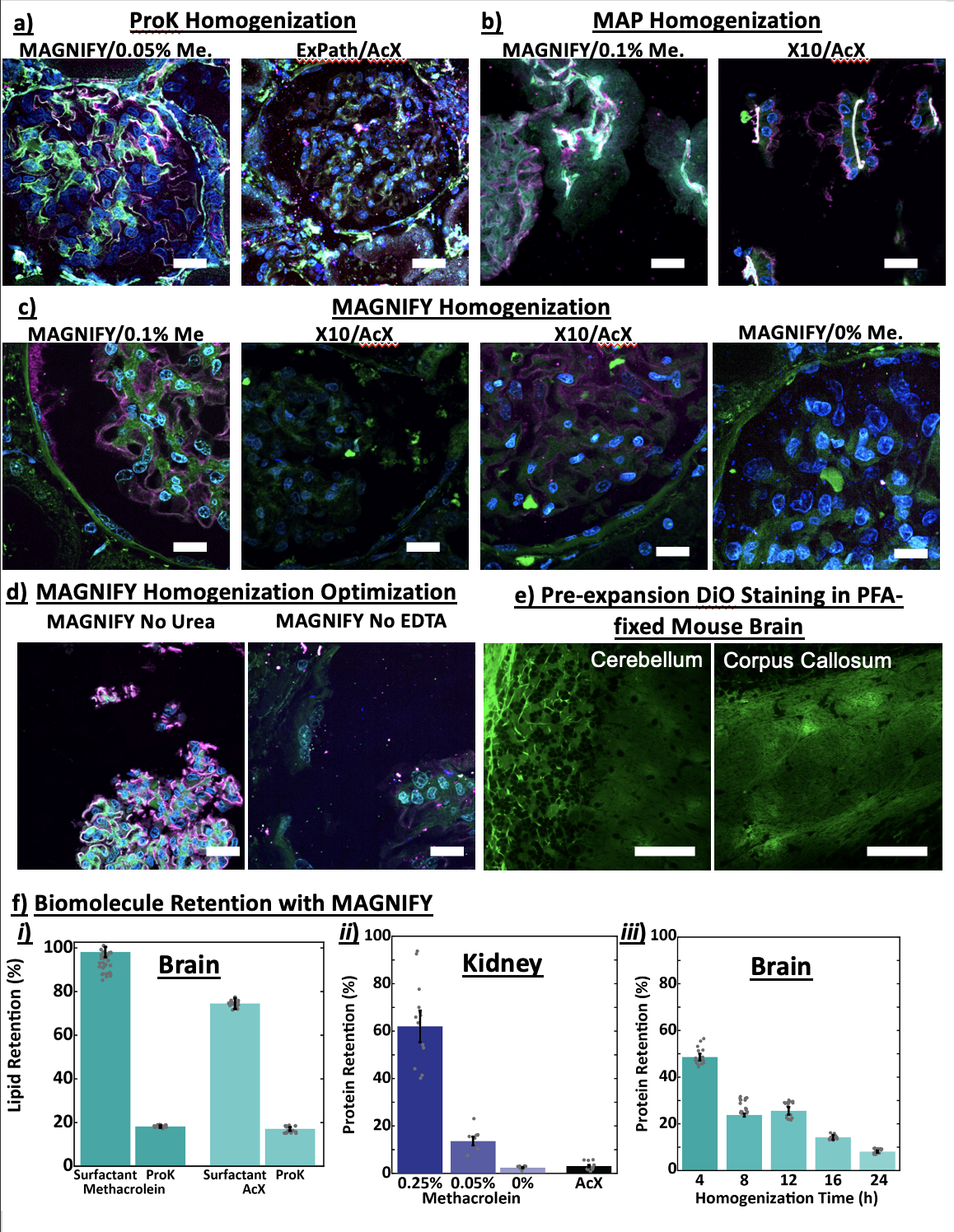


Supplementary Figure 2: Comparison of anchoring, homogenization, and gelling strategies. **(a)** Deparaffinized and stained FFPE kidney samples were gelled according to the MAGNIFY or ExPath protocols and were incubated in the ExPath homogenization buffer (50 mM Tris pH 8, 25 mM EDTA, 0.5% w/v TritonX, 0.8M NaCl) with Proteinase K diluted by 1:200 (final concentration 4 units/mL) for 3 hours at 60 °C. Final expansion factors: 4.2 (MAGNIFY) and 3.3 (ExPath). **(b)** Kidney samples prepared with the MAGNIFY protocol anchored with 0.1% (v/v) methacrolein, X10 protocol anchored with AcX, and the MAGNIFY protocol showed severe cracking due to incomplete homogenization when expanded in water after homogenizing for 60h at 80°C in the MAP homogenization buffer (200 mM SDS, 200 mM NaCl, and 50 mM Tris in water pH 9 at RT). **(c)** Samples prepared with the MAGINFY protocol anchored with 0.1% (v/v) methacrolein, ExPath protocol anchored with AcX, and the x10 protocol anchored with AcX could be expanded in water after homogenization using a non-ionic surfactant buffer (10% w/v SDS, 8M Urea, 25 mM EDTA, 2x PBS, pH 7.5 at RT) for 60h at 80°C. However, the x10 prepared samples had less even homogenization and smaller expansion compared to the MAGNIFY sample. Some anchoring is present in samples without an anchoring agent, most likely due to the crosslinking capability of DMAA. Optimal anchoring was observed in 0.25% (v/v) methacrolein anchored kidney samples. Expansion factors: 7.9 (MAGNIFY/0.1%Me.) and 6.0 (X10). **(d)** When urea or EDTA were removed from the non-ionic surfactant buffer, insufficient homogenization led to incomplete expansion and severe cracking in FFPE kidney samples homogenized for 60h at 80°C. Gels were expanded in water and representative maximum intensity projection images were obtained at 10x magnification. Scale bar 100 µm (absolute scale) Blue DAPI, green NHS, magenta WGA. **(e)** Pre-expansion DiO staining in the cerebellum (left) and corpus callosum (right) in PFA-fixed mouse brain tissue showing strong myelin staining but no strong staining of the neuronal plasma membrane. Scale bar: 50 µm. **(f)** Protein and Lipid retention for mouse brain and human kidney. ***(i)*** Lipid retention measured as DiD fluorescence retention for mouse brain homogenized in surfactant versus ProK for tissues anchored with 0.1% methacrolein or AcX. N = 20 technical replicates. Error bars in terms of s.e.m. ***(ii)*** Protein retention of human kidney measured as NHS fluorescence retention processed with MAGNIFY versus ExPath and anchored with different conditions and homogenized in hot surfactant for 60 hours. N = 13, 8, 8, and 9 technical replicates***. (iii)*** Protein retention of mouse brain measured as NHS fluorescence retention processed with MAGNIFY and anchored with 0.1% methacrolein and homogenized in hot surfactant over time. N = 20 technical replicates.


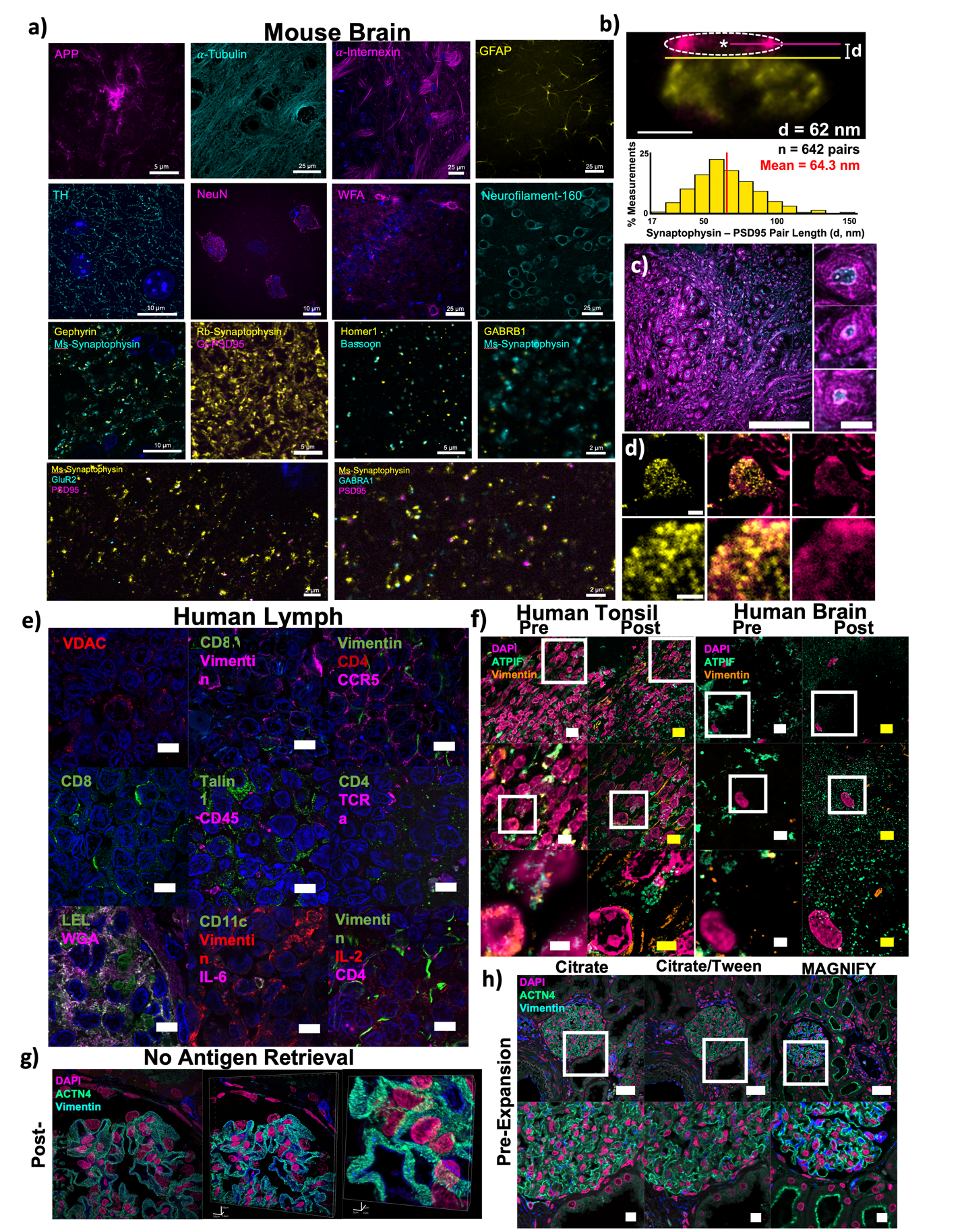


Supplementary Figure 3: Examples of Post Expansion Staining with MAGNIFY **(a)** Paraformaldehyde-fixed 30µm mouse brain sections were gelled according to the MAGNIFY protocol using 0.1%-1% (v/v) methacrolein and were homogenized using a non-ionic surfactant buffer (10% w/v SDS, 8M Urea, 5-25 mM EDTA, 1-2x PBS, pH 7.5 at RT) for 4-8h at 80°C. After expansion, samples were stained with indicated primary antibodies in 1x PBS overnight. After washing, samples were stained with DAPI and relevant secondary antibodies. After staining, samples were washed in 1x PBS and imaged at 10x (APP, GFAP, WFA, Neurofilament-160) or 40x (𝛼-Tubulin, 𝛼-Internexin, TH, NeuN, all synaptic markers) magnification. Blue, DAPI. **(b)** Quantification of synaptophysin-PSD95 pair distances in mouse striatum expanded fully with MAGNIFY. Top: Example measurement. The distance, *d*, was taken from the edge of synaptophysin (yellow) to the center of the postsynaptic density (PSD95, magenta). Bottom: Summary of all measurements taken. Y-axis represents the number of measurements as a percentage of all measurements taken. n = 642 measurements across two technical replicates. Mean pair distance = 64.3 nm. Expansion factor: 11*×* in ddH_2_O. **(c)** Visualization of myelinated axons in the mouse brain with MAGNIFY. Cyan: NHS-Cy3, Magenta: DiD. Scale bars: Overview: 10 µm, Zoom-ins: 1 µm. **(d)** Lipophilic dye specificity for synaptic vesicles in the mouse brain. Left: synaptophysin, yellow. Right: DiD, magenta. Middle, merge. Bottom Row: Zoom-in of top row. Areas of high vesicle density (as shown by synaptophysin labeling) can be seen to be labeled with DiD as well. Scale bar top: 200nm; bottom: 100 nm. **(e)** Deparaffinized human lymph node samples purchased from US Biomax were gelled according to the MAGNIFY protocol using 0.1% (v/v) methacrolein and were homogenized using a non-ionic surfactant buffer (10% w/v SDS, 8M Urea, 5-25 mM EDTA, 1-2x PBS, pH 7.5 at RT) for 26-32h at 80°C. After expansion, samples were stained with indicated primary antibodies in staining buffer overnight. After washing, samples were stained with DAPI and relevant secondary antibodies. After staining, samples were washed in 1xPBS and imaged at 40x magnification. Scale bar 50 um (absolute scale) Blue DAPI. **(f)** MAGNIFY improves post expansion staining. Human tonsil (left) and brain (right) FFPE samples were imaged at 40x (top left, scale bar 10 μm, middle left scale bar 5μm, bottom left scale bar 2 μm). The white box indicates the field of view of the higher magnification images. The samples were then processed with the MAGNIFY protocol, and the same fields of view were imaged post-expansion in water at 10× (top right, scale bar, 10 μm biological scale) and 40x (middle right scale bar 5μm, bottom right scale bar 2 μm; biological scale). Post expansion images were projected over 4-17 z slices. Expansion factors in water were: 8.04× (tonsil), 8.36× (brain). Staining of ATIPF and vimentin improve with post expansion staining. Magenta, DAPI; Green, ATPIF; Orange, Vimentin. **(g)** MAGNIFY does not require antigen retrieval. MAGNIFY protocol achieves good post expansion staining without the need for antigen retrieval pre-expansion. Scale bar 25 μm (absolute scale). Foot processes in kidney podocytes can still be resolved. Magenta, DAPI; Green, ACTN4, Blue, Vimentin. **(h)** The MAGNIFY homogenization buffer can be used as a superior pre-expansion antigen retrieval buffer**.** After heat treatment for 30-45 minutes in 200 mM sodium citrate (pH 8), 200 mM sodium citrate, 0.05% tween (pH 6.5), or 10% w/v SDS, 8M Urea, 25 mM EDTA, 2x PBS (pH 9.5), the MAGNIFY buffer demonstrates superior pre-expansion staining. Scale bar top: 50 μm; bottom: 10 μm.


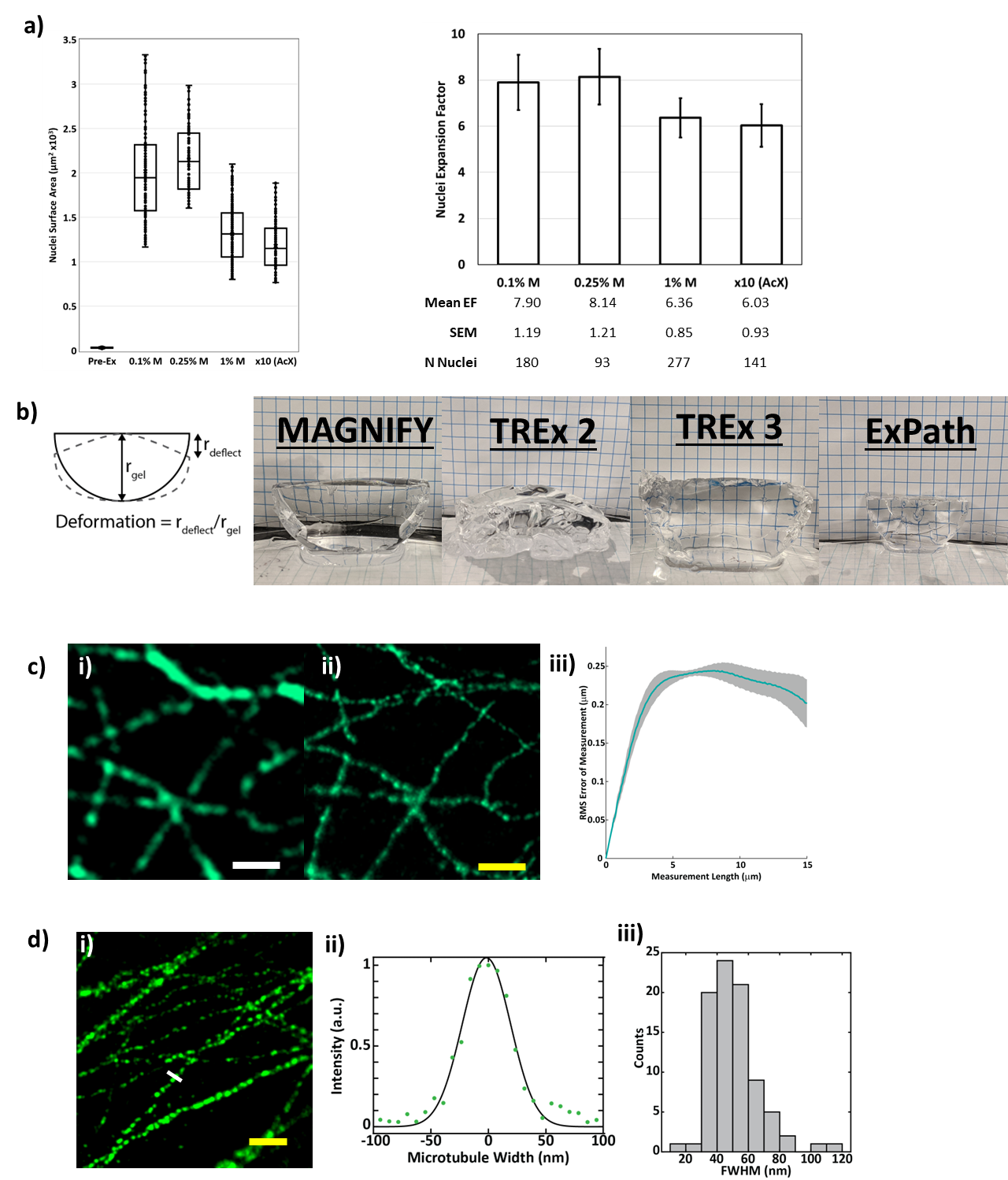


Supplementary Figure 4: Exploration and characterization of MAGNIFY gel chemistry. **(a)** Estimation of Expansion Factors for Gelling and Anchoring Strategies. Kidney samples prepared with the x10 protocol or MAGNIFY with different methacrolein concentrations. All samples were homogenized for 60 hours at 80 °C. To estimate expansion factors, average nuclear areas were measured in ImageJ (left). The box defines the interquartile range, divided by the median. The whiskers delineate the minimum and maximum values for each data set. Each anchoring condition was then divided by the average nuclear size from the pre-expansion images and then averaged to calculate expansion factors for the different anchoring strategies (right) with mean expansion factors, sdandard error of the mean (SEM) values, and n nuclei measured listed in the table below. Error bars are given in terms of SEM. **(b)** Measurement of Deformation of different gel chemistries. Blank gels were prepared in 2 mL Eppendorf tubes and gelled at 37 °C or 45 °C and fully expanded in water. Deformation was calculated in the same method described in the TREx paper^7^, by taking the ratio of the radius of deformation to the radius of the gel. Squares are 5 mm. **(c)** Example images of *(****i****)* pre-expansion images of HEK cells stained with αTubulin (scale bar 1μm) imaged using a Nikon CFI Plan Apo VC 60× C WI (1.2 NA) objective and processed with SOFI compared to the same field of view *(****ii****)* post-expansion with MAGNIFY imaged with a Nikon APO LWD 20× (NA 0.95) WI Lambda S objective (scale bar 1 μm biological scale, 9.22 μm physical scale, expansion factor, 9.22×). *(****iii****)* Root mean square (RMS) length measurement error as a function of measurement length for pre-expansion versus post expansion images. Solid line, mean of channel; shaded area, standard error of mean; n = 3 technical replicates.

***
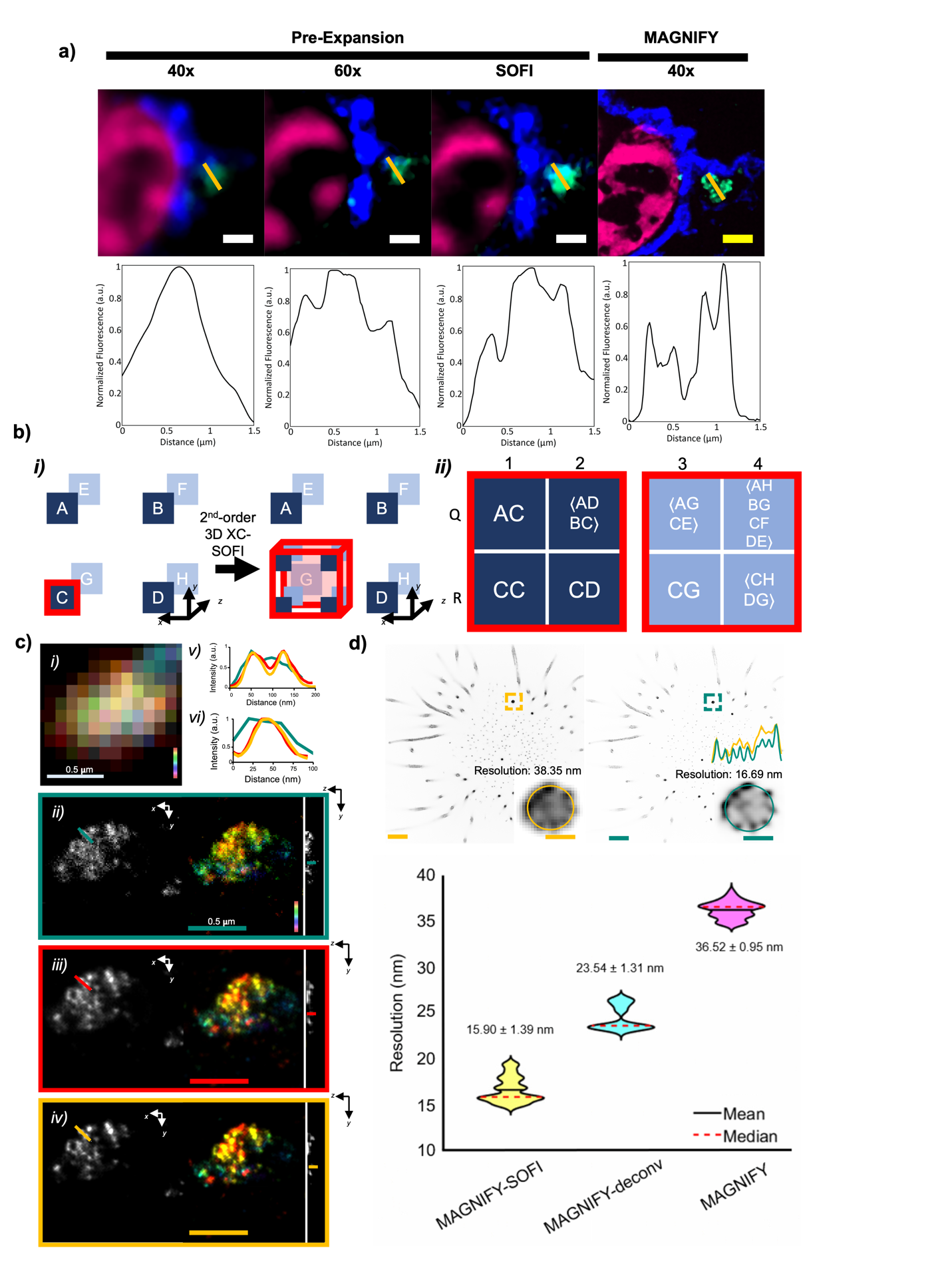
***

Supplementary Figure 5: Overview of SOFI **(a)** Second-order SOFI improves resolution pre-expansion. Top: Placenta tissue imaged pre-expansion at both 40× and 60× and post-expansion at 40×. Bottom: line profile of ATPIF channel indicated by orange line. 60× images were generated by performing a maximum intensity projection over 50 time points. This same set of images at 60× was processed using second-order SOFI with deconvolution. Resolution is seen to improve. Post-expansion images of the MAGNIFY processed sample show greater resolution enhancement. Scale bar: 1 µm (post-expansion 8.75 µm). Magenta: DAPI, Green: ATPIF, Blue: Pan-Keratin. **(b)** Generation of 3D XC-SOFI images **(*i*)** Each pixel in the original image becomes eight in the 2nd – order 3D XC-SOFI image by calculating its correlation with neighboring pixels. **(*ii*)** The generation of each new pixel in the 2nd – order 3D XC-SOFI image is given. For example, New Pixel R1 is given by calculating the autocorrelation of Pixel C in the original image, while New Pixel R2 is given by calculating the correlation between Pixels C and D. The values of New Pixels Q2, Q3, Q4, and R4 were calculated by averaging the correlations of multiple sets of Original Pixels. **(c)** Three-dimensional resolution improvement with MAGNIFY-SOFI. **(*i*)** A color-coded Z-projection of a pre-synapse stained for synaptophysin in a mouse brain before MAGNIFY. **(*ii*)** A separate synaptophysin stained pre-synapse in a mouse brain after MAGNIFY. Middle: Color-coded Z-projection of the synapse. Individual presynaptic vesicles are beginning to be resolved. Right: YZ view of the pre-synapse. Green line demarcates where line profile in **(*vi*)** is taken. Left: Single XY plane of the synapse. Green line demarcates where line profile in **(*v*)** is taken. **(*iii*)** As in **(*ii*)** but after 2nd-order 3D XC-SOFI. **(*iv*)** As in **(*ii*)** but after 3rd-order 3D XC-SOFI. **(v).** XY intensity line profiles showing increased resolving power of MAGNIFY-SOFI. **(*vi*)** YZ intensity line profiles showing increased resolving power of MAGNIFY-SOFI**. (****d)** Parameter-free image resolution estimation of MAGNIFY (top left panel) and MAGNIFY-SOFI (top right panel) images based on decorrelation analysis. Example images were acquired from a fully expanded human stem cell-derived lung organoid. Line profiles around an example cilium are shown for both MAGNIFY (yellow) and MAGNIFY-SOFI (green) in the right panel, demonstrating the resolution improvement. Bottom: violin plot of calculated resolutions of MANGIFY-SOFI (yellow), MAGNIFY with deconvolution (MAGNIFY-deconv; cyan), and MAGNIFY (magenta). N = 37 measurements from 4 technical replicates. Scale bar: 300 nm, inset: 100 nm. All in biological scales. Expansion factor: 10.5 ×.


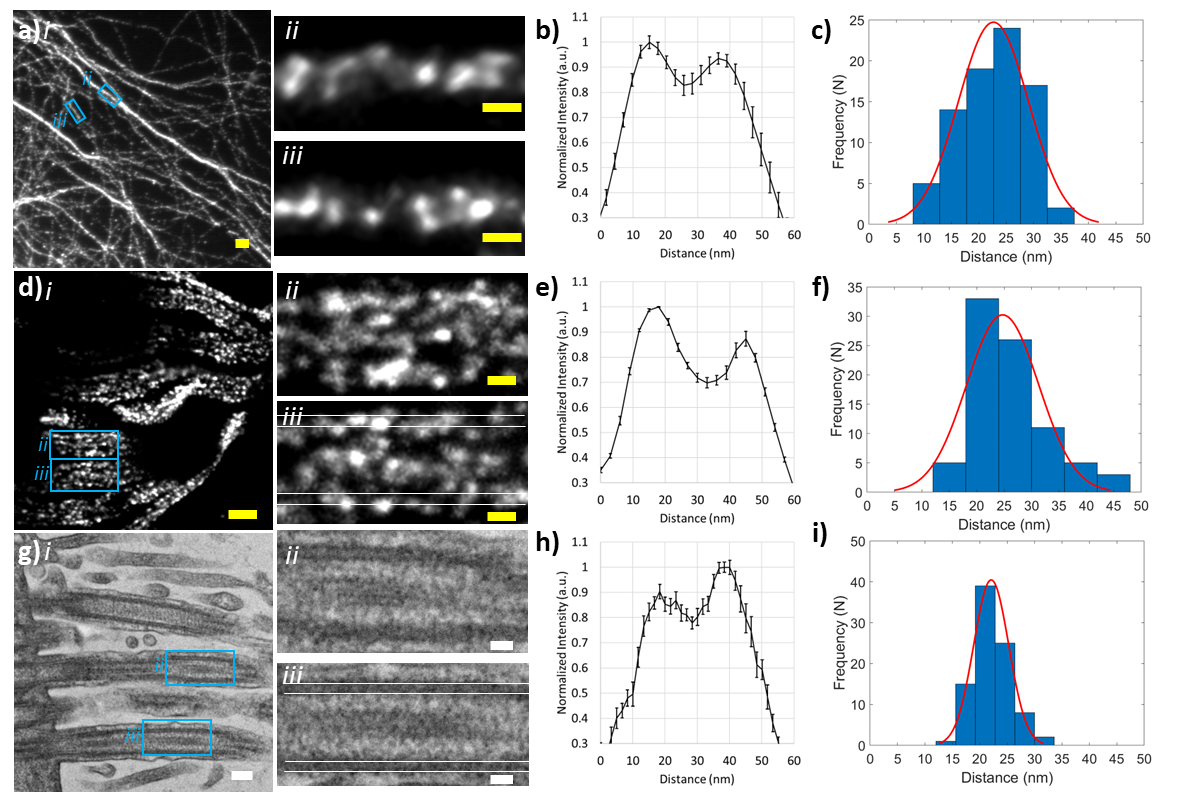


Supplementary Figure 6: Example images of tubulin structure in cell culture and cilia in human lung organoids. **(a)** Fluorescence images of alpha and beta tubulin stained MAGNIFY-processed fully expanded U2OS cells at 40× magnification. *ii* and *iii* show third order SOFI processed fluorescent images at 60× magnification of regions indicated in *i*. **(b)** Example average line profile averaged over 13 segments. Segments were averaged over 5 pixels. Error bars in terms of SEM. **(c)** Peak-to-peak distances of line profiles of 81 segments of expanded U2OS cells. The average over all distances was 22.68 nm ± 0.71 nm (mean ± s.e.m.). **(d)** Second order SOFI processed fluorescent images at 60× magnification of cilia in fully expanded MAGNIFY-processed human lung organoids stained with alpha and beta tubulin. *ii* and *iii* show zoomed in regions indicated in *i*. **(e)** Example average line profile averaged over 7 segments. Segments were averaged over 5 pixels. Error bars in terms of SEM. **(f)** Peak-to-peak distances of line profiles of 83 segments of expanded cilia. The average over all distances was 24.72 nm ± 0.72 nm (mean ± s.e.m.). **(g)** EM images of cilia in human lung organoids. *ii* and *iii* show zoomed in regions indicated in *i*. **(h)** Example average line profile averaged over 13 segments. Segments were averaged over 5 pixels. Error bars in terms of SEM. **(i) P**eak-to-peak distances of line profiles of 90 segments of unexpanded cilia imaged with EM. The average over all distances was 22.09 nm ± 0.34 nm (mean ± s.e.m.). Scale bars (biological scale): a,d,g *i*: 250 nm, a,d,g *ii-iii*: 50nm.

**
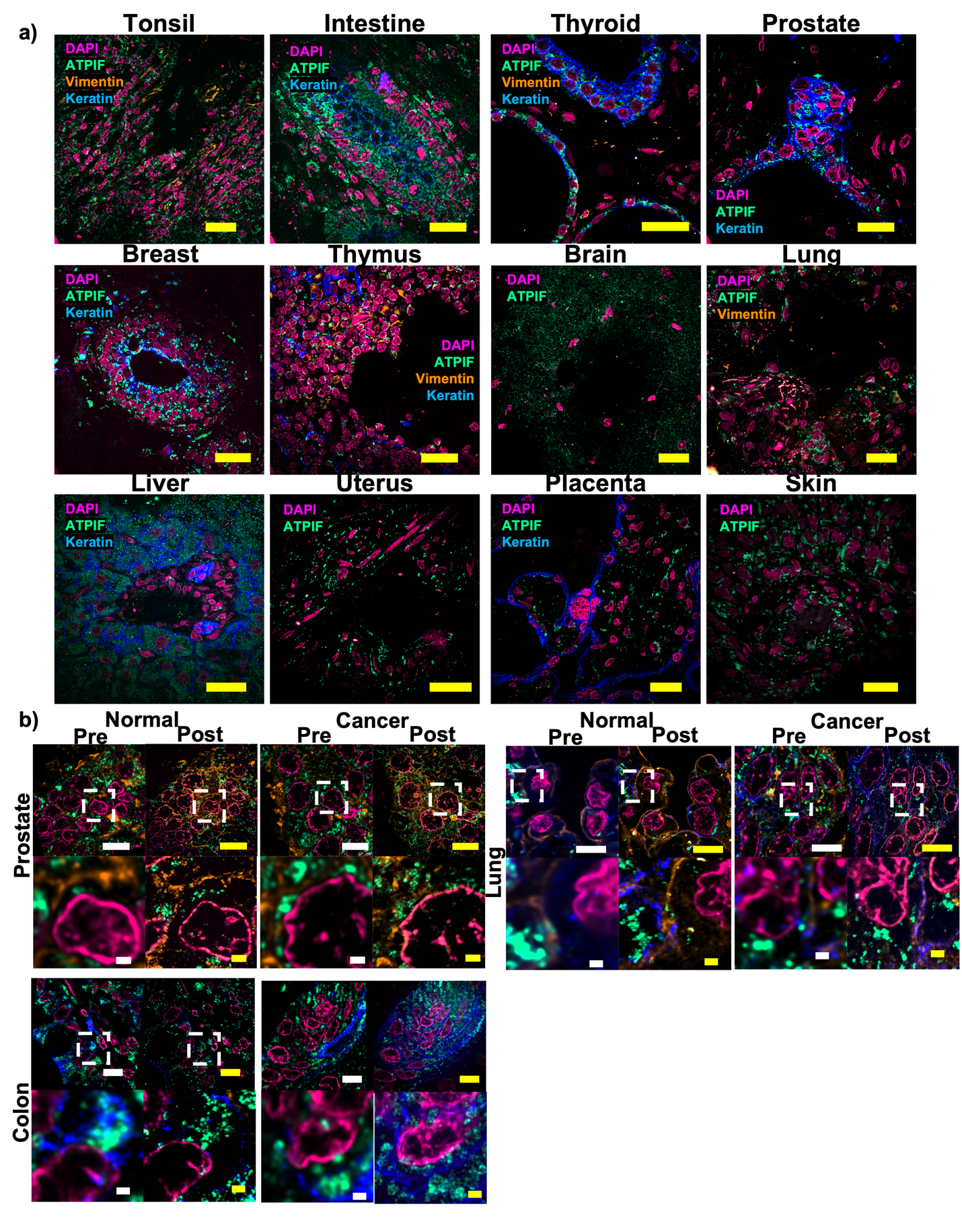
**

Supplementary Figure 7: Example images of MAGNIFY expansion of FFPE tissue. **(a)** Successful expansion of multiple human tissue types processed using the MAGNIFY framework. Images were obtained at 10× magnification. Scale bars are 25 µm (biological scale). Expansion factors are given in **Supplementary Table 1. (b)** Images of various tissue types for both normal (left images) and cancerous (right images) human tissues. Within each block of images for a given tissue disease type, there are four images shown. The left column shows two images, the top of which is a field of view (scale bar, 10 µm), and the bottom of which zooms into the area outlined in the top image by a red dash box (scale bar, 1 μm). The right column within the four images shows the same fields of view as are shown in the left column, but post-expansion (Scale bars are kept in the same biological scale: top images, 10 μm; bottom images, 1 µm; expansion factors: 10.4×, 10.8×, and 9.8× in water for prostate, lung, and colon, respectively); Magenta, DAPI; Green, ATPIF; Orange, WGA; Blue, PanK.

***
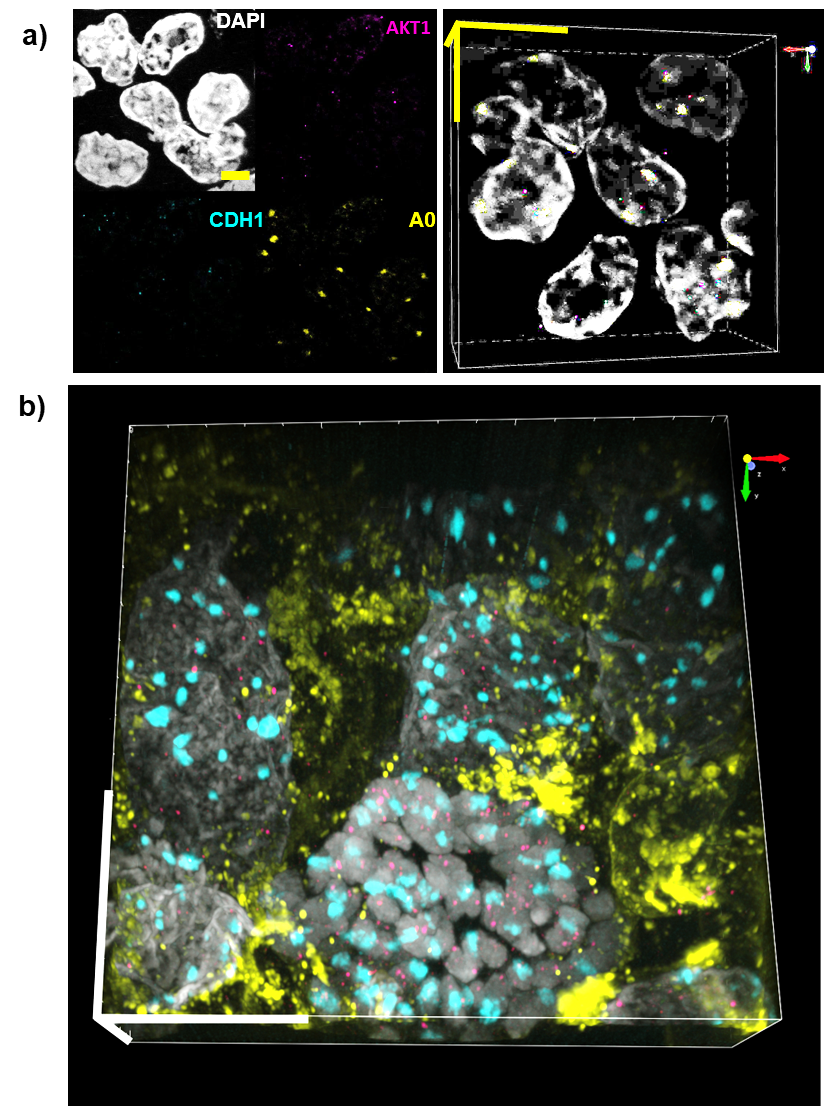
***

Supplementary Figure 8: DNA FISH with MAGNIFY using FFPE human tissue. After tissue section recovery, samples were gelled using the MAGNIFY gel and anchored with 0.25% methacrolein. After gelling, samples were treated with hot surfactant (1% w/v SDS, 8M Urea, 25 mM EDTA, 2× PBS, pH 7.5) for 48 hours at 80 °C, labeled with respective DNA FISH probes, and xpanded in 1xPBS before imaging.  **(a)** Normal human lymph node tissue. Scale bars: Left: 2.5 µm, Right: x: 5 µm, y: 5 µm, z: 5.7 µm. Expansion factor: 3.5× in 1× PBS. White, DAPI; Magenta, AKT1; Blue, CDH1, Yellow, A0. (**b**) 3D reconstruction of confocal images of expanded human urinary bladder cancer tissue section, homogenized by proteinase K digestion. Expansion factor 3.8× (in PBS buffer). Stain: Gray, DAPI; Cyan, DNA FISH probe against centromere binding protein B box motif CEPN-B; Magenta, DNA FISH probe against telomere motif TelC; Yellow, Wheat Germ Agglutinin. Scale bar: x: 20 μm; y: 20 μm; z: 20 μm.

## Supplementary Videos

Supplementary Video 1. 3D rendering of a fully expanded MAGNIFY processed human FFPE kidney tissue stained with DAPI (magenta), ACTN4 (orange), and WGA (blue) taken at 40× magnification.

Supplementary Video 2. 3D rendering of a fully expanded MAGNIFY processed human FFPE colon tissue stained with DAPI (magenta), ATPIF (green), and Cytokeratin Pan Type I/II (blue) taken at 40× magnification.

Supplementary Video 3. 3D rendering of a fully expanded MAGNIFY processed human FFPE placenta tissue stained with DAPI (magenta), ATPIF (green), and Cytokeratin Pan Type I/II (blue) taken at 40× magnification.

Supplementary Video 4. 3D rendering of a fully expanded MAGNIFY processed human FFPE breast tissue stained with DAPI (magenta), ATPIF (green), and Cytokeratin Pan Type I/II (blue) taken at 40× magnification.

Supplementary Video 5. 3D rendering of an expanded human urinary bladder cancer tissue section stained with DAPI (white), DNA FISH probe against centromere binding protein B box motif CEPN-B (cyan), DNA FISH probe against telomere motif TelC, and WGA (yellow) taken at 40× magnification.

Supplementary Video 6. 3D rendering of an SST neuron in MAGNIFY processed mouse brain stained with DAPI (white), anti-GFP (blue), synaptophysin (magenta) and PSD95 (green) expanded in 1x PBS and taken at 40× magnification.

Supplementary Video 7. 3D rendering of SST dendrites in MAGNIFY processed mouse brain stained with DAPI (white), anti-GFP (blue), synaptophysin (magenta) and PSD95 (green) expanded in 1x PBS and taken at 40× magnification.

Supplementary Video 8. 3D rendering of SST dendrites in MAGNIFY processed mouse brain stained with DAPI (white), anti-GFP (blue), synaptophysin (magenta) and PSD95 (green) expanded in 1x PBS and taken at 40× magnification.

Supplementary Video 9. 3D rendering of a MAGNIFY-SOFI image stack of fully expanded ependymal cilia and basal bodies from the ependymal cell lining in the adult mouse brain stained with NHS-ATTO-488 taken at 40× magnification.

## Supplementary Tables

### Supplementary Table 1: Condition optimization for different tissue types

| **Tissue Type** | **Fixation** | **Methacrolein** | **Hom. Time** | **Hom. Temp.** | **Expansion Factor** |
| --- | --- | --- | --- | --- | --- |
| Human Kidney | FFPE | 0.25% | 60 h | 80C | 8.64± 0.24 (N = 4)* |
| Human Lymph Node | FFPE | 0.25% | 60 h | 80C | 8.46± 0.47 (N = 5)* |
| Human Tonsil | FFPE | 0.25% | 60 h | 80C | 8.62 ± 0.27 (N = 6)* |
| Human Colon | FFPE | 0.25% | 60 h | 80C | 9.67 ± 0.35 (N = 3)* |
| Human Thyroid | FFPE | 0.25% | 60 h | 80C | 10.75 ± 0.35 (N = 3)* |
| Human Prostate | FFPE | 0.25% | 60 h | 80C | 10.38 ± 0.57 (N = 3)* |
| Human Breast | FFPE | 0.25% | 60 h | 80C | 9.03 ± 0.22 (N = 3)* |
| Human Thymus | FFPE | 0.25% | 60 h | 80C | 10.00 ± 0.37 (N = 3)* |
| Human Brain | FFPE | 0.25% | 10 h | 80C | 8.36 ± 0.25 (N = 3)* |
| Human Lung | FFPE | 0.25% | 60 h | 80C | 10.77± 0.61 (N = 3)* |
| Human Liver | FFPE | 0.25% | 60 h | 80C | 10.13± 0.60 (N = 3)* |
| Human Uterus | FFPE | 0.25% | 60 h | 80C | 8.00 ± 0.28 (N = 3)* |
| Human Placenta | FFPE | 0.25% | 60 h | 80C | 8.75 ± 0.37 (N = 3)* |
| Human Skin | FFPE | 0.25% | 60 h | 80C | 9.39 ± 0.30 (N = 3)* |
| Mouse Brain | PFA | 0.1% | 8 h | 80C | 11.18 ± 1.87 (N = 3)*^†^ |
| Lung Organoid | PFA | 0.1% | 8 h | 80C | 9.9 ± 2.1 (N=3) |
| HEK Cells | PFA/GA | 0.1% | 6 h | 80C | 9.22 ± 2.3 (N=6) ***** |

******* *Error in terms of s.e.m. over N technical replicates.*

† *Mouse brain expansion factor was calculated by measuring different nuclei sizes in mouse striatum before and after expansion.*

### Supplementary Table 2: Protein Retention for tissues under different anchoring and homogenization conditions.

|  | **Anchoring** | | **Homogenization** | | **Protein Retention** | | |
| --- | --- | --- | --- | --- | --- | --- | --- |
| **Tissue** | **Type** | **Conc.** | **Type** | **Time** | **%Retention** | **SEM** | **N** |
| **Kidney** | Methcarolein | 0.25% | Surfactant | 60h | 61.99 | 6.66 | 13 |
|  | Methcarolein | 0.05% | Surfactant | 60h | 13.63 | 1.86 | 8 |
|  | Methcarolein | 0.00% | Surfactant | 60h | 2.42 | 0.28 | 8 |
|  | AcX | 0.05 mg/mL | Surfactant | 60h | 2.94 | 0.57 | 14 |
|  | Methcarolein | 0.05% | ProK | 3h | 14.54 | 1.07 | 12 |
|  | AcX | 0.05 mg/mL | ProK | 3h | 3.08 | 0.46 | 9 |
| **Brain** | Methcarolein | 0.10% | Surfactant | 4h | 48.44 | 1.49 | 20 |
|  | Methcarolein | 0.10% | Surfactant | 8h | 23.76 | 0.69 | 20 |
|  | Methcarolein | 0.10% | Surfactant | 12h | 25.56 | 1.62 | 20 |
|  | Methcarolein | 0.10% | Surfactant | 16h | 14.20 | 1.06 | 20 |
|  | Methcarolein | 0.10% | Surfactant | 24h | 8.09 | 0.47 | 20 |
|  | AcX | 0.05 mg/mL | Surfactant | 8h | 12.82 | 0.77 | 20 |
|  | Methcarolein | 0.10% | ProK | 2h | 6.19 | 0.34 | 20 |
|  | AcX | 0.05 mg/mL | ProK | 2h | 7.72 | 0.41 | 20 |
| **Organoid** | Methacrolein | 0.10% | Surfactant | 8h | 76.23 | 8.46 | 13 |

***** Error in terms of s.e.m. over N technical replicates.

### Supplementary Table 3: Lipid retention in mouse brain under different anchoring and homogenization conditions.

| **Anchoring** | | **Homogenization** | | **Lipid Retention** | | |
| --- | --- | --- | --- | --- | --- | --- |
| **Type** | **Concentration** | **Type** | **Time** | **%Retention** | **SEM** | **N** |
| Methcarolein | 0.10% | Surfactant | 4h | 98.10 | 2.36 | 30 |
| Methcarolein | 0.10% | Surfactant | 8h | 77.05 | 1.80 | 20 |
| Methcarolein | 0.10% | Surfactant | 12h | 73.23 | 2.99 | 20 |
| Methcarolein | 0.10% | Surfactant | 16h | 63.60 | 3.06 | 20 |
| Methcarolein | 0.10% | Surfactant | 24h | 43.07 | 3.06 | 20 |
| AcX | 0.05 mg/mL | Surfactant | 8h | 74.50 | 2.47 | 20 |
| Methcarolein | 0.10% | ProK | 2h | 18.13 | 0.54 | 20 |
| AcX | 0.05 mg/mL | ProK | 2h | 17.06 | 0.80 | 20 |

***** Error in terms of s.e.m. over N technical replicates.

### Supplementary Table 4: Validated Primary Antibodies and fluorescent labels for Pre and Post MAGNIFY Staining

| **Host** | **Target** | **Vendor** | **Cat. Number** |
| --- | --- | --- | --- |
| Chicken | GFAP | Abcam | ab4674 |
| Chicken | GFP | Abcam | ab13970 |
| Chicken | NeuN | Millipore | ABN91 |
| Chicken | Tyrosine Hydroxalase | Abcam | ab76442 |
| Chicken | Vimentin | Abcam | ab24525 |
| Goat | PSD-95 | Abcam | ab12093 |
| Goat | CD4 | R&D Systems | AF-379-SP |
| Goat | Talin-1 | Novus | AF5456-SP |
| Mouse | Anti-Actin, α-Smooth Muscle | Sigma Aldrich | A5228 |
| Mouse | α-Tubulin | Sigma Aldrich | T6199 |
| Mouse | Bassoon | Abcam | ab82958 |
| Mouse | Cytokeratin Pan Type I/II | Invitrogen | MA5-13156 |
| Mouse | Synaptophysin | Invitrogen | MA1-213 |
| Mouse | CD11c | Abcam | ab11029 |
| Mouse | CD4 | Origene | UM870010 |
| Mouse | CD8 | Invitrogen | MA1-80231 |
| Mouse | CD8a (Alexa 488) | Invitrogen | 53-0008-82 |
| Mouse | VDACI | Abcam | ab14734 |
| Rabbit | α-Tubulin | Proteintech | 11224-1-AP |
| Rabbit | ACTN4 | Sigma Aldrich | HPA001873 |
| Rabbit | Alpha-Internexin (INA) | Sigma Aldrich | HPA008057 |
| Rabbit | Amyloid Precursor Protein (APP) | Sigma Aldrich | HPA001462 |
| Rabbit | Anti-ATPase Inhibitory Factor 1 (ATPIF1) | Millipore | ABC137 |
| Rabbit | Anti-ATPase Inhibitory Factor 1 (ATPIF1) | Proteintech | 12067-1-AP |
| Rabbit | GABRA1 | Proteintech | 12410-1-AP |
| Rabbit | GABRB1 | Proteintech | 20183-1-AP |
| Rabbit | GluR2 | Proteintech | 11994-1-AP |
| Rabbit | Synaptophysin | Proteintech | 17785-1-AP |
| Rabbit | CCR5 | Proteintech | 17476-1-AP |
| Rabbit | CD45 | Abcam | ab10558 |
| Rabbit | IL2 | Proteintech | 26156-1-AP |
| Rabbit | IL-6 | Proteintech | 21865-1-AP |
| Rabbit | TCR alpha Antibody | Novus | NBP2-52684 |
|  | Wheat Germ Agglutinin (WGA) CF555 | Biotium | 29076 |
|  | Wheat Germ Agglutinin (WGA) CF640R | Biotium | 29026 |
|  | NHS-ATTO-488 | Sigma Aldrich | 41698 |
|  | NHS-ATTO-532 | Sigma Aldrich | 88793 |
|  | Succinimidyl Ester (NHS) CF 555 | Biotium | 92130 |
|  | Cy3 NHS ester (non-sulfonated) | Glpbio Tech. | GC12618-25 |
|  | Vybrant™ DiD cell-labeling solution | Invitrogen | V-22887 |
|  | Vybrant™ DiO cell-labeling solution | Invitrogen | V-22886 |
|  | Vybrant™ DiI cell-labeling solution | Invitrogen | V-22885 |
|  | Lycopersicon Esculentum (Tomato) Lectin (LEL) | Vector Labs | DL-1174-1 |

### Supplementary Table 5: Secondary antibodies used for pre- and post-MAGNIFY staining.

| **Reactivity** | **Host** | **Conjugate** | **Vendor** | **Catalog Number** |
| --- | --- | --- | --- | --- |
| Rabbit | Goat | DyLight550 | Invitrogen | SA5-10033 |
| Rabbit | Goat | CF555 | Biotium | 20232 |
| Rabbit | Goat | Alexa Fluor 488 | Invitrogen | A11034 |
| Rabbit | Goat | CF640R | Biotium | 20202 |
| Rabbit | Goat | Fab Fragment AF488 | Jackson Immuno | 111-547-003 |
| Rabbit | Donkey | CF488A | Biotium | 20015 |
| Rabbit | Donkey | Fab Fragment AF488 | Jackson Immuno | 711-547-003 |
| Mouse | Goat | Alexa Fluor 488 | Invitrogen | A11001 |
| Mouse | Goat | CF568 | Biotium | 20301 |
| Mouse | Donkey | CF555 | Biotium | 20037 |
| Mouse | Donkey | CF640R | Biotium | 20177 |
| Mouse | Donkey | Fab Fragment AF488 | Jackson Immuno | 715-547-003 |
| Chicken | Goat | Alexa Fluor 488 | Invitrogen | A11039 |
| Chicken | Goat | DyLight 488 | Invitrogen | SA5-10070 |
| Chicken | Goat | DyLight 550 | Invitrogen | SA5-10033 |
| Chicken | Goat | CF488A | Biotium | 20020 |
| Chicken | Goat | CF555 | Biotium | 20034 |
| Chicken | Goat | CF640 | Biotium | 20084 |
| Goat | Donkey | CF647 | Biotium | 20829 |
| Streptavidin | | CF 640 | Biotium | 292037 |

### Supplementary Table 6: Comparison of expansion factors for different hydrogel chemistries for different tissue types.

| **Components Concentrations** | | | | | |  | **Expansion Factor** | |
| --- | --- | --- | --- | --- | --- | --- | --- | --- |
| **DMAA** | **SA** | **AA** | **Bis** | **NaCl** | **PBS** | **Anchoring** | **Human Kidney** | **Mouse Brain** |
| 4 | 34 | 10 | 0.01 | 1 | 1 | Methacrolein | 8.14 | 10.6 |
| 0 | 15 | 5 | 0.05 | 11.7 | 1 | AcX | 5.54 | N/A |
| 26.7 | 6.4 | 0 | 0 | 0 | 0 | AcX | 6.03 | N/A |

*PBS given in terms of 1x PBS concentration. N,N-Dimethylacrylamide (DMAA), Sodium acrylate (SA), Acrylamide (AA), N,N′-Methylenebisacrylamide (Bis), NaCl given in terms of w/v%.

### Supplementary Table 7: Exploration of different hydrogel chemistries and their respective expansion factors of blank gels in water.

| **Components Concentrations** | | | | | | | |  |
| --- | --- | --- | --- | --- | --- | --- | --- | --- |
| **DMAA** | **SA** | **AA** | **Bis** | **NaCl** | **PBS** | **APS** | **4HT** | **Ex Factor** |
| 1.5 | 37 | 15 | 0.01 | 1 | 1 | 1:50 | 1:500 | 6.5 (n=2) |
| 2 | 34 | 10 | 0.01 | 1 | 1 | 1:30 | 0 | 7.76 |
| 2 | 34 | 10 | 0.01 | 1 | 1 | 1:50 | 1:500 | 7.5 (n=2) |
| 4 | 30 | 10 | 0.01 | 1 | 1 | 1:30 | 0 | 6.45 |
| 4 | 34 | 10 | 0.01 | 1 | 1 | 1:30 | 0 | 7.86 |
| 4 | 33 | 10 | 0.01 | 1 | 1 | 1:50 | 1:500 | 7.5 (n=2) |
| 8 | 30 | 10 | 0.01 | 1 | 1 | 1:30 | 0 | 7.46 |
| 0 | 15 | 5 | 0.05 | 11.7 | 1 | 1:30 | 0 | 6.5 |
| 0 | 8.6 | 2.5 | 0.075 | 11.7 | 1 | 1:30 | 0 | 6 |

*PBS given in terms of 1x PBS concentration. N,N-Dimethylacrylamide (DMAA), Sodium acrylate (SA), Acrylamide (AA), N,N′-Methylenebisacrylamide (Bis), NaCl given in terms of w/v%.

### Supplementary Table 8: Gelling Conditions for MAGNIFY Gel.

| **Temp** | **APS** | **4HT** | **Ex Factor** |
| --- | --- | --- | --- |
| 45C | 1:50 | 1:500 | 7 |
| 45C | 1:40 | 1:500 | 8 |
| 45C | 1:30 | 1:500 | 8 |
| 45C | 1:50 | 1:250 | 7 |
| 45C | 1:40 | 1:250 | 8 |
| 45C | 1:30 | 1:250 | 7.5 |
| 37C | 1:50 | 1:500 | 8 |
| 37C | 1:40 | 1:500 | 8.5 |
| 37C | 1:30 | 1:500 | 8 |
| 37C | 1:50 | 1:250 | 7.5 |
| 37C | 1:40 | 1:250 | 8 |
| 37C | 1:30 | 1:250 | 7 |

### Supplementary Table 9: Deformation testing of different gel chemistries

| **Gel** | | | | | | | |  |  |  |
| --- | --- | --- | --- | --- | --- | --- | --- | --- | --- | --- |
|  | **DMAA** | **SA** | **AA** | **Bis** | **NaCl** | **PBS** | **Deformation** | | **EF** | **N** |
| **MAGNIFY** | 4 | 34 | 10 | 0.01 | 1 | 1 | 0.03± 0.01 | | 5.9±0.28 | 4 |
| **TREx (Low Bis)** | 0 | 11 | 14.5 | 0.005 | 0 | 1 | N/A | |  |  |
| **TREx (Med. Bis)** | 0 | 11 | 14.5 | 0.009 | 0 | 1 | 0.51± 0.10 | | 6.6±0.10 | 4 |
| **TREx (High Bis)** | 0 | 11 | 14.5 | 0.015 | 0 | 1 | 0.28± 0.09 | | 5.9±0.27 | 3 |
| **ExPath** | 0 | 15 | 5 | 0.05 | 11.7 | 1 | 0.04± 0.02 | | 4.3±0.14 | 4 |

*Errors given in terms of s.e.m.

**PBS given in terms of 1x PBS concentration. N,N-Dimethylacrylamide (DMAA), Sodium acrylate (SA), Acrylamide (AA), N,N′-Methylenebisacrylamide (Bis), NaCl given in terms of w/v%.

### Supplementary Table 10: Gelling Solution Recipe

| **Component** | **Stock Concentration*** | **Amount (mL)** | **Final Concentration** |
| --- | --- | --- | --- |
| N,N-dimethylacrylamide (DMAA) |  | 0.416 | 4 |
| Sodium acrylate (SA) | 50 | 6.8 | 34 |
| Acrylamide | 66.7 | 1.499 | 10 |
| N,N′-Methylenebisacrylamide | 2 | 0.05 | 0.01 |
| Sodium chloride | 30 | 0.333 | 1 |
| PBS | 10x | 1 | 1x |
| **Total Volume** |  | **10 mL** |  |
| * Note All concentrations given in g/100 mL except PBS | | |  |

### Supplementary Table 11: List of Chemicals and Reagents

| **Step** | **Reagent** | **Acronym** | **Vendor** | **Catalog Number** |
| --- | --- | --- | --- | --- |
| **Gelling** | N,N-dimethylacrylamide | DMAA | Sigma Aldrich | 274135 |
|  | Sodium acrylate | SA | AK Scientific | R624 |
|  | Sodium acrylate | SA | Santa Cruz Biotechnology | sc-236893B |
|  | Acrylamide | AA | Sigma Aldrich | A8887 |
|  | N,N′-Methylenebisacrylamide | BIS | Sigma Aldrich | M7279 |
|  | 4-hydroxy-TEMPO | 4HT | Sigma Aldrich | 176141 |
|  | Sodium chloride | NaCl | Sigma Aldrich | S6191 |
|  | Phosphate Buffered Saline, 10x Solution | PBS | Fischer Scientifc | BP399-1 |
|  | Acryloyl-X, SE | AcX | Invitrogen | A20770 |
|  | Ammonium persulfate | APS | Sigma Aldrich | A3678 |
|  | N,N,N′,N′-Tetramethylethylenediamine | TEMED | Sigma Aldrich | T9281 |
|  | Methacrolein |  | Sigma Aldrich | 133035 |
|  |  |  |  |  |
| **Homogenizing** | Ethylenediaminetetraacetic acid 0.5 M | EDTA | VWR | BDH7830-1 |
|  | Triton X-100 |  | Sigma Aldrich | T8787 |
|  | Tris Base |  | Fischer Scientifc | BP152-1 |
|  | Sodium chloride | NaCl | Sigma Aldrich | S6191 |
|  | Proteinase K (Molecular Biology Grade) | ProK | Thermo Scientific | EO0491 |
|  | Phosphate Buffered Saline, 10x Solution | PBS | Fischer Scientifc | BP399-1 |
|  | Sodium dodecyl sulfate | SDS | Sigma Aldrich | L3771 |
|  | Urea |  | Sigma Aldrich | U5378 |
|  | Glycine |  | Sigma Aldrich | G8898 |
|  |  |  |  |  |
| **Other** | Sodium citrate tribasic dihydrate |  | Sigma Aldrich | C8532-1KG |
|  | Xylenes |  | Sigma Aldrich | 214736 |
|  | Ethanol |  | Pharmco | 111000200 |
|  | SuperBlock Bloacking Buffer in PBS |  | Thermo Scientific | 37515 |
|  | Heparin |  | Sigma Aldrich | H3393 |
|  | DAPI |  | Thermo | 62248 |

### Supplementary Table 12: DNA FISH probes design

| Name | Target sequence | Oligo sequence |
| --- | --- | --- |
| AKT1-1 | ATGTCGATCCTAGAGTCCAGAACGGGGACTTCC | ATGTCGATCCTAGAGTCCAGAACGGGGACTTCCATAATCGCTAGGCACCTGGATT |
| AKT1-2 | GGAACGTGTTCAAAGAGGAGGAGACTCCGGACA | GGAACGTGTTCAAAGAGGAGGAGACTCCGGACAATAATCGCTAGGCACCTGGATT |
| AKT1-3 | AATCGCCAGAGAAGTAGAGAGTGTGTTTGGCGGG | AATCGCCAGAGAAGTAGAGAGTGTGTTTGGCGGGATAATCGCTAGGCACCTGGATT |
| AKT1-4 | ACCGGATCATCTTCACAACAGTCCCATTAACTAGACGC | ACCGGATCATCTTCACAACAGTCCCATTAACTAGACGCATAATCGCTAGGCACCTGGATT |
| AKT1-5 | GGTCTCATTCTTCTCTGCCTTGGAGTCCGGGA | GGTCTCATTCTTCTCTGCCTTGGAGTCCGGGAATAATCGCTAGGCACCTGGATT |
| AKT1-6 | AATGGAGAATGTAGTGGAGGAGTCACCCCAGTCAAG | AATGGAGAATGTAGTGGAGGAGTCACCCCAGTCAAGATAATCGCTAGGCACCTGGATT |
| AKT1-7 | AACTGCAGCTTGGACCCCTGGAATGACACT | AACTGCAGCTTGGACCCCTGGAATGACACTATAATCGCTAGGCACCTGGATT |
| AKT1-8 | GCACTTACAGCCACTGAGACTAGCTTAGGGACGG | GCACTTACAGCCACTGAGACTAGCTTAGGGACGGATAATCGCTAGGCACCTGGATT |
| AKT1-9 | GAACTCAGATGTGACTGCTCCCTTCCTGCCC | GAACTCAGATGTGACTGCTCCCTTCCTGCCCATAATCGCTAGGCACCTGGATT |
| AKT1-10 | ACCCTTCACTGGTTTCTCTTCATCCCTGTCTCTGC | ACCCTTCACTGGTTTCTCTTCATCCCTGTCTCTGCATAATCGCTAGGCACCTGGATT |
| AKT1-11 | TCACTTGGTGCTTAAAAGTTGGCAGGACGCAAGT | TCACTTGGTGCTTAAAAGTTGGCAGGACGCAAGTATAATCGCTAGGCACCTGGATT |
| AKT1-12 | TGGGTCTCAAGTGCAGGAATGACAGGACACC | TGGGTCTCAAGTGCAGGAATGACAGGACACCATAATCGCTAGGCACCTGGATT |
| AKT1-13 | TTTTGTCGGAAACATCTGGCCACAGAGCACCT | TTTTGTCGGAAACATCTGGCCACAGAGCACCTATAATCGCTAGGCACCTGGATT |
| AKT1-14 | GGGGTGTTCTTGAAGAGAGGACTCTGCTTTCCCT | GGGGTGTTCTTGAAGAGAGGACTCTGCTTTCCCTATAATCGCTAGGCACCTGGATT |
| AKT1-15 | GGCCTCCTTGGAGACAGCCAGTGCAAAATAAGC | GGCCTCCTTGGAGACAGCCAGTGCAAAATAAGCATAATCGCTAGGCACCTGGATT |
| AKT1-16 | AAGAGGTCCAACCACTTCAAGAACAGCCCGC | AAGAGGTCCAACCACTTCAAGAACAGCCCGCATAATCGCTAGGCACCTGGATT |
| AKT1-17 | CCCTCTTACCCTCTGCTTCCTCCCTGAATTCCT | CCCTCTTACCCTCTGCTTCCTCCCTGAATTCCTATAATCGCTAGGCACCTGGATT |
| AKT1-18 | GGATGGTGGACAGATACCAGGAACTCTTCCTCGG | GGATGGTGGACAGATACCAGGAACTCTTCCTCGGATAATCGCTAGGCACCTGGATT |
| AKT1-19 | TACAAAGTCTGAGCTGGGAGGGAGCAGAGGT | TACAAAGTCTGAGCTGGGAGGGAGCAGAGGTATAATCGCTAGGCACCTGGATT |
| AKT1-20 | GGGGAGAGAGTCCTTCTCTTGGTCAGCCCC | GGGGAGAGAGTCCTTCTCTTGGTCAGCCCCATAATCGCTAGGCACCTGGATT |
| AKT1-21 | AGAAAGGGGTCTGTGTCCCACTTACTCATTCCATACC | AGAAAGGGGTCTGTGTCCCACTTACTCATTCCATACCATAATCGCTAGGCACCTGGATT |
| AKT1-22 | GGGGACATCCAGAGGTCTTTGAGTCCAGCC | GGGGACATCCAGAGGTCTTTGAGTCCAGCCATAATCGCTAGGCACCTGGATT |
| AKT1-23 | GCTTTCCATCCTGCTAAGTACTTGGGGCATTTCCC | GCTTTCCATCCTGCTAAGTACTTGGGGCATTTCCCATAATCGCTAGGCACCTGGATT |
| AKT1-24 | TCAGAGGGGAAATGAGGAAGCCATGCAGGATCA | TCAGAGGGGAAATGAGGAAGCCATGCAGGATCAATAATCGCTAGGCACCTGGATT |
| AKT1-25 | AGGAACACCATGGACAGGGAGAGCAAACGG | AGGAACACCATGGACAGGGAGAGCAAACGGATAATCGCTAGGCACCTGGATT |
| AKT1-26 | AGCCTAGAATAAGGAGAGGCCCAGGTCCAGG | AGCCTAGAATAAGGAGAGGCCCAGGTCCAGGATAATCGCTAGGCACCTGGATT |
| AKT1-27 | TAGCCAGGCGTGGCCTCACATTCAGCT | TAGCCAGGCGTGGCCTCACATTCAGCTATAATCGCTAGGCACCTGGATT |
| AKT1-28 | GTAGTAGCCCCAGGGTCTGTGAGTGCCTG | GTAGTAGCCCCAGGGTCTGTGAGTGCCTGATAATCGCTAGGCACCTGGATT |
| AKT1-29 | TTTTGCTCCTCTGTCCTGCTAGGGTGGGC | TTTTGCTCCTCTGTCCTGCTAGGGTGGGCATAATCGCTAGGCACCTGGATT |
| AKT1-30 | AAGTCACTCTCTTCTGCCCCAGGCGGAATC | AAGTCACTCTCTTCTGCCCCAGGCGGAATCATAATCGCTAGGCACCTGGATT |
| AKT1-31 | GTTTCACATCTGGTAGTGGGGAGACCCCAAACAC | GTTTCACATCTGGTAGTGGGGAGACCCCAAACACATAATCGCTAGGCACCTGGATT |
| AKT1-32 | AAAAGTAGGTGTCACAAGATGGGGCATTGTGGGATG | AAAAGTAGGTGTCACAAGATGGGGCATTGTGGGATGATAATCGCTAGGCACCTGGATT |
| AKT1-33 | AGAGCAAGGTCATTGAGCTCCTTGGGCCT | AGAGCAAGGTCATTGAGCTCCTTGGGCCTATAATCGCTAGGCACCTGGATT |
| AKT1-34 | CAGTTTCCCCATCTACACCAGGGAGCGGT | CAGTTTCCCCATCTACACCAGGGAGCGGTATAATCGCTAGGCACCTGGATT |
| AKT1-35 | ATAGCCCCTCTTGTTGCCGAGAGCAGGT | ATAGCCCCTCTTGTTGCCGAGAGCAGGTATAATCGCTAGGCACCTGGATT |
| AKT1-36 | TCCTTGGGAGGTGAGCGTCATCTCTGGGA | TCCTTGGGAGGTGAGCGTCATCTCTGGGAATAATCGCTAGGCACCTGGATT |
| AKT1-37 | TGTGTGTGCTCTGAGTCAGAGGAGCTTCAGGG | TGTGTGTGCTCTGAGTCAGAGGAGCTTCAGGGATAATCGCTAGGCACCTGGATT |
| AKT1-38 | GGCCTCTGGGTCTGCACATCTAACAGGGAG | GGCCTCTGGGTCTGCACATCTAACAGGGAGATAATCGCTAGGCACCTGGATT |
| AKT1-39 | CTTTGGCCAGAGGCTGTGTGGCTCTTTTGAGG | CTTTGGCCAGAGGCTGTGTGGCTCTTTTGAGGATAATCGCTAGGCACCTGGATT |
| AKT1-40 | TACTTTCCCCAAACAGCTTGCTCACCTTGCCC | TACTTTCCCCAAACAGCTTGCTCACCTTGCCCATAATCGCTAGGCACCTGGATT |
| AKT1-41 | TTCCTACCCCAGTGTGTTACTTCGCTTTGGTGAGC | TTCCTACCCCAGTGTGTTACTTCGCTTTGGTGAGCATAATCGCTAGGCACCTGGATT |
| AKT1-42 | AAAACAACAAAAGAGGAAGCCGAGGTGGCCTTGA | AAAACAACAAAAGAGGAAGCCGAGGTGGCCTTGAATAATCGCTAGGCACCTGGATT |
| AKT1-43 | TGGAATGATTCCTGTGCTGGGGCCTAGACC | TGGAATGATTCCTGTGCTGGGGCCTAGACCATAATCGCTAGGCACCTGGATT |
| AKT1-44 | CCCGTACACCTTCCACTCTCAGGAGAAGCCT | CCCGTACACCTTCCACTCTCAGGAGAAGCCTATAATCGCTAGGCACCTGGATT |
| AKT1-45 | TGCCGCCTTTAGGTGTGTTTTCCTTGAACCTTAAGG | TGCCGCCTTTAGGTGTGTTTTCCTTGAACCTTAAGGATAATCGCTAGGCACCTGGATT |
| AKT1-46 | AGGAGAATGGAAAGCTGAGACCCAGGTGCTTCC | AGGAGAATGGAAAGCTGAGACCCAGGTGCTTCCATAATCGCTAGGCACCTGGATT |
| AKT1-47 | ATGGTGGAGCGGAAGGAGGTGAAGAATTTGCA | ATGGTGGAGCGGAAGGAGGTGAAGAATTTGCAATAATCGCTAGGCACCTGGATT |
| AKT1-48 | GTTTGTGTCAAGTTACAACCCCTGCCTTGGCG | GTTTGTGTCAAGTTACAACCCCTGCCTTGGCGATAATCGCTAGGCACCTGGATT |
| AKT1-49 | ACTTTGAGAGAATGTTCAGGGAGCTGAGGACGTGG | ACTTTGAGAGAATGTTCAGGGAGCTGAGGACGTGGATAATCGCTAGGCACCTGGATT |
| AKT1-50 | CCTGGGGACCCTCAGGATAGTGAAGGAGAAGAC | CCTGGGGACCCTCAGGATAGTGAAGGAGAAGACATAATCGCTAGGCACCTGGATT |
| AKT1-51 | CCCCGTGGCTCCCTGGAAAGTAGAATGCC | CCCCGTGGCTCCCTGGAAAGTAGAATGCCATAATCGCTAGGCACCTGGATT |
| AKT1-52 | AGACCTCTTCCCAGCCTCCTGTCCATGGT | AGACCTCTTCCCAGCCTCCTGTCCATGGTATAATCGCTAGGCACCTGGATT |
| AKT1-53 | AGAGATGAGAGACATGTGACGCTCCCTGCTCC | AGAGATGAGAGACATGTGACGCTCCCTGCTCCATAATCGCTAGGCACCTGGATT |
| AKT1-54 | GCCCTGCTGTGGCTTTATTCCTTACCTGTAGCG | GCCCTGCTGTGGCTTTATTCCTTACCTGTAGCGATAATCGCTAGGCACCTGGATT |
| AKT1-55 | GTTGGGCATGCAGGTGTGACTGAGTGGC | GTTGGGCATGCAGGTGTGACTGAGTGGCATAATCGCTAGGCACCTGGATT |
| AKT1-56 | CTTTCTTTGAGGCCTGCGGTCCTCCGACT | CTTTCTTTGAGGCCTGCGGTCCTCCGACTATAATCGCTAGGCACCTGGATT |
| AKT1-57 | CTGAAGCTTCTAGTTGGGAGGGGCAGAGGC | CTGAAGCTTCTAGTTGGGAGGGGCAGAGGCATAATCGCTAGGCACCTGGATT |
| AKT1-58 | CTCGTAACCATGCAGGAGACTCACTGTGACTTGTCC | CTCGTAACCATGCAGGAGACTCACTGTGACTTGTCCATAATCGCTAGGCACCTGGATT |
| AKT1-59 | TTCAGATTAGGCCATAGGTGAGTGGCCTGGAGC | TTCAGATTAGGCCATAGGTGAGTGGCCTGGAGCATAATCGCTAGGCACCTGGATT |
| AKT1-60 | GTGTATTCCAGTGTGGCTGTGTCCCAGGTGT | GTGTATTCCAGTGTGGCTGTGTCCCAGGTGTATAATCGCTAGGCACCTGGATT |
| AKT1-61 | ATGTATGTGTGCCCCAGGTGTGGCTGTGC | ATGTATGTGTGCCCCAGGTGTGGCTGTGCATAATCGCTAGGCACCTGGATT |
| AKT1-62 | TGGGTGTGCTCCAGGTGGCTGTATTTCCC | TGGGTGTGCTCCAGGTGGCTGTATTTCCCATAATCGCTAGGCACCTGGATT |
| AKT1-63 | AAAAGCAGGACATTTCTACACTAGGTGGGACAAGCAGG | AAAAGCAGGACATTTCTACACTAGGTGGGACAAGCAGGATAATCGCTAGGCACCTGGATT |
| AKT1-64 | ATCTCCATACCCCTCATCCTCTGAGGCCTGG | ATCTCCATACCCCTCATCCTCTGAGGCCTGGATAATCGCTAGGCACCTGGATT |
| AKT1-65 | CTGAGTGTATGTGGCCAGACCAGGTCAAGTGG | CTGAGTGTATGTGGCCAGACCAGGTCAAGTGGATAATCGCTAGGCACCTGGATT |
| AKT1-66 | AGTTCCAGGTCACTGTGTTTGGGGACGATTCTCC | AGTTCCAGGTCACTGTGTTTGGGGACGATTCTCCATAATCGCTAGGCACCTGGATT |
| AKT1-67 | CACCTTGCTCACCTTTCAAACACTCCTTGGCACC | CACCTTGCTCACCTTTCAAACACTCCTTGGCACCATAATCGCTAGGCACCTGGATT |
| AKT1-68 | GGGTGACTTGTTCCTGCTGAGTTAGGGCTTCTG | GGGTGACTTGTTCCTGCTGAGTTAGGGCTTCTGATAATCGCTAGGCACCTGGATT |
| AKT1-69 | AGATTGTGTCAGCCCTGGACTACCTGCACTCG | AGATTGTGTCAGCCCTGGACTACCTGCACTCGATAATCGCTAGGCACCTGGATT |
| AKT1-70 | CCTTGCTTTCAGGGCTGCTCAAGAAGGACCC | CCTTGCTTTCAGGGCTGCTCAAGAAGGACCCATAATCGCTAGGCACCTGGATT |
| AKT1-71 | GCGGTACCGACACTGTGGCCTTGTTTCC | GCGGTACCGACACTGTGGCCTTGTTTCCATAATCGCTAGGCACCTGGATT |
| AKT1-72 | CATGCAGCATCGCTTCTTTGCCGGTATCGT | CATGCAGCATCGCTTCTTTGCCGGTATCGTATAATCGCTAGGCACCTGGATT |
| AKT1-73 | CGCATGCTCCCCACATATCCACACTCACGC | CGCATGCTCCCCACATATCCACACTCACGCATAATCGCTAGGCACCTGGATT |
| AKT1-74 | AGCCTGCTGCAGTCCTGGTACAAGGAGG | AGCCTGCTGCAGTCCTGGTACAAGGAGGATAATCGCTAGGCACCTGGATT |
| AKT1-75 | TCATCTTTCAGGGACCCTAGGAGCCCTGGC | TCATCTTTCAGGGACCCTAGGAGCCCTGGCATAATCGCTAGGCACCTGGATT |
| AKT1-76 | GCTGGGTTCGGAAGCCTGCACTCTGAGA | GCTGGGTTCGGAAGCCTGCACTCTGAGAATAATCGCTAGGCACCTGGATT |
| AKT1-77 | GCCCAGCCTTGACCAGAGACCTTGCTAATTGA | GCCCAGCCTTGACCAGAGACCTTGCTAATTGAATAATCGCTAGGCACCTGGATT |
| AKT1-78 | AGATCCAGGTGCTTTGAAGGTCTTGAGCACACTTG | AGATCCAGGTGCTTTGAAGGTCTTGAGCACACTTGATAATCGCTAGGCACCTGGATT |
| AKT1-79 | AAAAGGTTCCTTTTGGAGAGTGCCAATGATCAGGGTG | AAAAGGTTCCTTTTGGAGAGTGCCAATGATCAGGGTGATAATCGCTAGGCACCTGGATT |
| AKT1-80 | TGGGCTGTCTGTCACCAGCTATCTGTCATCTCT | TGGGCTGTCTGTCACCAGCTATCTGTCATCTCTATAATCGCTAGGCACCTGGATT |
| AKT1-81 | TTTTGGCTCACTTTGCTGGGTGGAAGAGTGGG | TTTTGGCTCACTTTGCTGGGTGGAAGAGTGGGATAATCGCTAGGCACCTGGATT |
| AKT1-82 | ATGAAGGAGACCATTGGACCACGTGGCCA | ATGAAGGAGACCATTGGACCACGTGGCCAATAATCGCTAGGCACCTGGATT |
| AKT1-83 | GGTAGGGCAGTGAATGAGACAGACCACCAGGA | GGTAGGGCAGTGAATGAGACAGACCACCAGGAATAATCGCTAGGCACCTGGATT |
| AKT1-84 | AGGACAGATAGGGCAGGGCCCAGAGTTGG | AGGACAGATAGGGCAGGGCCCAGAGTTGGATAATCGCTAGGCACCTGGATT |
| AKT1-85 | CTGGCGATGGCAATTGGCCCCTCTCTAAGG | CTGGCGATGGCAATTGGCCCCTCTCTAAGGATAATCGCTAGGCACCTGGATT |
| AKT1-86 | CCAAGAACAGCCACATCTGGAGCAGCCC | CCAAGAACAGCCACATCTGGAGCAGCCCATAATCGCTAGGCACCTGGATT |
| AKT1-87 | CCCCTCTGACAATATAGGATTGCCTTAGGGCCATTTCT | CCCCTCTGACAATATAGGATTGCCTTAGGGCCATTTCTATAATCGCTAGGCACCTGGATT |
| AKT1-88 | CTCTGAGAATTTCCCGACCCTCCTAGCAGCCC | CTCTGAGAATTTCCCGACCCTCCTAGCAGCCCATAATCGCTAGGCACCTGGATT |
| AKT1-89 | GGGGAAAGTGTTCTAGGGCAAAGAAGCATTGAGAAGATGT | GGGGAAAGTGTTCTAGGGCAAAGAAGCATTGAGAAGATGTATAATCGCTAGGCACCTGGATT |
| AKT1-90 | TTTTGGTGAGAGAGTTTAGGTAAGGTACCCAGAGCCATGT | TTTTGGTGAGAGAGTTTAGGTAAGGTACCCAGAGCCATGTATAATCGCTAGGCACCTGGATT |
| AKT1-91 | ATCAGGCAGTAAGTCACTGAACCACGATTTGAATTCAGCC | ATCAGGCAGTAAGTCACTGAACCACGATTTGAATTCAGCCATAATCGCTAGGCACCTGGATT |
| AKT1-92 | TTTCACCGGATGGACTGCATCTTTATTTCCTAAACCTGGC | TTTCACCGGATGGACTGCATCTTTATTTCCTAAACCTGGCATAATCGCTAGGCACCTGGATT |
| AKT1-93 | TCTTTCACAAAAGGGTTATTATCAACTGTGGGCCTCTGGA | TCTTTCACAAAAGGGTTATTATCAACTGTGGGCCTCTGGAATAATCGCTAGGCACCTGGATT |
| AKT1-94 | TCCAGACTTGTTCCCTCCTAAGTTCTAGTGATCTCATGCC | TCCAGACTTGTTCCCTCCTAAGTTCTAGTGATCTCATGCCATAATCGCTAGGCACCTGGATT |
| AKT1-95 | CCCCTCAGATGAAGCAGTTGCCTAATTAGTAAGTTCCCT | CCCCTCAGATGAAGCAGTTGCCTAATTAGTAAGTTCCCTATAATCGCTAGGCACCTGGATT |
| AKT1-96 | CATAGACCATGAACGAGTTTGAGTACCTGAAGCTGCTGG | CATAGACCATGAACGAGTTTGAGTACCTGAAGCTGCTGGATAATCGCTAGGCACCTGGATT |
| AKT1-97 | TCACCTTATAGTCACCCTTCATCCTGGGTCATTGAGAGT | TCACCTTATAGTCACCCTTCATCCTGGGTCATTGAGAGTATAATCGCTAGGCACCTGGATT |
| AKT1-98 | AAAATAAAGTTATCACCTCCTGGTAGCAGGGAGGGTCTCT | AAAATAAAGTTATCACCTCCTGGTAGCAGGGAGGGTCTCTATAATCGCTAGGCACCTGGATT |
| AKT1-99 | TACACGAAACATGACACAGTAAATGATGTTCCGAGGGTGA | TACACGAAACATGACACAGTAAATGATGTTCCGAGGGTGAATAATCGCTAGGCACCTGGATT |
| AKT1-100 | AGCATTGCTGAAAGTAACCAAGGTATGGAAATGGTCAGGC | AGCATTGCTGAAAGTAACCAAGGTATGGAAATGGTCAGGCATAATCGCTAGGCACCTGGATT |
| AKT1_488 | /5Alex488N/AATCCAGGTGCCTAGCGATT |  |
| CDH1-1 | GCCCTCTCTTGGTTACTGGGCTGTGGCT | GCCCTCTCTTGGTTACTGGGCTGTGGCTTATGTGCGGCATTGACTAAGAG |
| CDH1-2 | AGAGCAAGGCAGGGGCTAGAAACAAGCTTGT | AGAGCAAGGCAGGGGCTAGAAACAAGCTTGTTATGTGCGGCATTGACTAAGAG |
| CDH1-3 | ATCTGAGCATGCGTCTGGGGTGTCCCA | ATCTGAGCATGCGTCTGGGGTGTCCCATATGTGCGGCATTGACTAAGAG |
| CDH1-4 | GGGGACCCTGGGACTGGTGATTTAGTGGG | GGGGACCCTGGGACTGGTGATTTAGTGGGTATGTGCGGCATTGACTAAGAG |
| CDH1-5 | TCCTTTGCTTGCTCTGCAATCTGGCCTTGG | TCCTTTGCTTGCTCTGCAATCTGGCCTTGGTATGTGCGGCATTGACTAAGAG |
| CDH1-6 | AAAGAAGACCAGTGGGCCGCCCTCCT | AAAGAAGACCAGTGGGCCGCCCTCCTTATGTGCGGCATTGACTAAGAG |
| CDH1-7 | AAGGCCATGTAAAGAAGGCAGGCTGCTGC | AAGGCCATGTAAAGAAGGCAGGCTGCTGCTATGTGCGGCATTGACTAAGAG |
| CDH1-8 | GCACCTGTCCCAATCCAATCCTTGCTGGG | GCACCTGTCCCAATCCAATCCTTGCTGGGTATGTGCGGCATTGACTAAGAG |
| CDH1-9 | AGGCTAGACCCTGAGGTTACGGCAGATTGGA | AGGCTAGACCCTGAGGTTACGGCAGATTGGATATGTGCGGCATTGACTAAGAG |
| CDH1-10 | GGGCAGGCAGGGAGATCTCAAACCTGAGT | GGGCAGGCAGGGAGATCTCAAACCTGAGTTATGTGCGGCATTGACTAAGAG |
| CDH1-11 | GCTCCCTCTCACCCAGCAAACCAGCC | GCTCCCTCTCACCCAGCAAACCAGCCTATGTGCGGCATTGACTAAGAG |
| CDH1-12 | GGGTGGACCGGAACGGGTTTGTTGTGG | GGGTGGACCGGAACGGGTTTGTTGTGGTATGTGCGGCATTGACTAAGAG |
| CDH1-13 | CAGAGGTTGCACTGCTTGCCACCAAGTCAC | CAGAGGTTGCACTGCTTGCCACCAAGTCACTATGTGCGGCATTGACTAAGAG |
| CDH1-14 | GCTCAGGACCCCGTGGGATAATTGGCCA | GCTCAGGACCCCGTGGGATAATTGGCCATATGTGCGGCATTGACTAAGAG |
| CDH1-15 | TTTGCTTAAATGTCACTCCCGCCCTCAGGGA | TTTGCTTAAATGTCACTCCCGCCCTCAGGGATATGTGCGGCATTGACTAAGAG |
| CDH1-16 | AAAATTATTTGGCAGGGGCAGGGCACGGT | AAAATTATTTGGCAGGGGCAGGGCACGGTTATGTGCGGCATTGACTAAGAG |
| CDH1-17 | AGTGGGAGAGCCTGGCCGTATGTCTGG | AGTGGGAGAGCCTGGCCGTATGTCTGGTATGTGCGGCATTGACTAAGAG |
| CDH1-18 | CAAGCCCTCTGCTAGGCATTGGGGATGC | CAAGCCCTCTGCTAGGCATTGGGGATGCTATGTGCGGCATTGACTAAGAG |
| CDH1-19 | GCCCTGAGTCACCGACATTGAGAGGCCT | GCCCTGAGTCACCGACATTGAGAGGCCTTATGTGCGGCATTGACTAAGAG |
| CDH1-20 | TGCTTCCAGTGCCTGGTACGTAGCAACCA | TGCTTCCAGTGCCTGGTACGTAGCAACCATATGTGCGGCATTGACTAAGAG |
| CDH1-21 | GGGGCGGGTAGTGTAAGTGTCAAAGGAGCC | GGGGCGGGTAGTGTAAGTGTCAAAGGAGCCTATGTGCGGCATTGACTAAGAG |
| CDH1-22 | TCATGCCAAGGTCCACCCATGCAGCC | TCATGCCAAGGTCCACCCATGCAGCCTATGTGCGGCATTGACTAAGAG |
| CDH1-23 | TTCCGCACAGTGTGTGAGTGCCTTCTGTGT | TTCCGCACAGTGTGTGAGTGCCTTCTGTGTTATGTGCGGCATTGACTAAGAG |
| CDH1-24 | GGGGATGCCCCAAGCCTAGAGAGGTGG | GGGGATGCCCCAAGCCTAGAGAGGTGGTATGTGCGGCATTGACTAAGAG |
| CDH1-25 | TGGCAGGACTGCAGAAGGACAATAGTGTGGC | TGGCAGGACTGCAGAAGGACAATAGTGTGGCTATGTGCGGCATTGACTAAGAG |
| CDH1-26 | TGGTTGGGTGAGGCCCTTTTGGCCTG | TGGTTGGGTGAGGCCCTTTTGGCCTGTATGTGCGGCATTGACTAAGAG |
| CDH1-27 | AGGGGAGGGCGTTCTAGATGGAGGGAACA | AGGGGAGGGCGTTCTAGATGGAGGGAACATATGTGCGGCATTGACTAAGAG |
| CDH1-28 | CCTCTGGTCCTTTAGCCCAGATCCACCGG | CCTCTGGTCCTTTAGCCCAGATCCACCGGTATGTGCGGCATTGACTAAGAG |
| CDH1-29 | TTTCCAATGGCGCTGCTGGCTCTGGC | TTTCCAATGGCGCTGCTGGCTCTGGCTATGTGCGGCATTGACTAAGAG |
| CDH1-30 | CCCCAGCATTAAGCCTGGCATGTAGTTGCC | CCCCAGCATTAAGCCTGGCATGTAGTTGCCTATGTGCGGCATTGACTAAGAG |
| CDH1-31 | GGGAGGATTAGGCTGCAGGGTTACCCCA | GGGAGGATTAGGCTGCAGGGTTACCCCATATGTGCGGCATTGACTAAGAG |
| CDH1-32 | CAGGAGCCTTGGGAGTGGGTTTACACCTCAA | CAGGAGCCTTGGGAGTGGGTTTACACCTCAATATGTGCGGCATTGACTAAGAG |
| CDH1-33 | AGCAACTCAGTGGTGGAGGAGGGATTCCAGT | AGCAACTCAGTGGTGGAGGAGGGATTCCAGTTATGTGCGGCATTGACTAAGAG |
| CDH1-34 | TTCCCTTCCTTGAGAAAACTGGCCCAGGCT | TTCCCTTCCTTGAGAAAACTGGCCCAGGCTTATGTGCGGCATTGACTAAGAG |
| CDH1-35 | CTGGGCCTGTCTTCCACAAGACATCGCCT | CTGGGCCTGTCTTCCACAAGACATCGCCTTATGTGCGGCATTGACTAAGAG |
| CDH1-36 | GCAGCACATAGAGGGCCCTTAGGGGATGT | GCAGCACATAGAGGGCCCTTAGGGGATGTTATGTGCGGCATTGACTAAGAG |
| CDH1-37 | TTCAGGTGGGTACCAGCCAGTTGACATCCAG | TTCAGGTGGGTACCAGCCAGTTGACATCCAGTATGTGCGGCATTGACTAAGAG |
| CDH1-38 | ACCAGCCTCGCACCTTCAGGGAACGT | ACCAGCCTCGCACCTTCAGGGAACGTTATGTGCGGCATTGACTAAGAG |
| CDH1-39 | AGTGAAACGGGGTAGGGAGTGCCTGGG | AGTGAAACGGGGTAGGGAGTGCCTGGGTATGTGCGGCATTGACTAAGAG |
| CDH1-40 | GTAGTGGTGCAGGGCCCCTTTAGCTGAGG | GTAGTGGTGCAGGGCCCCTTTAGCTGAGGTATGTGCGGCATTGACTAAGAG |
| CDH1-41 | CCATCAGCTTCCACCGAGACGAAGTGATGCA | CCATCAGCTTCCACCGAGACGAAGTGATGCATATGTGCGGCATTGACTAAGAG |
| CDH1-42 | AACCAGACCGTGCAGCCAACTCCTGC | AACCAGACCGTGCAGCCAACTCCTGCTATGTGCGGCATTGACTAAGAG |
| CDH1-43 | AGTCCCAGGCAACTCAGACCTTCCCTGG | AGTCCCAGGCAACTCAGACCTTCCCTGGTATGTGCGGCATTGACTAAGAG |
| CDH1-44 | TGGAGAAGGTGGGTGTTTTGTGTGTTCCCGT | TGGAGAAGGTGGGTGTTTTGTGTGTTCCCGTTATGTGCGGCATTGACTAAGAG |
| CDH1-45 | ATGAGCCACCGTGAATGGCCAGAAGCAC | ATGAGCCACCGTGAATGGCCAGAAGCACTATGTGCGGCATTGACTAAGAG |
| CDH1-46 | TGGTGTTCTTGCCTTTGGTTTGCCTAAGGCC | TGGTGTTCTTGCCTTTGGTTTGCCTAAGGCCTATGTGCGGCATTGACTAAGAG |
| CDH1-47 | CCAGTGATTCCACGTGGTCCTGCCCTATGT | CCAGTGATTCCACGTGGTCCTGCCCTATGTTATGTGCGGCATTGACTAAGAG |
| CDH1-48 | GGTGGCCTTTCAGGTTTTGTCAGGGCCA | GGTGGCCTTTCAGGTTTTGTCAGGGCCATATGTGCGGCATTGACTAAGAG |
| CDH1-49 | TGGTCAGACATCAACACCGCCTGCCTCA | TGGTCAGACATCAACACCGCCTGCCTCATATGTGCGGCATTGACTAAGAG |
| CDH1-50 | GGCTTGACTGAGATGCCCCAGTGGCTTG | GGCTTGACTGAGATGCCCCAGTGGCTTGTATGTGCGGCATTGACTAAGAG |
| CDH1-51 | TGTGTGCTGTTATAGCTACGTGGCCTTGGGC | TGTGTGCTGTTATAGCTACGTGGCCTTGGGCTATGTGCGGCATTGACTAAGAG |
| CDH1-52 | GGGGTGTTGGAGGGAGGCTGGAGAATGA | GGGGTGTTGGAGGGAGGCTGGAGAATGATATGTGCGGCATTGACTAAGAG |
| CDH1-53 | AAGGGAGGCAACTGAGGCCACAGTGTCT | AAGGGAGGCAACTGAGGCCACAGTGTCTTATGTGCGGCATTGACTAAGAG |
| CDH1-54 | CAGCCACCACCCTGGATTGGCTTCCAAC | CAGCCACCACCCTGGATTGGCTTCCAACTATGTGCGGCATTGACTAAGAG |
| CDH1-55 | TCCAGGGTGTTGGCTGAGCAGAGCAAGT | TCCAGGGTGTTGGCTGAGCAGAGCAAGTTATGTGCGGCATTGACTAAGAG |
| CDH1-56 | GAGGCTTGTCATGGCCTGACCGAGGTTGT | GAGGCTTGTCATGGCCTGACCGAGGTTGTTATGTGCGGCATTGACTAAGAG |
| CDH1-57 | TGCCTCCGCTTCATTCTCCACCCTCCT | TGCCTCCGCTTCATTCTCCACCCTCCTTATGTGCGGCATTGACTAAGAG |
| CDH1-58 | CCACACACCCGCAGCACTCATTTTAACCCC | CCACACACCCGCAGCACTCATTTTAACCCCTATGTGCGGCATTGACTAAGAG |
| CDH1-59 | GGGGCGTTCAGGAGGCTACAGCCTAGT | GGGGCGTTCAGGAGGCTACAGCCTAGTTATGTGCGGCATTGACTAAGAG |
| CDH1-60 | CCCCTTCCCTCTCCCATCATGTGTGGGT | CCCCTTCCCTCTCCCATCATGTGTGGGTTATGTGCGGCATTGACTAAGAG |
| CDH1-61 | ACCACTGCTGGAACAGGTGCCCCATCT | ACCACTGCTGGAACAGGTGCCCCATCTTATGTGCGGCATTGACTAAGAG |
| CDH1-62 | GATTACAGGGTTGAGCCTGCACCTGACCCT | GATTACAGGGTTGAGCCTGCACCTGACCCTTATGTGCGGCATTGACTAAGAG |
| CDH1-63 | TGGAATTTCTCTCCCTCCGCAAGCCTAGTGC | TGGAATTTCTCTCCCTCCGCAAGCCTAGTGCTATGTGCGGCATTGACTAAGAG |
| CDH1-64 | GGGGCGAATGTTCCTTCAGAGGCTGACCT | GGGGCGAATGTTCCTTCAGAGGCTGACCTTATGTGCGGCATTGACTAAGAG |
| CDH1-65 | AGGCTGGTCTTGACGGGACTTGGACTCAAG | AGGCTGGTCTTGACGGGACTTGGACTCAAGTATGTGCGGCATTGACTAAGAG |
| CDH1-66 | GGGCCCAGGACTTGTTCTCCCACATCTCA | GGGCCCAGGACTTGTTCTCCCACATCTCATATGTGCGGCATTGACTAAGAG |
| CDH1-67 | GCTGTGTTGTTACGCATGCAGCCACAGCT | GCTGTGTTGTTACGCATGCAGCCACAGCTTATGTGCGGCATTGACTAAGAG |
| CDH1-68 | AACCCCAGCTTCTCCAGTCAAGGCAGCA | AACCCCAGCTTCTCCAGTCAAGGCAGCATATGTGCGGCATTGACTAAGAG |
| CDH1-69 | GGCCCGCAGGAGCAAGAGCTAACCTTTCT | GGCCCGCAGGAGCAAGAGCTAACCTTTCTTATGTGCGGCATTGACTAAGAG |
| CDH1-70 | AGCCAGTACGCCCTGCGACACTGACT | AGCCAGTACGCCCTGCGACACTGACTTATGTGCGGCATTGACTAAGAG |
| CDH1-71 | AAGGGGACTTTGCTCCCTGCTTCCCATCC | AAGGGGACTTTGCTCCCTGCTTCCCATCCTATGTGCGGCATTGACTAAGAG |
| CDH1-72 | TGGTGAGTCCTGGCGGCTAAGTAGCATCAGA | TGGTGAGTCCTGGCGGCTAAGTAGCATCAGATATGTGCGGCATTGACTAAGAG |
| CDH1-73 | CCTTTGTATGCAGGGTGGGCAAAACACTGCT | CCTTTGTATGCAGGGTGGGCAAAACACTGCTTATGTGCGGCATTGACTAAGAG |
| CDH1-74 | CTTGCCAGCGTGACAGTGAGCTTCCCAG | CTTGCCAGCGTGACAGTGAGCTTCCCAGTATGTGCGGCATTGACTAAGAG |
| CDH1-75 | ATTTGCTAAGGCCACCCAGCAAGAACATGGC | ATTTGCTAAGGCCACCCAGCAAGAACATGGCTATGTGCGGCATTGACTAAGAG |
| CDH1-76 | TTTTCTCTGGCAAGGGCAGGGCTATTGGCT | TTTTCTCTGGCAAGGGCAGGGCTATTGGCTTATGTGCGGCATTGACTAAGAG |
| CDH1-77 | GGGGACAAATCAGCCCAAGTTGAGGGCTCT | GGGGACAAATCAGCCCAAGTTGAGGGCTCTTATGTGCGGCATTGACTAAGAG |
| CDH1-78 | TGGATGGATGTCTGCCTGGGTTCTTGGCA | TGGATGGATGTCTGCCTGGGTTCTTGGCATATGTGCGGCATTGACTAAGAG |
| CDH1-79 | AAAACTCAGCACAGTGAGTCCTTGCCAGGCA | AAAACTCAGCACAGTGAGTCCTTGCCAGGCATATGTGCGGCATTGACTAAGAG |
| CDH1-80 | TTTTGCTCCGCCATCCTCAGCACATGACCT | TTTTGCTCCGCCATCCTCAGCACATGACCTTATGTGCGGCATTGACTAAGAG |
| CDH1-81 | TGGGGACGCTGTCTGGCTAGGTTGGA | TGGGGACGCTGTCTGGCTAGGTTGGATATGTGCGGCATTGACTAAGAG |
| CDH1-82 | CCATGTCCCCTCCTTTATCCCTCAGGGCAG | CCATGTCCCCTCCTTTATCCCTCAGGGCAGTATGTGCGGCATTGACTAAGAG |
| CDH1-83 | TCACCACTGGGCTGGACCGAGAGGTC | TCACCACTGGGCTGGACCGAGAGGTCTATGTGCGGCATTGACTAAGAG |
| CDH1-84 | CTACCGAACCCAGCGACATCTGAAGAGCCC | CTACCGAACCCAGCGACATCTGAAGAGCCCTATGTGCGGCATTGACTAAGAG |
| CDH1-85 | GCTGCTGCCTCAGTGGACTCCAGGAGA | GCTGCTGCCTCAGTGGACTCCAGGAGATATGTGCGGCATTGACTAAGAG |
| CDH1-86 | CCCCTGTCTGGTATGAGGGGTGCTCTGTG | CCCCTGTCTGGTATGAGGGGTGCTCTGTGTATGTGCGGCATTGACTAAGAG |
| CDH1-87 | CCCCAGAGGATGACACCCGGGACAACG | CCCCAGAGGATGACACCCGGGACAACGTATGTGCGGCATTGACTAAGAG |
| CDH1-88 | ACTCGTAACGACGTTGCACCAACCCTCATGA | ACTCGTAACGACGTTGCACCAACCCTCATGATATGTGCGGCATTGACTAAGAG |
| CDH1-89 | ATGGTCCAGTGGCCCTCGGTGAGTCTTC | ATGGTCCAGTGGCCCTCGGTGAGTCTTCTATGTGCGGCATTGACTAAGAG |
| CDH1-90 | TGGCAGCCCCACTCTGATCTATGGGGAC | TGGCAGCCCCACTCTGATCTATGGGGACTATGTGCGGCATTGACTAAGAG |
| CDH1-91 | GGTGTGCCACAAGTCTGGGTGCATTGTCG | GGTGTGCCACAAGTCTGGGTGCATTGTCGTATGTGCGGCATTGACTAAGAG |
| CDH1-92 | CGAGGACGACTAGGGGACTCGAGAGAGGC | CGAGGACGACTAGGGGACTCGAGAGAGGCTATGTGCGGCATTGACTAAGAG |
| CDH1-93 | TGGCCTCAGTCAAAACGTGGAGAAAGAGGCC | TGGCCTCAGTCAAAACGTGGAGAAAGAGGCCTATGTGCGGCATTGACTAAGAG |
| CDH1--94 | AGGAGTATTCCCGTTTTACAGTTGAGCAAACCGAGGC | AGGAGTATTCCCGTTTTACAGTTGAGCAAACCGAGGCTATGTGCGGCATTGACTAAGAG |
| CDH1--95 | TAAGAGAGTGACGTCCACTTGCTCAGGGTCAGC | TAAGAGAGTGACGTCCACTTGCTCAGGGTCAGCTATGTGCGGCATTGACTAAGAG |
| CDH1--96 | AGAGCAGATATCTGAACTGGGATCTGACTCCCAGGC | AGAGCAGATATCTGAACTGGGATCTGACTCCCAGGCTATGTGCGGCATTGACTAAGAG |
| CDH1--97 | CCCATTAAGCACCATCTCTGGGATGACTGAAGAGCA | CCCATTAAGCACCATCTCTGGGATGACTGAAGAGCATATGTGCGGCATTGACTAAGAG |
| CDH1--98 | CTGAGAGACTGTGCCTGGACCAAAACATCTTGATACAGG | CTGAGAGACTGTGCCTGGACCAAAACATCTTGATACAGGTATGTGCGGCATTGACTAAGAG |
| CDH1--99 | AGCAAGGGAAGGGGCTAGTTTCTCTTCTGGTTTCA | AGCAAGGGAAGGGGCTAGTTTCTCTTCTGGTTTCATATGTGCGGCATTGACTAAGAG |
| CDH1--100 | TTGGATATTTTGATGTCAGTGGGCATTGAGGAGTGGC | TTGGATATTTTGATGTCAGTGGGCATTGAGGAGTGGCTATGTGCGGCATTGACTAAGAG |
| CDH1--101 | TTCTCGGGGAAGGCAAACTTGATCCAGTCTTGG | TTCTCGGGGAAGGCAAACTTGATCCAGTCTTGGTATGTGCGGCATTGACTAAGAG |
| CDH1--102 | GTGCTAAGGCTGGTGACTTTGTTTTCCTGCACC | GTGCTAAGGCTGGTGACTTTGTTTTCCTGCACCTATGTGCGGCATTGACTAAGAG |
| CDH1--103 | AAGATATTTGGTAGCACCAAGGGTGGGTTGGTCTGG | AAGATATTTGGTAGCACCAAGGGTGGGTTGGTCTGGTATGTGCGGCATTGACTAAGAG |
| CDH1--104 | AATAGTGACACGTATTCACTTTTGCCTCAGACTCTGGGG | AATAGTGACACGTATTCACTTTTGCCTCAGACTCTGGGGTATGTGCGGCATTGACTAAGAG |
| CDH1--105 | CCTCTGGTGTGGTTGGTTTCAGTTCCCTTTATGTTCAC | CCTCTGGTGTGGTTGGTTTCAGTTCCCTTTATGTTCACTATGTGCGGCATTGACTAAGAG |
| CDH1--106 | TTTTGGACTGTGGGGTCTCCTTGTATCAGTCCAATGT | TTTTGGACTGTGGGGTCTCCTTGTATCAGTCCAATGTTATGTGCGGCATTGACTAAGAG |
| CDH1--107 | GATTTTAAAAGGCCGCTAGGACTTGGGATGTGGCC | GATTTTAAAAGGCCGCTAGGACTTGGGATGTGGCCTATGTGCGGCATTGACTAAGAG |
| CDH1--108 | TGACTCTCTTTGGACAGTGCACGGAAGTGAAGGG | TGACTCTCTTTGGACAGTGCACGGAAGTGAAGGGTATGTGCGGCATTGACTAAGAG |
| CDH1--109 | ATGGATGTAACATTGGCACCAGGTTTAGACATTGGCTTC | ATGGATGTAACATTGGCACCAGGTTTAGACATTGGCTTCTATGTGCGGCATTGACTAAGAG |
| CDH1--110 | TTTTGTGCACAGGCAGCTGTAGGGTCTTAACTCAG | TTTTGTGCACAGGCAGCTGTAGGGTCTTAACTCAGTATGTGCGGCATTGACTAAGAG |
| CDH1--111 | GGGACTTTGGAGTGTTTGCTAGGGTCATTCCTCTGC | GGGACTTTGGAGTGTTTGCTAGGGTCATTCCTCTGCTATGTGCGGCATTGACTAAGAG |
| CDH1--112 | ATGCCTGGCTAATAACCCAGTCCTTTAGAGTTGCTGC | ATGCCTGGCTAATAACCCAGTCCTTTAGAGTTGCTGCTATGTGCGGCATTGACTAAGAG |
| CDH1--113 | AAAAGAAAAGCTGGCAGGCTTTCCCAGTGATTGTTTTCT | AAAAGAAAAGCTGGCAGGCTTTCCCAGTGATTGTTTTCTTATGTGCGGCATTGACTAAGAG |
| CDH1--114 | AAACACAGGGCCTATATTTCAGTGTTGAGGTTTGGCTG | AAACACAGGGCCTATATTTCAGTGTTGAGGTTTGGCTGTATGTGCGGCATTGACTAAGAG |
| CDH1--115 | TGAAGGATGTGCTGAACGTTTCAGAAAACAGTATGCAGC | TGAAGGATGTGCTGAACGTTTCAGAAAACAGTATGCAGCTATGTGCGGCATTGACTAAGAG |
| CDH1--116 | AAAAGCAAACCCAGAAACCAGCTTTGGGATCACCT | AAAAGCAAACCCAGAAACCAGCTTTGGGATCACCTTATGTGCGGCATTGACTAAGAG |
| CDH1--117 | CCCCTACCCCTCAAACTCATAAAAGTCACTTCCCCA | CCCCTACCCCTCAAACTCATAAAAGTCACTTCCCCATATGTGCGGCATTGACTAAGAG |
| CDH1--118 | ACTCATGTCTGTGACAAAGGTTGATCTGAGCTTACTGGG | ACTCATGTCTGTGACAAAGGTTGATCTGAGCTTACTGGGTATGTGCGGCATTGACTAAGAG |
| CDH1--119 | GGTTATGGAAATCCCTGTTTGCTCTCCAGGGCT | GGTTATGGAAATCCCTGTTTGCTCTCCAGGGCTTATGTGCGGCATTGACTAAGAG |
| CDH1--120 | GTCTGTTACACATTCCCTTCCAAGGTAGCCCCAGA | GTCTGTTACACATTCCCTTCCAAGGTAGCCCCAGATATGTGCGGCATTGACTAAGAG |
| CDH1--121 | AGAAGCTGCCCAAACCCTAAAATCCCCGAGAG | AGAAGCTGCCCAAACCCTAAAATCCCCGAGAGTATGTGCGGCATTGACTAAGAG |
| CDH1--122 | TATCCTGCCTCAGCTCTATCTCCTTTGAGGGCAC | TATCCTGCCTCAGCTCTATCTCCTTTGAGGGCACTATGTGCGGCATTGACTAAGAG |
| CDH1--123 | CGGAACATACATGCCAATGGGCAGGAATGTAAAGAGAG | CGGAACATACATGCCAATGGGCAGGAATGTAAAGAGAGTATGTGCGGCATTGACTAAGAG |
| CDH1--124 | TTTTCCCTCTCAAACTCTATGGACTTGGTCCAGCCC | TTTTCCCTCTCAAACTCTATGGACTTGGTCCAGCCCTATGTGCGGCATTGACTAAGAG |
| CDH1--125 | TTAAGGTTGATTGTAAATGGGCGGGGAAGAATTTGGTGG | TTAAGGTTGATTGTAAATGGGCGGGGAAGAATTTGGTGGTATGTGCGGCATTGACTAAGAG |
| CDH1--126 | GTTAGAACCTTCCATTATGCTCTGTGTGTGCTGGGG | GTTAGAACCTTCCATTATGCTCTGTGTGTGCTGGGGTATGTGCGGCATTGACTAAGAG |
| CDH1--127 | ATACTGTGTTCATAGCTTCCCTCTGAACAGCTGTGCA | ATACTGTGTTCATAGCTTCCCTCTGAACAGCTGTGCATATGTGCGGCATTGACTAAGAG |
| CDH1--128 | CTCCAATCCTCCACTTCTGCCCAAGATTTGGGT | CTCCAATCCTCCACTTCTGCCCAAGATTTGGGTTATGTGCGGCATTGACTAAGAG |
| CDH1--129 | AGCATGGAGACATCATAGGATGGTATGTCAAGCGTTCA | AGCATGGAGACATCATAGGATGGTATGTCAAGCGTTCATATGTGCGGCATTGACTAAGAG |
| CDH1--130 | AAACCAGTTTCTTCACCCTGCCTGCTTCTGTGT | AAACCAGTTTCTTCACCCTGCCTGCTTCTGTGTTATGTGCGGCATTGACTAAGAG |
| CDH1--131 | TAAGCAAACAGTGCACTGCAAACAGTGACTAAGCCT | TAAGCAAACAGTGCACTGCAAACAGTGACTAAGCCTTATGTGCGGCATTGACTAAGAG |
| CDH1--132 | AAAATCAAATCACCTAGGGCACTTCATGCAGACTTGCC | AAAATCAAATCACCTAGGGCACTTCATGCAGACTTGCCTATGTGCGGCATTGACTAAGAG |
| CDH1--133 | CTCTTGCAGATTCACTGAGAGCCAATCTCCAAGGGT | CTCTTGCAGATTCACTGAGAGCCAATCTCCAAGGGTTATGTGCGGCATTGACTAAGAG |
| CDH1--134 | ATTGAGAACTCTATGGATGCAAGGAGGGGTGGATGG | ATTGAGAACTCTATGGATGCAAGGAGGGGTGGATGGTATGTGCGGCATTGACTAAGAG |
| CDH1--135 | CCCCTACACTCAGGGAGATTCCAGAAAATCTCCAAGAGA | CCCCTACACTCAGGGAGATTCCAGAAAATCTCCAAGAGATATGTGCGGCATTGACTAAGAG |
| CDH1--136 | GACCTCTCAGCAGGTAATTCCAGTAGGCTCCAAGA | GACCTCTCAGCAGGTAATTCCAGTAGGCTCCAAGATATGTGCGGCATTGACTAAGAG |
| CDH1--137 | CTTGATGAGTAAGAGGGTGTCTTCCATGCAAGGAAAGGG | CTTGATGAGTAAGAGGGTGTCTTCCATGCAAGGAAAGGGTATGTGCGGCATTGACTAAGAG |
| CDH1--138 | GTGGGTCATGGAGGGTTTTCTCTTTCTGGGCT | GTGGGTCATGGAGGGTTTTCTCTTTCTGGGCTTATGTGCGGCATTGACTAAGAG |
| CDH1--139 | CACATCAGCGTTTAGAGACAGTCGGTTCCCACTG | CACATCAGCGTTTAGAGACAGTCGGTTCCCACTGTATGTGCGGCATTGACTAAGAG |
| CDH1--140 | TCCCAGTGGAGGTTTTGAGAAAGAGGTTGGGAGA | TCCCAGTGGAGGTTTTGAGAAAGAGGTTGGGAGATATGTGCGGCATTGACTAAGAG |
| CDH1--141 | ATGTTCCTTTGGACCAACACTGGAAACAGAGGAGAAAGA | ATGTTCCTTTGGACCAACACTGGAAACAGAGGAGAAAGATATGTGCGGCATTGACTAAGAG |
| CDH1--142 | TTTCAAAGGGGTATTTGGGAATGTCAGCTCTGTGCTTG | TTTCAAAGGGGTATTTGGGAATGTCAGCTCTGTGCTTGTATGTGCGGCATTGACTAAGAG |
| CDH1--143 | CGCTTGACAGTTAATCCTCCCTGTAGTCCAAGGTGG | CGCTTGACAGTTAATCCTCCCTGTAGTCCAAGGTGGTATGTGCGGCATTGACTAAGAG |
| CDH1--144 | AAAACCAGGTCTCTCATCCTGTTCCCTCTACTACACTGT | AAAACCAGGTCTCTCATCCTGTTCCCTCTACTACACTGTTATGTGCGGCATTGACTAAGAG |
| CDH1--145 | CAGTTTCAGCCGGTTTCCCTCTTGACCTTCACT | CAGTTTCAGCCGGTTTCCCTCTTGACCTTCACTTATGTGCGGCATTGACTAAGAG |
| CDH1--146 | TTCAAGACTTGGGAGTGTAGGGACAGGGAAACCG | TTCAAGACTTGGGAGTGTAGGGACAGGGAAACCGTATGTGCGGCATTGACTAAGAG |
| CDH1--147 | GCTCTTGCTGCTGAAAGAGGGAAAGTTACTACTGGGA | GCTCTTGCTGCTGAAAGAGGGAAAGTTACTACTGGGATATGTGCGGCATTGACTAAGAG |
| CDH1--148 | TGTTCCACATGAGCAAGATCATAAACGGTGGCCA | TGTTCCACATGAGCAAGATCATAAACGGTGGCCATATGTGCGGCATTGACTAAGAG |
| CDH1--149 | GATTCTGTGGTTTGGCATTCAGGTACGCCTGTACA | GATTCTGTGGTTTGGCATTCAGGTACGCCTGTACATATGTGCGGCATTGACTAAGAG |
| CDH1--150 | CATGACGCCCAGCTGTTTCTCTATTCCCAAAAGAGAAG | CATGACGCCCAGCTGTTTCTCTATTCCCAAAAGAGAAGTATGTGCGGCATTGACTAAGAG |
| CDH1--151 | TTTCAGCATGTCTTCACCTTCATTGTCGCAGCAGC | TTTCAGCATGTCTTCACCTTCATTGTCGCAGCAGCTATGTGCGGCATTGACTAAGAG |
| CDH1--152 | TTCCTTCCTGGTCTCCCCACTTATTCCTTCCCC | TTCCTTCCTGGTCTCCCCACTTATTCCTTCCCCTATGTGCGGCATTGACTAAGAG |
| CDH1--153 | TTCCTCCAAGCCAGTCTTTTCTCCATCCATCAGTCA | TTCCTCCAAGCCAGTCTTTTCTCCATCCATCAGTCATATGTGCGGCATTGACTAAGAG |
| CDH1--154 | GATCTGTCCTTTGACTCATGTACCTGGGGTCCTCA | GATCTGTCCTTTGACTCATGTACCTGGGGTCCTCATATGTGCGGCATTGACTAAGAG |
| CDH1--155 | TCTCTTCCCACTCTTCTGCATGCTCACTTTGCC | TCTCTTCCCACTCTTCTGCATGCTCACTTTGCCTATGTGCGGCATTGACTAAGAG |
| CDH1--156 | AGCTCACTCTTTTCTGTCCTGAAAAGACTGCAAGCC | AGCTCACTCTTTTCTGTCCTGAAAAGACTGCAAGCCTATGTGCGGCATTGACTAAGAG |
| CDH1--157 | CCCCTATTTATGATAGTAAACCCTGCCCTCAGCCCT | CCCCTATTTATGATAGTAAACCCTGCCCTCAGCCCTTATGTGCGGCATTGACTAAGAG |
| CDH1--158 | AAAGACAGGGCTTTATGTATTAGCCACAGAGAAGGGAGG | AAAGACAGGGCTTTATGTATTAGCCACAGAGAAGGGAGGTATGTGCGGCATTGACTAAGAG |
| CDH1--159 | CATTTCTACCCCACACTTCTGAGTGTAAGGAAGCACCA | CATTTCTACCCCACACTTCTGAGTGTAAGGAAGCACCATATGTGCGGCATTGACTAAGAG |
| CDH1--160 | GATCAAAATCTAGGCAGATATCTGAGCCCGCCAGAGC | GATCAAAATCTAGGCAGATATCTGAGCCCGCCAGAGCTATGTGCGGCATTGACTAAGAG |
| CDH1--161 | CCCTGGGTATCTCAGCATCCTTCAGACCCATGA | CCCTGGGTATCTCAGCATCCTTCAGACCCATGATATGTGCGGCATTGACTAAGAG |
| CDH1--162 | AAAAGCAAGGCCTGCTTAATCAGATCTCGGCCA | AAAAGCAAGGCCTGCTTAATCAGATCTCGGCCATATGTGCGGCATTGACTAAGAG |
| CDH1--163 | TATGTTGAACTCTTCCCGTATGCCATCTCCTGTAACCC | TATGTTGAACTCTTCCCGTATGCCATCTCCTGTAACCCTATGTGCGGCATTGACTAAGAG |
| CDH1--164 | CAGGTGAAAGGGATGGATCATTCCTTCTCTGAAGGTCT | CAGGTGAAAGGGATGGATCATTCCTTCTCTGAAGGTCTTATGTGCGGCATTGACTAAGAG |
| CDH1--165 | AAAACTATGCATTGGGGTGGAGAAATTTAGGGGCAGTC | AAAACTATGCATTGGGGTGGAGAAATTTAGGGGCAGTCTATGTGCGGCATTGACTAAGAG |
| CDH1--166 | ATAGATGTTAGAACTGGACTGTGTCAGCTGGGTGTGG | ATAGATGTTAGAACTGGACTGTGTCAGCTGGGTGTGGTATGTGCGGCATTGACTAAGAG |
| CDH1--167 | GCCAGGCCAGGCATTTTCTAGAGAAGGAATCCTG | GCCAGGCCAGGCATTTTCTAGAGAAGGAATCCTGTATGTGCGGCATTGACTAAGAG |
| CDH1--168 | CACCCCTCAGCATGTTGCCCAAGAGGAATATTGC | CACCCCTCAGCATGTTGCCCAAGAGGAATATTGCTATGTGCGGCATTGACTAAGAG |
| CDH1--169 | TTGGAATCTCACCTTCAGTACGAGGCTTCCTCCC | TTGGAATCTCACCTTCAGTACGAGGCTTCCTCCCTATGTGCGGCATTGACTAAGAG |
| CDH1--170 | AAAAGGGAACAAGATGACCAACTTGTTGGTGGGGA | AAAAGGGAACAAGATGACCAACTTGTTGGTGGGGATATGTGCGGCATTGACTAAGAG |
| CDH1--171 | CTGGGCACTTACTTTATGAACAGGTGGGGTGGG | CTGGGCACTTACTTTATGAACAGGTGGGGTGGGTATGTGCGGCATTGACTAAGAG |
| CDH1--172 | ATAGTGACAGGTACTCTAGGTGCCGGCTCTTACTCA | ATAGTGACAGGTACTCTAGGTGCCGGCTCTTACTCATATGTGCGGCATTGACTAAGAG |
| CDH1--173 | TAAGTCCCAGGAGATACTAGCCCTAGATCTCGTCTCTGT | TAAGTCCCAGGAGATACTAGCCCTAGATCTCGTCTCTGTTATGTGCGGCATTGACTAAGAG |
| CDH1--174 | AGCTTCGTGCTAAAAGGTTTTGGAGAAATAACAGCTGCT | AGCTTCGTGCTAAAAGGTTTTGGAGAAATAACAGCTGCTTATGTGCGGCATTGACTAAGAG |
| CDH1--175 | TGGTGACGTTCATGTGGCTGGTTTCATTACCACC | TGGTGACGTTCATGTGGCTGGTTTCATTACCACCTATGTGCGGCATTGACTAAGAG |
| CDH1--176 | CTCACCTGTACAAAGGGGATAAACATAGCCCCTACCAC | CTCACCTGTACAAAGGGGATAAACATAGCCCCTACCACTATGTGCGGCATTGACTAAGAG |
| CDH1--177 | AGTGCATGCTGATTGTGCCCGTGAATAGTTACTGC | AGTGCATGCTGATTGTGCCCGTGAATAGTTACTGCTATGTGCGGCATTGACTAAGAG |
| CDH1--178 | ATCCTTCCTGTGCTCAGCGGCTCTATTTCTAAGAATGT | ATCCTTCCTGTGCTCAGCGGCTCTATTTCTAAGAATGTTATGTGCGGCATTGACTAAGAG |
| CDH1--179 | TTCCATTCTAGATCCATCACTTTGGCCTAGAAAGGAGCC | TTCCATTCTAGATCCATCACTTTGGCCTAGAAAGGAGCCTATGTGCGGCATTGACTAAGAG |
| CDH1--180 | GCTCTGTGTCAGTTACCAGCCTTGTTTCCAGGAG | GCTCTGTGTCAGTTACCAGCCTTGTTTCCAGGAGTATGTGCGGCATTGACTAAGAG |
| CDH1--181 | TTTGGAGCTGACCCAGGGGTGATAGAAAACACTGG | TTTGGAGCTGACCCAGGGGTGATAGAAAACACTGGTATGTGCGGCATTGACTAAGAG |
| CDH1--182 | TGTGAGAAAACTCCCTCGTTGGTTCTTCCTACAGGG | TGTGAGAAAACTCCCTCGTTGGTTCTTCCTACAGGGTATGTGCGGCATTGACTAAGAG |
| CDH1--183 | AAGGCTAGCAAGCTGGGAGAACTTGGAGAACC | AAGGCTAGCAAGCTGGGAGAACTTGGAGAACCTATGTGCGGCATTGACTAAGAG |
| CDH1--184 | GCCTGAGTGGTCTAGGCTGATACATCAGGCTTGG | GCCTGAGTGGTCTAGGCTGATACATCAGGCTTGGTATGTGCGGCATTGACTAAGAG |
| CDH1--185 | CCCCACATGTTTATCCCAAATCCCCTTCTCCTCAAGC | CCCCACATGTTTATCCCAAATCCCCTTCTCCTCAAGCTATGTGCGGCATTGACTAAGAG |
| CDH1--186 | TTGCTTCTAGGTGCCCATCTCACCCATCCTACC | TTGCTTCTAGGTGCCCATCTCACCCATCCTACCTATGTGCGGCATTGACTAAGAG |
| CDH1--187 | AGGAAAGATGAATCGGAATGGAGCATTGCTGGGAAG | AGGAAAGATGAATCGGAATGGAGCATTGCTGGGAAGTATGTGCGGCATTGACTAAGAG |
| CDH1--188 | TACTAGGGTGATCTGAGAAGGCCTCACTGAAGGGG | TACTAGGGTGATCTGAGAAGGCCTCACTGAAGGGGTATGTGCGGCATTGACTAAGAG |
| CDH1--189 | CCATGTGACTAGGTAGGGAAGGGTGTTCTAGACGG | CCATGTGACTAGGTAGGGAAGGGTGTTCTAGACGGTATGTGCGGCATTGACTAAGAG |
| CDH1--190 | GCAAAGACACACCCCTGACACTGAGCAGAAGG | GCAAAGACACACCCCTGACACTGAGCAGAAGGTATGTGCGGCATTGACTAAGAG |
| CDH1--191 | GTACCCATCACCAGGACTTTGTCTTTCACTTGGGACT | GTACCCATCACCAGGACTTTGTCTTTCACTTGGGACTTATGTGCGGCATTGACTAAGAG |
| CDH1--192 | GCCATCTTCGTTGTTGCTACCTCTAGGAAGCCTTCT | GCCATCTTCGTTGTTGCTACCTCTAGGAAGCCTTCTTATGTGCGGCATTGACTAAGAG |
| CDH1--193 | GATACGTCCTACTCCTTCCCTTTGCCAGTTGGGA | GATACGTCCTACTCCTTCCCTTTGCCAGTTGGGATATGTGCGGCATTGACTAAGAG |
| CDH1--194 | ACTCAGTGAGTAGTTTGATGAGGGGCTCTTTGCTGT | ACTCAGTGAGTAGTTTGATGAGGGGCTCTTTGCTGTTATGTGCGGCATTGACTAAGAG |
| CDH1--195 | CCCCAGTGGAGGTGACTTATGTCCATCTTTTGGGG | CCCCAGTGGAGGTGACTTATGTCCATCTTTTGGGGTATGTGCGGCATTGACTAAGAG |
| CDH1--196 | ACTTTTGCAGTGAGGGAGTGCATTATAATGGAGAGGAGG | ACTTTTGCAGTGAGGGAGTGCATTATAATGGAGAGGAGGTATGTGCGGCATTGACTAAGAG |
| CDH1--197 | AAAAGCCCCAAACCAGCTGAGCTTAGATGGATCTC | AAAAGCCCCAAACCAGCTGAGCTTAGATGGATCTCTATGTGCGGCATTGACTAAGAG |
| CDH1--198 | GTGCCAGAAGAAAATCCCCACTCCCTACAATGTGTG | GTGCCAGAAGAAAATCCCCACTCCCTACAATGTGTGTATGTGCGGCATTGACTAAGAG |
| CDH1--199 | GGGGAAGAAGTCCAATTTCAGCTGGGAAAATGGGA | GGGGAAGAAGTCCAATTTCAGCTGGGAAAATGGGATATGTGCGGCATTGACTAAGAG |
| CDH1_546 | /5Alex546N/CTCTTAGTCAATGCCGCACA |  |

## References

1. Zhao, Y. *et al.* Nanoscale imaging of clinical specimens using pathology-optimized expansion microscopy. *Nat. Biotechnol.* **35**, 757–764 (2017).

2. Tillberg, P. W. *et al.* Protein-retention expansion microscopy of cells and tissues labeled using standard fluorescent proteins and antibodies. *Nat. Biotechnol.* **34**, 987–992 (2016).

3. Bucur, O. *et al.* Nanoscale imaging of clinical specimens using conventional and rapid-expansion pathology. *Nat. Protoc.* **15**, 1649–1672 (2020).

4. Ku, T. *et al.* Multiplexed and scalable super-resolution imaging of three-dimensional protein localization in size-adjustable tissues. *Nat Biotech* **34**, 973–981 (2016).

5. Susaki, E. A. *et al.* Whole-brain imaging with single-cell resolution using chemical cocktails and computational analysis. *Cell* **157**, 726–739 (2014).

6. Truckenbrodt, S. *et al.* X10 expansion microscopy enables 25‐nm resolution on conventional microscopes. *EMBO Rep.* **19**, e45836 (2018).

7. Damstra, H. G. J. *et al.* Visualizing cellular and tissue ultrastructure using Ten-fold Robust Expansion Microscopy (TREx). *bioRxiv* 2021.02.03.428837 (2021). doi:10.1101/2021.02.03.428837

8. Ijdo, J. W., Wells, R. A., Baldini, A. & Reeders, S. T. Improved telomere detection using a telomere repeat probe (TTAGGG)n generated by PCR. *Nucleic Acids Res.* **19**, 4780 (1991).

9. Suntronpong, A. *et al.* CENP-B box, a nucleotide motif involved in centromere formation, occurs in a New World monkey. *Biol. Lett.* **12**, (2016).

10. Dertinger, T. *et al.* Fast, background-free, 3D super-resolution optical fluctuation imaging (SOFI). *Proc. Natl. Acad. Sci. U. S. A.* **106**, 22287–22292 (2009).

11. Dertinger, T., Xu, J., Naini, O. F., Vogel, R. & Weiss, S. SOFI-based 3D superresolution sectioning with a widefield microscope. *Opt. Nanoscopy* **1**, 1–5 (2012).

12. Moser, F. *et al.* Cryo-SOFI enabling low-dose super-resolution correlative light and electron cryo-microscopy. *Proc. Natl. Acad. Sci. U. S. A.* **116**, 4804–4809 (2019).

13. Wijesekara, P. *et al.* Engineering rotating apical-out airway organoid for assessing respiratory cilia motility. *iScience* **25**, (2022)*.* doi: 10.1016/j.isci.2022.104730

14. Hua, K. & Ferland, R. J. Fixation methods can differentially affect ciliary protein immunolabeling. *Cilia* **6**, 5 (2017).

15. Halpern, A. R., Howard, M. D. & Vaughan, J. C. Point by Point: An Introductory Guide to Sample Preparation for Single-Molecule, Super-Resolution Fluorescence Microscopy. *Curr. Protoc. Chem. Biol.* **7**, 103–120 (2015).

16. Ponjavić, I., Vukušić, K. & Tolić, I. M. Expansion microscopy of the mitotic spindle. *Methods Cell Biol.* **161**, 247–274 (2021).

17. Whelan, D. R. & Bell, T. D. M. Image artifacts in single molecule localization microscopy: Why optimization of sample preparation protocols matters. *Sci. Rep.* **5**, 1–10 (2015).
