# Supplementary material for: Magnify is a universal molecular anchoring strategy for expansion microscopy
Source: Nat Biotechnol. 2023 Jan 2;41(6):858–69. doi: 10.1038/s41587-022-01546-1 (PMC10264239; doi:10.1038/s41587-022-01546-1)
Supplement: Supplementary file 1 [file 41587_2022_1546_MOESM1_ESM.pdf]

nature biotechnology

Article https://doi.org/10.1038/s41587-022-01546-1

Magnify is a universal molecular anchoring strategy for expansion microscopy

In the format provided by the authors and unedited

Magnify: A versatile 11-fold expansion microscopy technique with universal molecular retention

Authors
Aleksandra Klimas[1±], Brendan R. Gallagher[1±], Piyumi Wijesekara[2], Sinda Fekir[3,4], Emma F. DiBernardo[1], Zhangyu Cheng[1], Donna B. Stolz[5], Franca Cambi[6,7], Simon Watkins[5], Steven L Brody[8], Amjad Horani[9,10], Alison L. Barth[1], Christopher I. Moore[3,4], Xi Ren[2], Yongxin Zhao[1+]

Affiliations
[1]Department of Biological Sciences, Carnegie Mellon University, Pittsburgh, Pennsylvania, USA. [2]Department of Biomedical Engineering, Carnegie Mellon University, Pittsburgh, Pennsylvania, USA. [3]Department of Neuroscience, Brown University, Providence, Rhode Island, USA. [4]Carney Institute for Brain Science, Brown University, Providence, Rhode Island, USA. [5]Department of Cell Biology, Center for Biologic Imaging, University of Pittsburgh, Pittsburgh, Pennsylvania. [6]Veterans Administration Pittsburgh, Pittsburgh, Pennsylvania. [7]Department of Neurology/PIND, University of Pittsburgh, Pittsburgh, Pennsylvania, USA. [8]Department of Medicine, Washington University School of Medicine, St. Louis, Missouri, United States. [9]Department of Pediatrics, Washington University School of Medicine, St. Louis, Missouri, United States. [10]Department of Cell Biology and Physiology, Washington University School of Medicine, St. Louis, Missouri, United States

±These authors contributed equally to this work
Aleksandra Klimas, Brendan R. Gallagher

[+]Correspondence to yongxinz@andrew.cmu.edu

Contents

Supplementary Notes 4
Supplementary Note 1: Exploration of Anchoring and Homogenization Buffers 4
Supplementary Note 2: Exploration of Gel Chemistry 5
Supplementary Note 3: DNA FISH experiment on tissues homogenized in MAGNIFY gel 6
Supplementary Note 4: Use of SOFI with MAGNIFY 6
Supplementary Note 5: Parameter free resolution estimation of MAGNIFY-SOFI 6
Supplementary Note 6: High-quality imaging of tubulin ultrastructure in cell culture. 7
Supplementary Figures 9
Supplementary Figure 1: The full workflow for MAGNIFY. 9
Supplementary Figure 2: Comparison of anchoring, homogenization, and gelling strategies. 11
Supplementary Figure 3: Examples of Post Expansion Staining with MAGNIFY 12

Supplementary Figure 4: Exploration and characterization of MAGNIFY gel chemistry... 14
Supplementary Figure 5: Overview of SOFI ... 17
Supplementary Figure 6: Example images of tubulin structure in cell culture and cilia in human lung organoids. ... 18
Supplementary Figure 7: Example images of MAGNIFY expansion of FFPE tissue. ... 20
Supplementary Figure 8: DNA FISH with MAGNIFY using FFPE human tissue. ... 21
Supplementary Videos ... 23
Supplementary Video 1 ... 23
Supplementary Video 2 ... 23
Supplementary Video 3 ... 23
Supplementary Video 4 ... 23
Supplementary Video 5 ... 23
Supplementary Video 6 ... 23
Supplementary Video 7 ... 23
Supplementary Video 8 ... 23
Supplementary Video 9 ... 23
Supplementary Tables ... 24
Supplementary Table 1: Condition optimization for different tissue types ... 24
Supplementary Table 2: Protein Retention for tissues under different anchoring and homogenization conditions. ... 25
Supplementary Table 3: Lipid retention in mouse brain under different anchoring and homogenization conditions. ... 26
Supplementary Table 4: Validated Primary Antibodies and fluorescent labels for Pre and Post MAGNIFY Staining ... 26
Supplementary Table 5: Secondary antibodies used for pre- and post-MAGNIFY staining. ... 27
Supplementary Table 6: Comparison of expansion factors for different hydrogel chemistries for different tissue types ... 28
Supplementary Table 7: Exploration of different hydrogel chemistries and their respective expansion factors of blank gels in water. ... 28
Supplementary Table 8: Gelling Conditions for MAGNIFY Gel. ... 29
Supplementary Table 9: Deformation testing of different gel chemistries ... 29
Supplementary Table 10: Gelling Solution Recipe ... 30
Supplementary Table 11: List of Chemicals and Reagents ... 31
Supplementary Table 12: DNA FISH probes design ... 32
References ... 45

Supplementary Notes

Supplementary Note 1: Exploration of Anchoring and Homogenization Buffers

We gelled deparaffinized and antibody labeled FFPE kidney pieces with the MANGIFY monomer solution containing 0.05% (v/v) methacrolein (**Supplementary Fig. 2a**) allowing the samples to still be homogenized by proteinase K (ProK) digestion, which is typically used to ensure isotropic expansion in most ExM protocols.[1,2] To quantify protein retention (**Fig. 1e,f**; **Supplementary Fig. 2f**; **Supplementary Table 2**), we compared average fluorescence of N-Hydroxysuccinimide (NHS) ester-dye conjugates, which bind to primary amines, before and after processing with the MAGNIFY protocol. To compare to previous protocols, FFPE kidney samples were prepared following the modified expansion pathology (ExPath) protocol.[1,3] With this protocol, the samples were gelled in a monomer solution containing 15% (w/v) SA, 5% (w/v) AA, 0.1% (w/v) Bis, and 11.7% (w/v) NaCl along with 0.01% (w/v) 4HT, 0.2% (v/v) TEMED, and 0.2% (w/v) APS after overnight incubation with 0.05 mg/mL AcX. After homogenization with ProK digestion buffer (50 mM Tris (pH 8), 25 mM EDTA, 0.5% w/v TritonX, 0.8M NaCl, 2 units/mL ProK) for 3 hours at 60ºC, only 3.08 ± 0.46% s.e.m. (n = 9 technical replicates) of pre-expansion NHS fluorescence was observed (**Fig. 1e**). Meanwhile samples anchored with 0.05% (v/v) methacrolein using the MAGNIFY gel chemistry measured 14.54 ± 1.07% s.e.m. (n = 12 technical replicates) of pre-expansion NHS fluorescence (**Fig. 1e**) when homogenized under the same conditions. Similarly, for PFA fixed mouse brain tissue anchored with 0.1% (v/v) methacrolein or 0.05 mg/mL AcX using the MAGNIFY gel chemistry measured 6.19 ± 0.34% s.e.m. (n = 20 technical replicates) and 7.7% ± 0.41% s.e.m. (n = 20 technical replicates), respectively, retained fluorescence after 2 hours homogenization with ProK (**Fig. 1e**; **Supplementary Table 2**). Additionally, lipid retention between the two conditions was comparable for PFA fixed mouse brain tissue (**Supplementary Fig. 2f**; **Supplementary Table 3**), where lipid retention measured by retained fluorescence of DiD was 18.13 ± 0.44% s.e.m. (n = 20 technical replicates) and 17.06 ± 0.8% s.e.m. (n = 20 technical replicates) for 0.1% (v/v) methacrolein anchoring and AcX anchoring, respectively, after 2 hours homogenization with ProK. Lipid retention measurements are a comparison of the same lipophilic dye before and after expansion, and it is a measure of how much of the initial stain is still present after expansion. Therefore, if a particular structure is not stained by the lipophilic dye before expansion (such as the cell membrane) due to fixation, permeabilization, or other reasons, it cannot be revealed by MAGNIFY (**Supplementary Fig. 2e**).

To better preserve protein epitopes during expansion, we sought to replace the strong protease digestion of ProK. Protocols such as magnified analysis of the proteome (MAP)[4] utilize a non-enzymatic surfactant based tissue denaturing solution, but we found this to be incompatible with both AcX and methacrolein based anchoring (**Supplementary Fig. 2b**). Pulling from the ExPath[1] protocol and clearing protocols such as CUBIC,[5] we found that the addition of EDTA and urea, respectively, to a non-ionic surfactant based solution could adequately homogenize tissues and preserve biomolecules (**Supplementary Figure 2c**) while providing isotropic expansion. Indeed, both EDTA and urea were necessary to adequately homogenize samples anchored with methacrolein (**Supplementary Figure 2d**). Both AcX and methacrolein anchored FFPE kidney samples retained fluorescence after homogenizing using a non-ionic surfactant-based buffer (10% (w/v) SDS, 8M urea, 25 mM EDTA, 2x PBS pH 7.5 at RT) for 60 hours at 80 ºC (**Fig. 1e**; **Supplementary Fig. 2f**). AcX anchored samples retained 2.94 ± 0.57% s.e.m. (n = 14 technical replicates) NHS fluorescence which was comparable to the

ProK homogenized samples. Meanwhile, samples anchored with 0.05% (v/v) methacrolein retained 13.63 ± 1.86% s.e.m. (n = 8 technical replicates) under the same conditions. Similarly, PFA fixed mouse brain tissue retained48.44 ± 1.49% s.e.m. (n = 20 technical replicates) and 12.82 ± 0.77% s.e.m. (n = 20 technical replicates) NHS fluorescence for 0.1% methacrolein and AcX anchored samples, respectively, when homogenized for 4 and 8 hours, respectively, at 80ºC (**Fig. 1e; Supplementary Table 2**). Under the same conditions, MAGNIFY showed improved lipid retention, where 0.1% (v/v) methacrolein anchored samples retained 98.10 ± 2.36% s.e.m. (n = 30 technical replicates) DiD fluorescence and AcX anchored samples retained 74.50 ± 2.47% s.e.m. (n = 20 technical replicates) after homogenizing for 4 and 8 hours, respectively in hot surfactant (**Fig. 3a**; **Supplementary Fig. 2f; Supplementary Table 3**).

To improve biomolecule retention, we found that increasing the methacrolein to 0.25% (v/v) for FFPE kidney samples preserved 58.54% ±7.08% s.e.m. (n = 14 technical replicates) of the NHS signal after homogenization for 60 hours at 80 ºC using a surfactant-based buffer (**Supplementary Fig. 2f**). Similar to methacrolein concentration, homogenization time was tissue-type dependent (**Supplementary Table 1**), where mechanically tougher and more heavily fixed tissues such as FFPE kidney took longer to homogenize compared to softer tissue such as PFA fixed mouse brain. We also found the expansion factor was dependent on methacrolein concentration, where both over and under anchoring led to smaller expansion factors (**Supplementary Fig. 4**) and the optimal methacrolein concentration was tissue-type dependent (**Supplementary Table 1**).

Supplementary Note 2: Exploration of Gel Chemistry

To optimize gel chemistry, we first attempted to replicate the X10 protocol,[6] where N,N-dimethylacrylamide acid (DMAA) was used to obtain larger expansion factors. We first made a gelling solution of 26.7% (w/v) DMAA and 6.4% (w/v) SA was prepared in water. After dissolving, a 3.6% (w/v) stock solution of KPS was added to a final concentration 0.36% (w/v) KPS. After vortexing, TEMED (100% w/w) was added to a final concentration of 0.4% (v/v). To replicate ExPath gelling conditions, we chose to omit the nitrogen purging step. When applied to FFPE kidney pieces treated with AcX prior to gelling, incomplete expansion and non-isotropic expansion was observed in samples homogenized in a non-ionic surfactant-based buffer (10% (w/v) SDS, 8M urea, 25 mM EDTA, 2x PBS pH 7.5 at RT) for 60 hours at 80 ºC (**Supplementary Fig. 2c**). Additionally, these tissue gels were not mechanically sturdy and only had an expansion factor of ~6X when expanded in water (**Supplementary Table 6**; **Supplementary Fig. 4**).

To provide better expansion in a sturdy gel, we chose to supplement the ExPath gelling solution with a small amount of DMAA. To find the optimal gel composition, we varied DMAA, (SA, N,N′-Methylenebisacrylamide (Bis), and acrylamide (AA) concentrations and prepared blank gels and compared their expansion in water. From the rapid ExPath protocol, we knew increasing SA and AA concentrations provided a sturdier gel to compensate for the lower Bis concentration, which increased the expansion factor.[3] We ultimately found a hydrogel formula composed of 4% (w/v) DMAA, 34% (w/v) SA, 10% (w/v) AA, and 0.01% (w/v) Bis (**Supplementary Table 7**) provided the optimal expansion factor and sturdiness to expand an array of tissue types. We also varied the initiator, ammonium persulfate (APS), and inhibitor, 4-hydroxy-TEMPO (4HT), concentrations and polymerized the blank gels at different temperatures and found that a slightly higher APS concentration and slightly lower 4HT concentration compared to the ExPath protocol in gels polymerized at 37 ºC provided the best expansion factor (**Supplementary Table 8**). It should also be noted the blank gel expansion factors are slightly

smaller than that of the tissue-gel hydrogel due to different components contributed to the hydrogel by the tissue.

To test the mechanical sturdiness of the gel, we compared the MAGNIFY gel chemistry to other gels using the method developed for the Ten-fold Robust Expansion Microscopy (TREx) protocol (**Supplementary Fig. 4**, **Supplementary Table 9**).[7] The TREx gel is comprised of 11% (w/v) SA, 14.5% (w/v) AA, 0.005-0.015% (w/v) Bis, and 1x PBS along with 0.15% (v/v) TEMED, and 0.15% (w/v) APS. Briefly, blank gels were made in a 2 mL Eppendorf tube and then cut into half cylinders. The gels were then expanded in water and the deformation index was calculated by taking the ratio of radius of deformation to the radius of the gel. For the TREx protocol, higher crosslinker concentrations (150 ppm Bis) compared to the reported values (50 and 90 ppm Bis) were needed to produce a deformation index less than 0.5. Although both the high crosslinker TREx gel and MAGNIFY had similar expansion factors (5.9× in water), MAGNIFY had a much lower deformation index of 0.03 compared to the 0.28 deformation index of the high crosslinker TREx gel.

Interestingly, the crosslinking provided by the DMAA also had an anchoring effect; FFPE kidney samples anchored with 0% (v/v) methacrolein (**Supplementary Fig. 2c**) retained 2.43 ± 0.83% s.e.m. (n = 8 technical replicates) and 4.40 ± 0.64% s.e.m. when homogenized for 60 hours at 80 ºC using a surfactant-based buffer and with ProK homogenization using this gel chemistry.

Supplementary Note 3: DNA FISH experiment on tissues homogenized in MAGNIFY gel

We applied DNA FISH probes against telomere sequences[8] and centromere protein B box[9] to gel-embedded bladder cancer samples homogenized with strong ProK digestion, as ProK will homogenize proteins within tissue without damaging nucleic acids. We observed that these two probes diffused into MAGNIFY processed bladder cancer specimens and hybridized with chromosomal DNA within 2 hours at 37 °C and post-expansion lectin staining could still be performed (**Fig. 3m, Supplementary Video 5**). Using strong ProK digestion, we noticed that FFPE bladder cancer tissue section can be processed and stained with DNA FISH probes and lectin stain within 8 hours, which could be useful in time-sensitive applications such as those used for histopathological diagnoses.

Supplementary Note 4: Use of SOFI with MAGNIFY

We chose to pair MAGNIFY with SOFI as SOFI can work on standard fluorophores[10] images can be generated with under 100 time points[10], and it has been shown to work on 3D datasets[10,11]. SOFI was performed on expanded samples with custom MATLAB code, where images were corrected for drift, intensity and deconvolved (Lucy-Richardson method). The SOFI processing used in MAGNIFY-SOFI is based on CryoSOFI code[12], but only second-order cross-correlation was performed and the code was modified to function in 3D (Supplemental Fig. 6). We noted that the axial resolution improvement is limited as Z-planes were not captured simultaneously[11].

Supplementary Note 5: Parameter free resolution estimation of MAGNIFY-SOFI

To determine the effective resolution achieved by MAGNIFY and MAGNIFY-SOFI, we applied a parameter-free algorithm based on decorrelation analysis to images of human lung organoid (Apical out airway organoids[13], both normal and with CCDC39 gene mutations), expanded 10.5×. Organoids were stained with NHS-Cy3 and imaged using a CFI Plan Apochromat VC 60×C water immersion (1.2 NA), with a measured resolution of 36.52 ± 0.95

nm (**Supplementary Fig. 5d**). Lucy-Richardson deconvolution was then performed on the dataset, improving the measured resolution to 23.54 ± 1.31 nm. Finally, a resolution of 15.90 ± 1.39 nm was demonstrated on deconvolved MAGNIFY-SOFI images. Values are based on 37 measurements in each condition across 4 technical replicates. Depending on the wavelength of fluorophore, assuming the expansion factor is consistent with this organoid example, the effective resolution of MAGNIFY-SOFI is estimated to be 13~18 nm.

Supplementary Note 6: High-quality imaging of tubulin ultrastructure in cell culture.

U2OS cells (Gifted from the Lee lab at Carnegie Mellon University, originally purchased from ATCC) were grown in DMEM supplemented with 10% fetal bovine serum, 4.5 g/L D-glucose, 110 mg/L sodium pyruvate, 6 mM L-glutamine, 0.1 mM non-essential amino acids, 50 units/mL of penicillin and 50 µg/mL of streptomycin. Cells were not authenticated or tested for mycoplasma contamination. All cells were maintained at 37°C in a humid 5% CO_2 atmosphere. Cells were seeded unto #1.5 cover glass treated with poly-l-lysine in a 6 well plate and grown for 24-48 hours before fixation.

There is no standardized fixation protocol for super-resolution microtubule imaging, and thus this protocol requires optimization for new applications. The optimized protocol for high-quality tubulin images with MAGNIFY is based off existing protocols[14–17] and is as follows: 1 hour before fixation, media was replaced with DMEM containing 0% FBS and the cells were returned to the 37°C incubator. After the 1 hour, we brought the well plate out to a RT bench top, aspirated the media, and quickly extracted the coverslips (< 30 s) with freshly made 37°C PEMT buffer (80 mM PIPES, 5 mM EGTA, 2 mM $MgCl_2$ at pH 6.8 with 0.2% TX). Quickly after we aspirated the PEMT buffer, we applied 2 ml of cold -20°C 100% methanol to each well, and then fixed the plate at -20°C for 5 minutes. We then replaced the methanol with RT 1x PBS and performed consecutive washes with PBS for 30 s, 1 min, 5 min, 10 min, and 15 min, using the 2-handed pipette technique to reduce mechanical stress and further dehydration.

MAGNIFY gel monomer solution was prepared as described in the methods section. Prior to gelation, methanol fixed U2OS cell samples were placed into custom gelling chamber consisting of four spacers cut from #1.5 cover glass adhered to the uncoated back of a microscope slide on either side of a piece of cover glass with adherent cells. Excess PBS around the specimen was absorbed with a Kimwipe and the specimen was allowed to air dry partially. Immediately prior to gelation, the chemicals APS, TEMED, and methacrolein were added to a final concentration of 0.25% (w/v) APS, 0.2% TEMED (w/v), and 0.1% (v/v) methacrolein. The solution was vortexed and applied to the U2OS cells. A glass slide was then placed on top of the double-stacked spacers to form a gelling chamber. The samples were incubated overnight in a humidified container at 37 ºC to complete gelation.

After gelation, the glass slide cover was removed from the gelling chamber, blank gel surrounding the tissue was trimmed from the samples, and the U2OS cell containing hydrogel was cut into smaller pieces. Samples were then incubated in homogenization buffer (10% w/v SDS, 8M Urea, 25 mM EDTA, 2× PBS, pH 7.5 at RT) for 36h at 80 ºC with shaking. Homogenized samples were then washed 3 times with 1× PBS at RT, followed by at least 3 washes in 1% decaethylene glycol monododecyl ether ($C_{12}E_{10}$)/1xPBS or 1%PBST at RT or 60 ºC to remove remaining SDS.

After homogenization and washing, U2OS cells were stained with approximately 1 µg/mL of a combination of rabbit anti-αTubulin (Abcam ab18251, Proteintech 80762-1-RR, 11224-1-AP), rabbit anti-βTubulin (Proteintech 10094-1-AP), mouse anti-αTubulin (Invitrogen

62204, Proteintech 66031-1-Ig), and mouse anti-βTubulin (Proteintech 66240-1-Ig, Sigma T8328) in staining buffer (5× SSC (750 mM NaCl, 75 mM sodium citrate, pH 7.0)/0.1% Tween 20) for 48h RT. Samples were then washed 3 times with washing buffer (1×PBS/0.1% ($C_{12}E_{10}$)/1xPBS) at RT for at least 10 minutes. To prevent disassociation during full expansion in water, samples were stained with both fluorescently conjugated secondaries and biotin-streptavidin labeling. First, samples were incubated in staining buffer with AF488 AffiniPure Fab Fragment Donkey Anti-Rabbit (Jackson ImmunoResearch 711-547-003) , AF488 AffiniPure Fab Fragment Donkey Anti-Mouse (715-547-003), Biotin-SP AffiniPure Fab Fragment Goat Anti-Rabbit (111-067-003), and Biotin-SP AffiniPure Fab Fragment Goat Anti-Mouse (115-067-003) diluted to approximately 1 μg/mL in staining buffer for at least 1 hour at RT. Samples were then washed at least 3 times with washing buffer for at least 10 minutes. Samples were then incubated with AF488 conjugated streptavidin (Invitrogen S11223) diluted to approximately 1 μg/mL in staining buffer for at least 20 minutes at RT. Samples were then washed at least 3 times with washing buffer for at least 10 minutes. After staining, samples were washed in water for at least 10 minutes. This was repeated until the sample was fully expanded, at least three exchanges of water.

Supplementary Figures

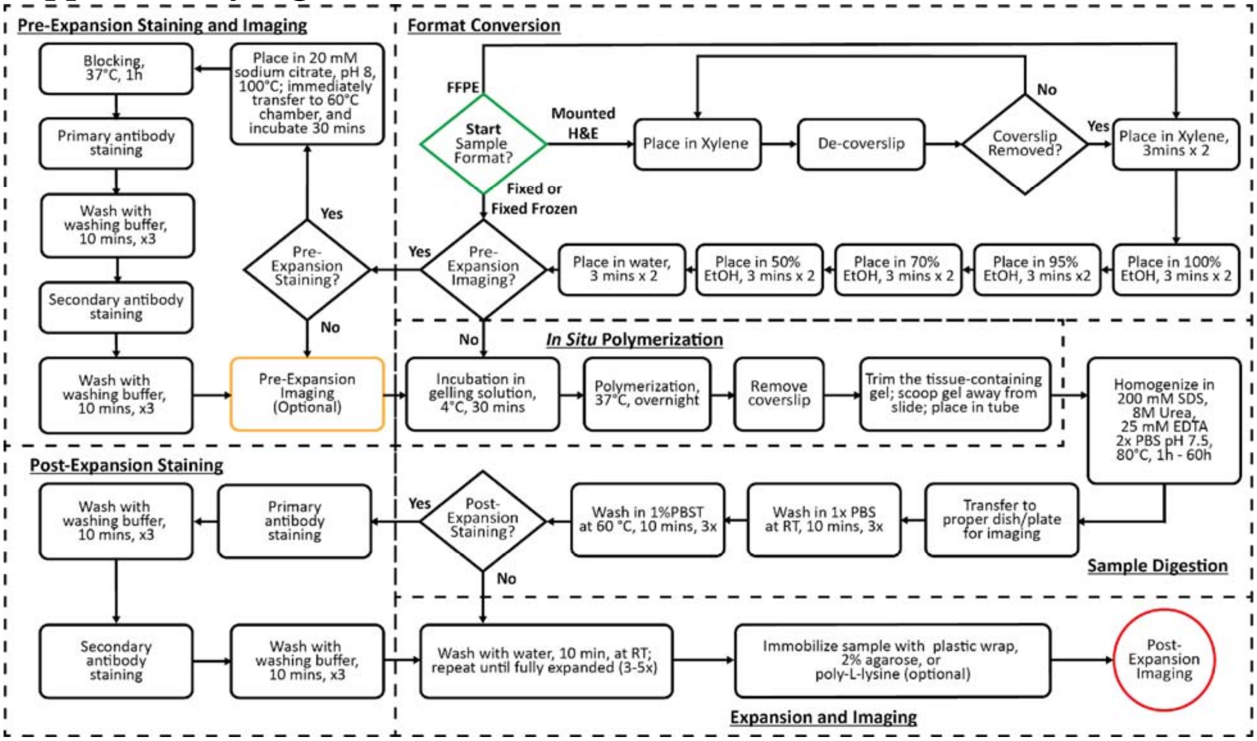

Supplementary Figure 1: ***The full workflow for MAGNIFY.***

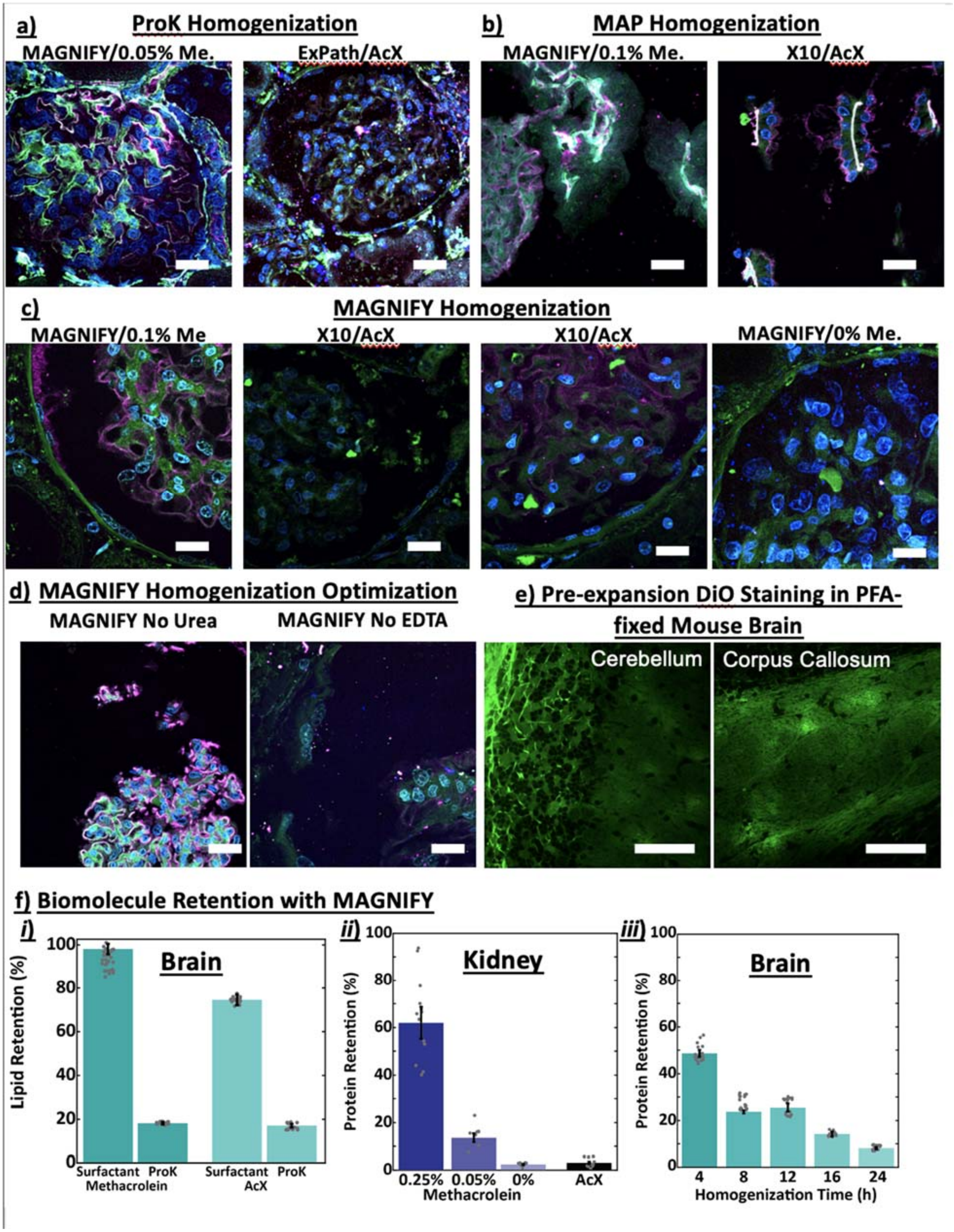
a) ProK Homogenization
MAGNIFY/0.05% Me.
ExPath/AcX
b) MAP Homogenization
MAGNIFY/0.1% Me.
X10/AcX
c) MAGNIFY Homogenization
MAGNIFY/0.1% Me
X10/AcX
X10/AcX
MAGNIFY/0% Me.
d) MAGNIFY Homogenization Optimization
MAGNIFY No Urea
MAGNIFY No EDTA
e) Pre-expansion DiO Staining in PFA-fixed Mouse Brain
Cerebellum
Corpus Callosum
f) Biomolecule Retention with MAGNIFY
i)
Brain
Lipid Retention (%)
0
20
40
60
80
100
Surfactant
ProK
Methacrolein
Surfactant
ProK
AcX
ii)
Kidney
Protein Retention (%)
0
20
40
60
80
100
0.25%
0.05%
0%
AcX
Methacrolein
iii)
Brain
Protein Retention (%)
0
20
40
60
80
100
4
8
12
16
24
Homogenization Time (h)

Supplementary Figure 2: ***Comparison of anchoring, homogenization, and gelling strategies.*** **(a)** Deparaffinized and stained FFPE kidney samples were gelled according to the MAGNIFY or ExPath protocols and were incubated in the ExPath homogenization buffer (50 mM Tris pH 8, 25 mM EDTA, 0.5% w/v TritonX, 0.8M NaCl) with Proteinase K diluted by 1:200 (final concentration 4 units/mL) for 3 hours at 60 °C. Final expansion factors: 4.2 (MAGNIFY) and 3.3 (ExPath). **(b)** Kidney samples prepared with the MAGNIFY protocol anchored with 0.1% (v/v) methacrolein, X10 protocol anchored with AcX, and the MAGNIFY protocol showed severe cracking due to incomplete homogenization when expanded in water after homogenizing for 60h at 80°C in the MAP homogenization buffer (200 mM SDS, 200 mM NaCl, and 50 mM Tris in water pH 9 at RT). **(c)** Samples prepared with the MAGINFY protocol anchored with 0.1% (v/v) methacrolein, ExPath protocol anchored with AcX, and the x10 protocol anchored with AcX could be expanded in water after homogenization using a non-ionic surfactant buffer (10% w/v SDS, 8M Urea, 25 mM EDTA, 2x PBS, pH 7.5 at RT) for 60h at 80°C. However, the x10 prepared samples had less even homogenization and smaller expansion compared to the MAGNIFY sample. Some anchoring is present in samples without an anchoring agent, most likely due to the crosslinking capability of DMAA. Optimal anchoring was observed in 0.25% (v/v) methacrolein anchored kidney samples. Expansion factors: 7.9 (MAGNIFY/0.1%Me.) and 6.0 (X10). **(d)** When urea or EDTA were removed from the non-ionic surfactant buffer, insufficient homogenization led to incomplete expansion and severe cracking in FFPE kidney samples homogenized for 60h at 80°C. Gels were expanded in water and representative maximum intensity projection images were obtained at 10x magnification. Scale bar 100 μm (absolute scale) Blue DAPI, green NHS, magenta WGA. **(e)** Pre-expansion DiO staining in the cerebellum (left) and corpus callosum (right) in PFA-fixed mouse brain tissue showing strong myelin staining but no strong staining of the neuronal plasma membrane. Scale bar: 50 μm. **(f)** Protein and Lipid retention for mouse brain and human kidney. ***(i)*** Lipid retention measured as DiD fluorescence retention for mouse brain homogenized in surfactant versus ProK for tissues anchored with 0.1% methacrolein or AcX. N = 20 technical replicates. Error bars in terms of s.e.m. ***(ii)*** Protein retention of human kidney measured as NHS fluorescence retention processed with MAGNIFY versus ExPath and anchored with different conditions and homogenized in hot surfactant for 60 hours. N = 13, 8, 8, and 9 technical replicates**.** ***(iii)*** Protein retention of mouse brain measured as NHS fluorescence retention processed with MAGNIFY and anchored with 0.1% methacrolein and homogenized in hot surfactant over time. N = 20 technical replicates.

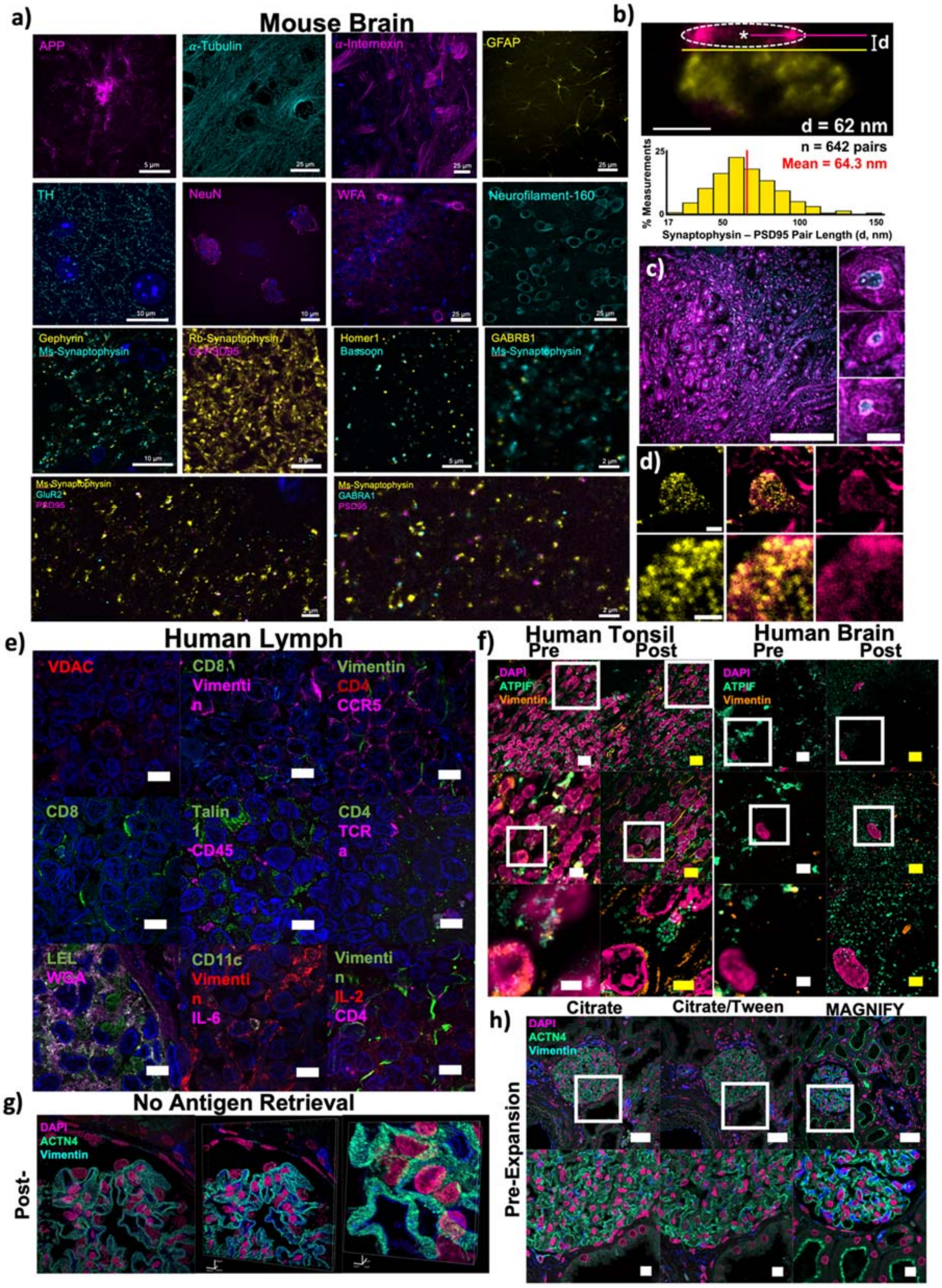

Supplementary Figure 3: ***Examples of Post Expansion Staining with MAGNIFY*** **(a)** Paraformaldehyde-fixed 30µm mouse brain sections were gelled according to the MAGNIFY protocol using 0.1%-1% (v/v) methacrolein and were homogenized using a non-ionic surfactant buffer (10% w/v

SDS, 8M Urea, 5-25 mM EDTA, 1-2x PBS, pH 7.5 at RT) for 4-8h at 80°C. After expansion, samples were stained with indicated primary antibodies in 1x PBS overnight. After washing, samples were stained with DAPI and relevant secondary antibodies. After staining, samples were washed in 1x PBS and imaged at 10x (APP, GFAP, WFA, Neurofilament-160) or 40x (α-Tubulin, α-Internexin, TH, NeuN, all synaptic markers) magnification. Blue, DAPI. **(b)** Quantification of synaptophysin-PSD95 pair distances in mouse striatum expanded fully with MAGNIFY. Top: Example measurement. The distance, *d*, was taken from the edge of synaptophysin (yellow) to the center of the postsynaptic density (PSD95, magenta). Bottom: Summary of all measurements taken. Y-axis represents the number of measurements as a percentage of all measurements taken. n = 642 measurements across two technical replicates. Mean pair distance = 64.3 nm. Expansion factor: 11× in ddH_2O. **(c)** Visualization of myelinated axons in the mouse brain with MAGNIFY. Cyan: NHS-Cy3, Magenta: DiD. Scale bars: Overview: 10 µm, Zoom-ins: 1 µm. **(d)** Lipophilic dye specificity for synaptic vesicles in the mouse brain. Left: synaptophysin, yellow. Right: DiD, magenta. Middle, merge. Bottom Row: Zoom-in of top row. Areas of high vesicle density (as shown by synaptophysin labeling) can be seen to be labeled with DiD as well. Scale bar top: 200nm; bottom: 100 nm. **(e)** Deparaffinized human lymph node samples purchased from US Biomax were gelled according to the MAGNIFY protocol using 0.1% (v/v) methacrolein and were homogenized using a non-ionic surfactant buffer (10% w/v SDS, 8M Urea, 5-25 mM EDTA, 1-2x PBS, pH 7.5 at RT) for 26-32h at 80°C. After expansion, samples were stained with indicated primary antibodies in staining buffer overnight. After washing, samples were stained with DAPI and relevant secondary antibodies. After staining, samples were washed in 1xPBS and imaged at 40x magnification. Scale bar 50 um (absolute scale) Blue DAPI. **(f)** MAGNIFY improves post expansion staining. Human tonsil (left) and brain (right) FFPE samples were imaged at 40x (top left, scale bar 10 µm, middle left scale bar 5µm, bottom left scale bar 2 µm). The white box indicates the field of view of the higher magnification images. The samples were then processed with the MAGNIFY protocol, and the same fields of view were imaged post-expansion in water at 10× (top right, scale bar, 10 µm biological scale) and 40x (middle right scale bar 5µm, bottom right scale bar 2 µm; biological scale). Post expansion images were projected over 4-17 z slices. Expansion factors in water were: 8.04× (tonsil), 8.36× (brain). Staining of ATIPF and vimentin improve with post expansion staining. Magenta, DAPI; Green, ATPIF; Orange, Vimentin. **(g)** MAGNIFY does not require antigen retrieval. MAGNIFY protocol achieves good post expansion staining without the need for antigen retrieval pre-expansion. Scale bar 25 µm (absolute scale). Foot processes in kidney podocytes can still be resolved. Magenta, DAPI; Green, ACTN4, Blue, Vimentin. **(h)** The MAGNIFY homogenization buffer can be used as a superior pre-expansion antigen retrieval buffer**.** After heat treatment for 30-45 minutes in 200 mM sodium citrate (pH 8), 200 mM sodium citrate, 0.05% tween (pH 6.5), or 10% w/v SDS, 8M Urea, 25 mM EDTA, 2x PBS (pH 9.5), the MAGNIFY buffer demonstrates superior pre-expansion staining. Scale bar top: 50 µm; bottom: 10 µm.

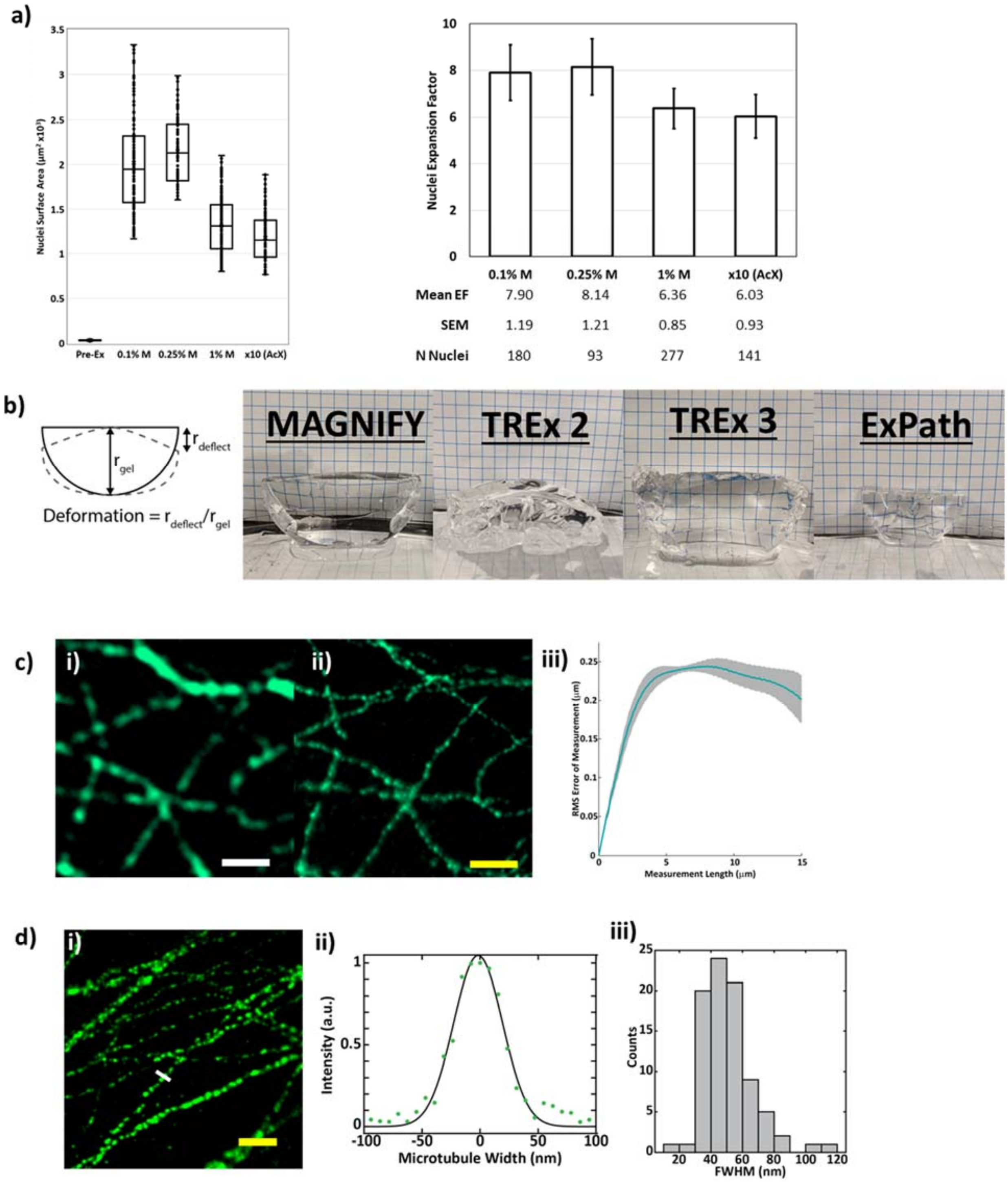

Supplementary Figure 4: ***Exploration and characterization of MAGNIFY gel chemistry.*** **(a)** Estimation of Expansion Factors for Gelling and Anchoring Strategies. Kidney samples prepared with the x10 protocol or MAGNIFY with different methacrolein concentrations. All samples were homogenized for 60 hours at 80 °C. To estimate expansion factors, average nuclear areas were measured in ImageJ (left). The box defines the interquartile range, divided by the median.

The whiskers delineate the minimum and maximum values for each data set. Each anchoring condition was then divided by the average nuclear size from the pre-expansion images and then averaged to calculate expansion factors for the different anchoring strategies (right) with mean expansion factors, sdandard error of the mean (SEM) values, and n nuclei measured listed in the table below. Error bars are given in terms of SEM. **(b)** Measurement of Deformation of different gel chemistries. Blank gels were prepared in 2 mL Eppendorf tubes and gelled at 37 °C or 45 °C and fully expanded in water. Deformation was calculated in the same method described in the TREx paper[7], by taking the ratio of the radius of deformation to the radius of the gel. Squares are 5 mm. **(c)** Example images of ***(i)*** pre-expansion images of HEK cells stained with αTubulin (scale bar 1µm) imaged using a Nikon CFI Plan Apo VC 60× C WI (1.2 NA) objective and processed with SOFI compared to the same field of view ***(ii)*** post-expansion with MAGNIFY imaged with a Nikon APO LWD 20× (NA 0.95) WI Lambda S objective (scale bar 1 µm biological scale, 9.22 µm physical scale, expansion factor, 9.22×). ***(iii)*** Root mean square (RMS) length measurement error as a function of measurement length for pre-expansion versus post expansion images. Solid line, mean of channel; shaded area, standard error of mean; n = 3 technical replicates.

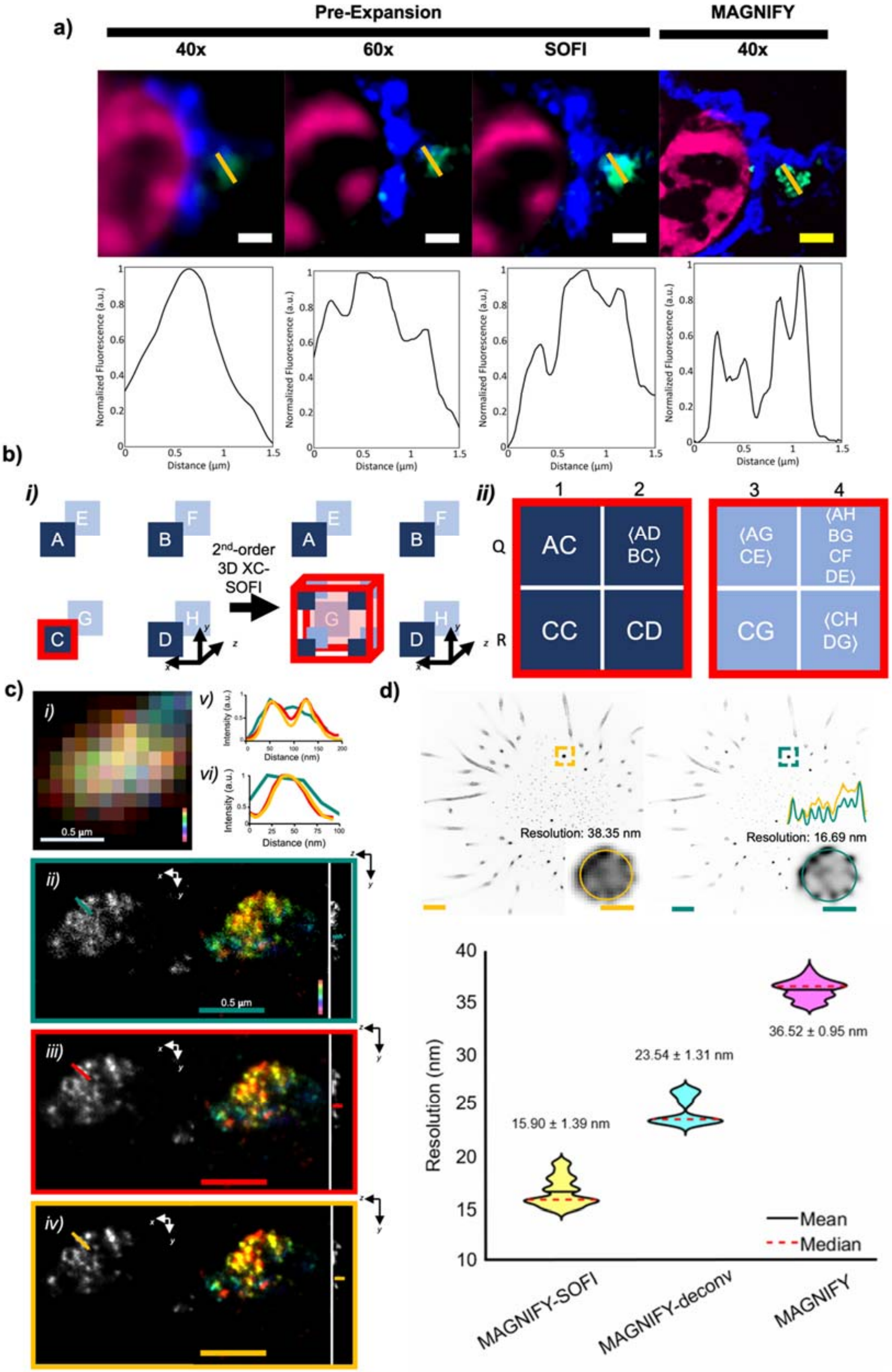
a)
Pre-Expansion
MAGNIFY
40x
60x
SOFI
40x
Normalized Fluorescence (a.u.)
Distance (µm)
b)
i)
A
B
C
D
E
F
G
H
2nd-order 3D XC-SOFI
ii)
1
2
3
4
Q
R
AC
⟨AD BC⟩
CC
CD
⟨AG CE⟩
⟨AH BG CF DE⟩
CG
⟨CH DG⟩
c)
i)
0.5 µm
v)
vi)
Intensity (a.u.)
Distance (nm)
ii)
iii)
iv)
d)
Resolution: 38.35 nm
Resolution: 16.69 nm
Resolution (nm)
15.90 ± 1.39 nm
23.54 ± 1.31 nm
36.52 ± 0.95 nm
Mean
Median
MAGNIFY-SOFI
MAGNIFY-deconv
MAGNIFY

Supplementary Figure 5: ***Overview of SOFI*** **(a)** Second-order SOFI improves resolution pre-expansion. Top: Placenta tissue imaged pre-expansion at both 40× and 60× and post-expansion at 40×. Bottom: line profile of ATPIF channel indicated by orange line. 60× images were generated by performing a maximum intensity projection over 50 time points. This same set of images at 60× was processed using second-order SOFI with deconvolution. Resolution is seen to improve. Post-expansion images of the MAGNIFY processed sample show greater resolution enhancement. Scale bar: 1 μm (post-expansion 8.75 μm). Magenta: DAPI, Green: ATPIF, Blue: Pan-Keratin. **(b)** Generation of 3D XC-SOFI images **(*i*)** Each pixel in the original image becomes eight in the 2nd – order 3D XC-SOFI image by calculating its correlation with neighboring pixels. **(*ii*)** The generation of each new pixel in the 2nd – order 3D XC-SOFI image is given. For example, New Pixel R1 is given by calculating the autocorrelation of Pixel C in the original image, while New Pixel R2 is given by calculating the correlation between Pixels C and D. The values of New Pixels Q2, Q3, Q4, and R4 were calculated by averaging the correlations of multiple sets of Original Pixels. **(c)** Three-dimensional resolution improvement with MAGNIFY-SOFI. **(*i*)** A color-coded Z-projection of a pre-synapse stained for synaptophysin in a mouse brain before MAGNIFY. **(*ii*)** A separate synaptophysin stained pre-synapse in a mouse brain after MAGNIFY. Middle: Color-coded Z-projection of the synapse. Individual presynaptic vesicles are beginning to be resolved. Right: YZ view of the pre-synapse. Green line demarcates where line profile in **(*vi*)** is taken. Left: Single XY plane of the synapse. Green line demarcates where line profile in **(*v*)** is taken. **(*iii*)** As in **(*ii*)** but after 2nd-order 3D XC-SOFI. **(*iv*)** As in **(*ii*)** but after 3rd-order 3D XC-SOFI. **(v).** XY intensity line profiles showing increased resolving power of MAGNIFY-SOFI. **(*vi*)** YZ intensity line profiles showing increased resolving power of MAGNIFY-SOFI**. (d)** Parameter-free image resolution estimation of MAGNIFY (top left panel) and MAGNIFY-SOFI (top right panel) images based on decorrelation analysis. Example images were acquired from a fully expanded human stem cell-derived lung organoid. Line profiles around an example cilium are shown for both MAGNIFY (yellow) and MAGNIFY-SOFI (green) in the right panel, demonstrating the resolution improvement. Bottom: violin plot of calculated resolutions of MANGIFY-SOFI (yellow), MAGNIFY with deconvolution (MAGNIFY-deconv; cyan), and MAGNIFY (magenta). N = 37 measurements from 4 technical replicates. Scale bar: 300 nm, inset: 100 nm. All in biological scales. Expansion factor: 10.5 ×.

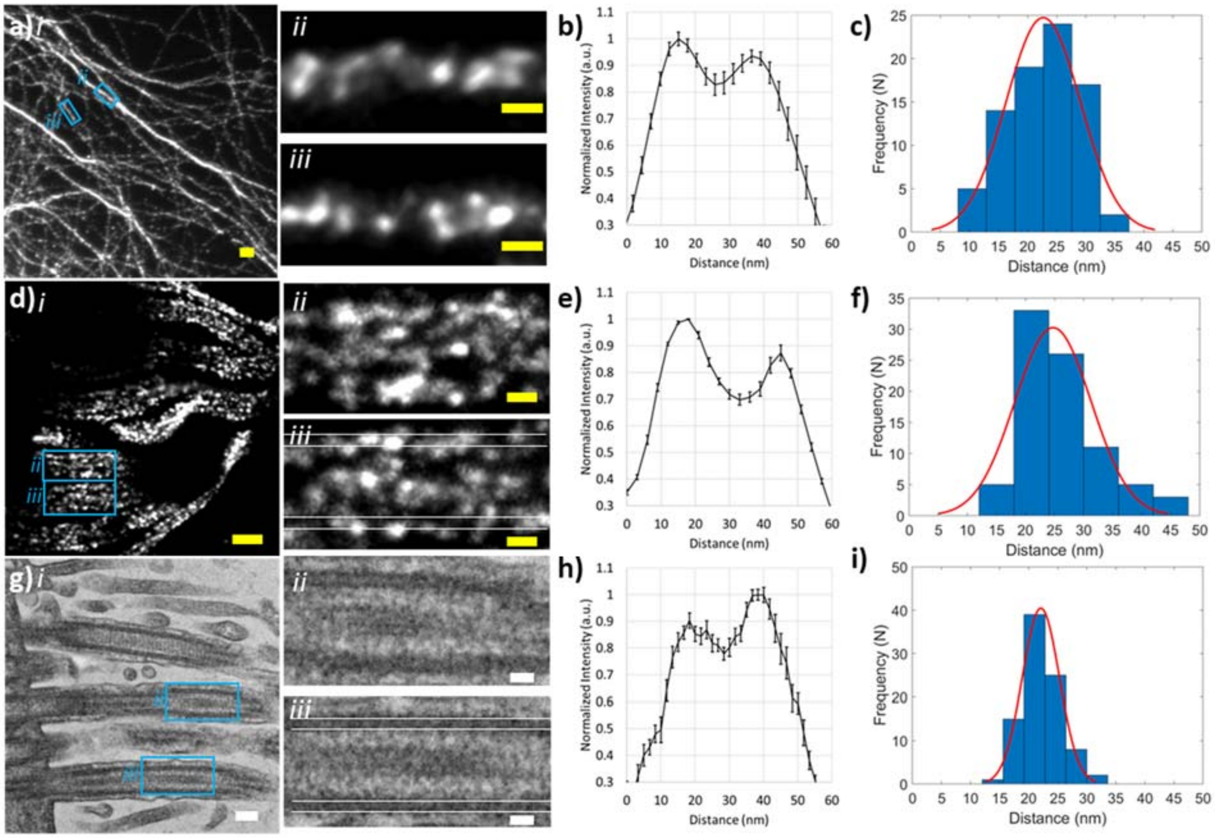

Supplementary Figure 6: ***Example images of tubulin structure in cell culture and cilia in human lung organoids.*** **(a)** Fluorescence images of alpha and beta tubulin stained MAGNIFY-processed fully expanded U2OS cells at 40× magnification. *ii* and *iii* show third order SOFI processed fluorescent images at 60× magnification of regions indicated in *i*. **(b)** Example average line profile averaged over 13 segments. Segments were averaged over 5 pixels. Error bars in terms of SEM. **(c)** Peak-to-peak distances of line profiles of 81 segments of expanded U2OS cells. The average over all distances was 22.68 nm ± 0.71 nm (mean ± s.e.m.). **(d)** Second order SOFI processed fluorescent images at 60× magnification of cilia in fully expanded MAGNIFY-processed human lung organoids stained with alpha and beta tubulin. *ii* and *iii* show zoomed in regions indicated in *i*. **(e)** Example average line profile averaged over 7 segments. Segments were averaged over 5 pixels. Error bars in terms of SEM. **(f)** Peak-to-peak distances of line profiles of 83 segments of expanded cilia. The average over all distances was 24.72 nm ± 0.72 nm (mean ± s.e.m.). **(g)** EM images of cilia in human lung organoids. *ii* and *iii* show zoomed in regions indicated in *i*. **(h)** Example average line profile averaged over 13 segments. Segments were averaged over 5 pixels. Error bars in terms of SEM. **(i) P**eak-to-peak distances of line profiles of 90 segments of unexpanded cilia imaged with EM. The average over all distances was 22.09 nm ± 0.34 nm (mean ± s.e.m.). Scale bars (biological scale): a,d,g *i*: 250 nm, a,d,g *ii*-*iii*: 50nm.

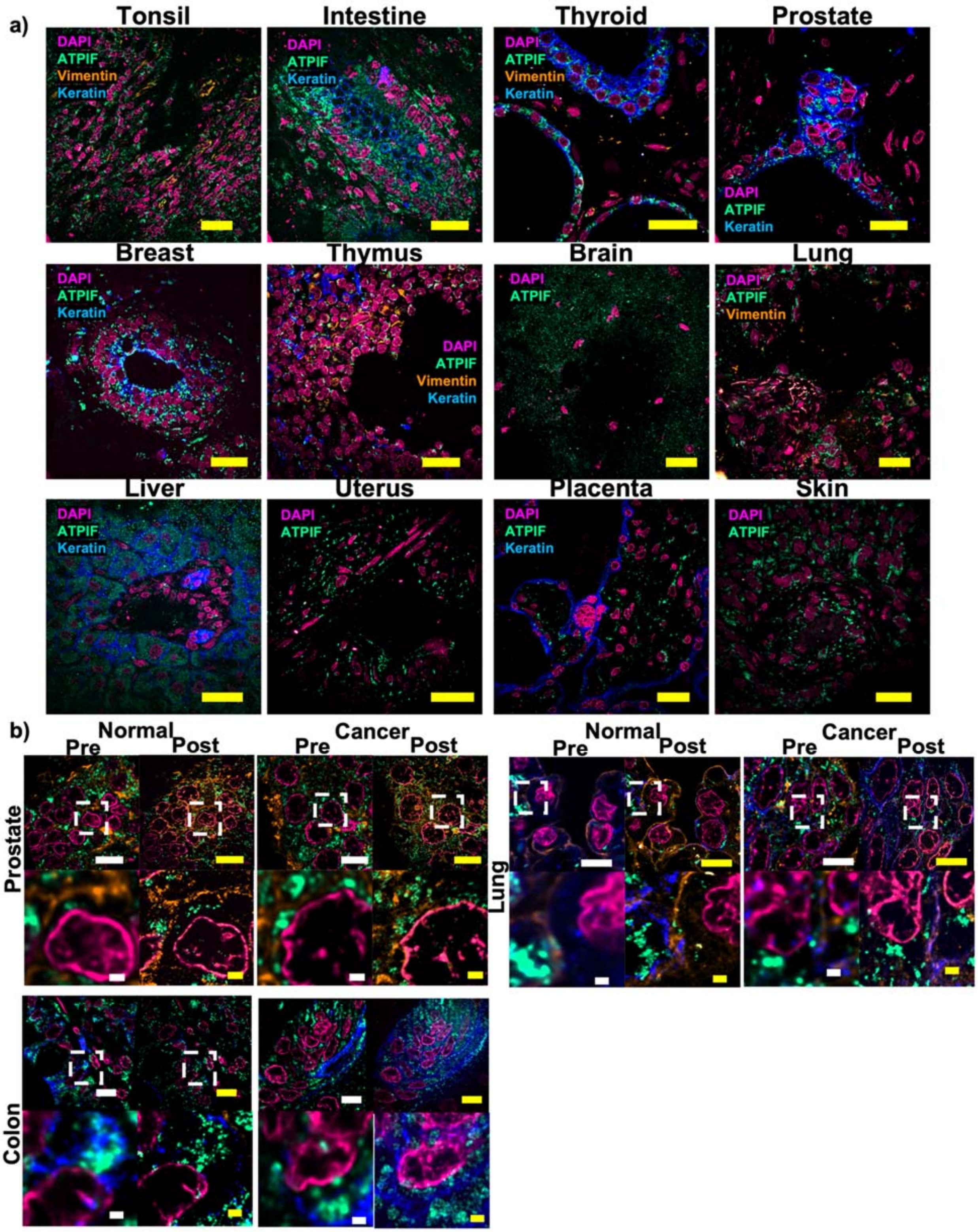
a)
Tonsil
DAPI
ATPIF
Vimentin
Keratin
Intestine
DAPI
ATPIF
Keratin
Thyroid
DAPI
ATPIF
Vimentin
Keratin
Prostate
DAPI
ATPIF
Keratin
Breast
DAPI
ATPIF
Keratin
Thymus
DAPI
ATPIF
Vimentin
Keratin
Brain
DAPI
ATPIF
Lung
DAPI
ATPIF
Vimentin
Liver
DAPI
ATPIF
Keratin
Uterus
DAPI
ATPIF
Placenta
DAPI
ATPIF
Keratin
Skin
DAPI
ATPIF
b)
Normal
Pre
Post
Cancer
Pre
Post
Prostate
Normal
Pre
Post
Cancer
Pre
Post
Lung
Colon

Supplementary Figure 7*: Example images of MAGNIFY expansion of FFPE tissue.* **(a)** Successful expansion of multiple human tissue types processed using the MAGNIFY framework. Images were obtained at 10× magnification. Scale bars are 25 µm (biological scale). Expansion factors are given in **Supplementary Table 1. (b)** Images of various tissue types for both normal (left images) and cancerous (right images) human tissues. Within each block of images for a given tissue disease type, there are four images shown. The left column shows two images, the top of which is a field of view (scale bar, 10 µm), and the bottom of which zooms into the area outlined in the top image by a red dash box (scale bar, 1 µm). The right column within the four images shows the same fields of view as are shown in the left column, but post-expansion (Scale bars are kept in the same biological scale: top images, 10 µm; bottom images, 1 µm; expansion factors: 10.4×, 10.8×, and 9.8× in water for prostate, lung, and colon, respectively); Magenta, DAPI; Green, ATPIF; Orange, WGA; Blue, PanK.

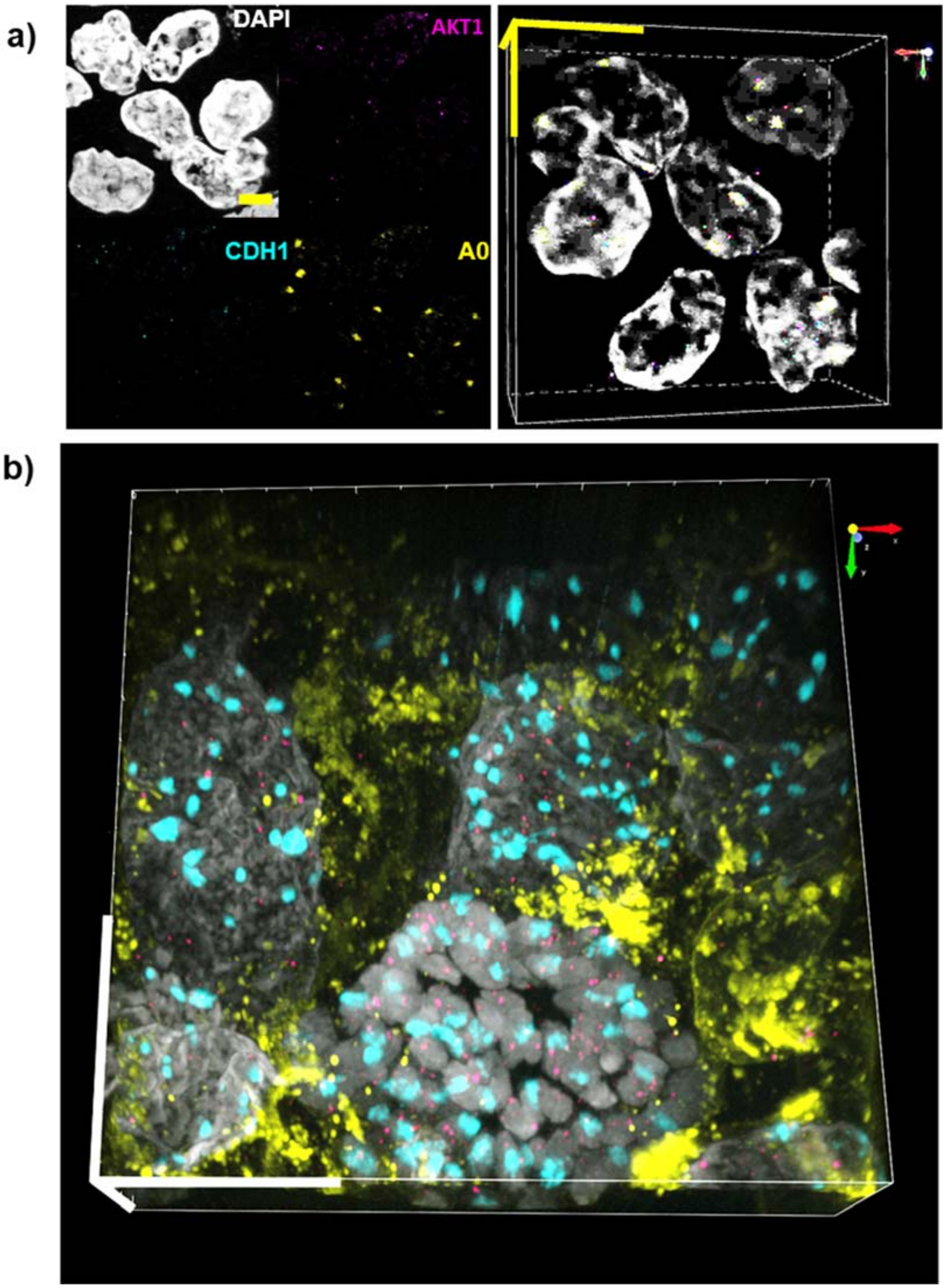

Supplementary Figure 8: ***DNA FISH with MAGNIFY using FFPE human tissue.*** After tissue section recovery, samples were gelled using the MAGNIFY gel and anchored with 0.25% methacrolein. After gelling, samples were treated with hot surfactant (1% w/v SDS, 8M Urea, 25 mM EDTA, 2× PBS, pH 7.5) for 48 hours at 80 °C, labeled with respective DNA FISH probes, and xpanded in 1xPBS before imaging. **(a)** Normal human lymph node tissue. Scale bars: Left: 2.5 µm, Right: x: 5 µm, y: 5 µm, z: 5.7 µm. Expansion factor: 3.5× in 1× PBS. White, DAPI; Magenta, AKT1; Blue, CDH1, Yellow, A0. (**b**) 3D reconstruction of confocal images of expanded human urinary bladder cancer tissue section, homogenized by proteinase K digestion.

Expansion factor 3.8× (in PBS buffer). Stain: Gray, DAPI; Cyan, DNA FISH probe against centromere binding protein B box motif CEPN-B; Magenta, DNA FISH probe against telomere motif TelC; Yellow, Wheat Germ Agglutinin. Scale bar: x: 20 µm; y: 20 µm; z: 20 µm.

Supplementary Videos

Supplementary Video 1. 3D rendering of a fully expanded MAGNIFY processed human FFPE kidney tissue stained with DAPI (magenta), ACTN4 (orange), and WGA (blue) taken at 40× magnification.

Supplementary Video 2. 3D rendering of a fully expanded MAGNIFY processed human FFPE colon tissue stained with DAPI (magenta), ATPIF (green), and Cytokeratin Pan Type I/II (blue) taken at 40× magnification.

Supplementary Video 3. 3D rendering of a fully expanded MAGNIFY processed human FFPE placenta tissue stained with DAPI (magenta), ATPIF (green), and Cytokeratin Pan Type I/II (blue) taken at 40× magnification.

Supplementary Video 4. 3D rendering of a fully expanded MAGNIFY processed human FFPE breast tissue stained with DAPI (magenta), ATPIF (green), and Cytokeratin Pan Type I/II (blue) taken at 40× magnification.

Supplementary Video 5. 3D rendering of an expanded human urinary bladder cancer tissue section stained with DAPI (white), DNA FISH probe against centromere binding protein B box motif CEPN-B (cyan), DNA FISH probe against telomere motif TelC, and WGA (yellow) taken at 40× magnification.

Supplementary Video 6. 3D rendering of an SST neuron in MAGNIFY processed mouse brain stained with DAPI (white), anti-GFP (blue), synaptophysin (magenta) and PSD95 (green) expanded in 1x PBS and taken at 40× magnification.

Supplementary Video 7. 3D rendering of SST dendrites in MAGNIFY processed mouse brain stained with DAPI (white), anti-GFP (blue), synaptophysin (magenta) and PSD95 (green) expanded in 1x PBS and taken at 40× magnification.

Supplementary Video 8. 3D rendering of SST dendrites in MAGNIFY processed mouse brain stained with DAPI (white), anti-GFP (blue), synaptophysin (magenta) and PSD95 (green) expanded in 1x PBS and taken at 40× magnification.

Supplementary Video 9. 3D rendering of a MAGNIFY-SOFI image stack of fully expanded ependymal cilia and basal bodies from the ependymal cell lining in the adult mouse brain stained with NHS-ATTO-488 taken at 40× magnification.

Supplementary Tables

Supplementary Table 1: ***Condition optimization for different tissue types***

Tissue Type	Fixation	Methacrolein	Hom. Time	Hom. Temp.	Expansion Factor
Human Kidney	FFPE	0.25%	60 h	80C	8.64± 0.24 (N = 4)*
Human Lymph Node	FFPE	0.25%	60 h	80C	8.46± 0.47 (N = 5)*
Human Tonsil	FFPE	0.25%	60 h	80C	8.62 ± 0.27 (N = 6)*
Human Colon	FFPE	0.25%	60 h	80C	9.67 ± 0.35 (N = 3)*
Human Thyroid	FFPE	0.25%	60 h	80C	10.75 ± 0.35 (N = 3)*
Human Prostate	FFPE	0.25%	60 h	80C	10.38 ± 0.57 (N = 3)*
Human Breast	FFPE	0.25%	60 h	80C	9.03 ± 0.22 (N = 3)*
Human Thymus	FFPE	0.25%	60 h	80C	10.00 ± 0.37 (N = 3)*
Human Brain	FFPE	0.25%	10 h	80C	8.36 ± 0.25 (N = 3)*
Human Lung	FFPE	0.25%	60 h	80C	10.77± 0.61 (N = 3)*
Human Liver	FFPE	0.25%	60 h	80C	10.13± 0.60 (N = 3)*
Human Uterus	FFPE	0.25%	60 h	80C	8.00 ± 0.28 (N = 3)*
Human Placenta	FFPE	0.25%	60 h	80C	8.75 ± 0.37 (N = 3)*
Human Skin	FFPE	0.25%	60 h	80C	9.39 ± 0.30 (N = 3)*
Mouse Brain	PFA	0.1%	8 h	80C	11.18 ± 1.87 (N = 3)*†
Lung Organoid	PFA	0.1%	8 h	80C	9.9 ± 2.1 (N=3)
HEK Cells	PFA/GA	0.1%	6 h	80C	9.22 ± 2.3 (N=6) *

***** *Error in terms of s.e.m. over N technical replicates.*

† Mouse brain expansion factor was calculated by measuring different nuclei sizes in mouse striatum before and after expansion.

Supplementary Table 2: ***Protein Retention for tissues under different anchoring and homogenization conditions.***

	Anchoring		Homogenization		Protein Retention		
**Tissue**	**Type**	**Conc.**	**Type**	**Time**	**%Retention**	**SEM**	**N**
**Kidney**	Methcarolein	0.25%	Surfactant	60h	61.99	6.66	13
	Methcarolein	0.05%	Surfactant	60h	13.63	1.86	8
	Methcarolein	0.00%	Surfactant	60h	2.42	0.28	8
	AcX	0.05 mg/mL	Surfactant	60h	2.94	0.57	14
	Methcarolein	0.05%	ProK	3h	14.54	1.07	12
	AcX	0.05 mg/mL	ProK	3h	3.08	0.46	9
**Brain**	Methcarolein	0.10%	Surfactant	4h	48.44	1.49	20
	Methcarolein	0.10%	Surfactant	8h	23.76	0.69	20
	Methcarolein	0.10%	Surfactant	12h	25.56	1.62	20
	Methcarolein	0.10%	Surfactant	16h	14.20	1.06	20
	Methcarolein	0.10%	Surfactant	24h	8.09	0.47	20
	AcX	0.05 mg/mL	Surfactant	8h	12.82	0.77	20
	Methcarolein	0.10%	ProK	2h	6.19	0.34	20
	AcX	0.05 mg/mL	ProK	2h	7.72	0.41	20
**Organoid**	Methacrolein	0.10%	Surfactant	8h	76.23	8.46	13

***** Error in terms of s.e.m. over N technical replicates.

Supplementary Table 3: ***Lipid retention in mouse brain under different anchoring and homogenization conditions.***

Anchoring		Homogenization		Lipid Retention		
**Type**	**Concentration**	**Type**	**Time**	**%Retention**	**SEM**	**N**
Methcarolein	0.10%	Surfactant	4h	98.10	2.36	30
Methcarolein	0.10%	Surfactant	8h	77.05	1.80	20
Methcarolein	0.10%	Surfactant	12h	73.23	2.99	20
Methcarolein	0.10%	Surfactant	16h	63.60	3.06	20
Methcarolein	0.10%	Surfactant	24h	43.07	3.06	20
AcX	0.05 mg/mL	Surfactant	8h	74.50	2.47	20
Methcarolein	0.10%	ProK	2h	18.13	0.54	20
AcX	0.05 mg/mL	ProK	2h	17.06	0.80	20

* Error in terms of s.e.m. over N technical replicates.

Supplementary Table 4: ***Validated Primary Antibodies and fluorescent labels for Pre and Post MAGNIFY Staining***

Host	Target	Vendor	Cat. Number
Chicken	GFAP	Abcam	ab4674
Chicken	GFP	Abcam	ab13970
Chicken	NeuN	Millipore	ABN91
Chicken	Tyrosine Hydroxalase	Abcam	ab76442
Chicken	Vimentin	Abcam	ab24525
Goat	PSD-95	Abcam	ab12093
Goat	CD4	R&D Systems	AF-379-SP
Goat	Talin-1	Novus	AF5456-SP
Mouse	Anti-Actin, α-Smooth Muscle	Sigma Aldrich	A5228
Mouse	α-Tubulin	Sigma Aldrich	T6199
Mouse	Bassoon	Abcam	ab82958
Mouse	Cytokeratin Pan Type I/II	Invitrogen	MA5-13156
Mouse	Synaptophysin	Invitrogen	MA1-213
Mouse	CD11c	Abcam	ab11029
Mouse	CD4	Origene	UM870010
Mouse	CD8	Invitrogen	MA1-80231
Mouse	CD8a (Alexa 488)	Invitrogen	53-0008-82
Mouse	VDACI	Abcam	ab14734
Rabbit	α-Tubulin	Proteintech	11224-1-AP
Rabbit	ACTN4	Sigma Aldrich	HPA001873
Rabbit	Alpha-Internexin (INA)	Sigma Aldrich	HPA008057
Rabbit	Amyloid Precursor Protein (APP)	Sigma Aldrich	HPA001462
Rabbit	Anti-ATPase Inhibitory Factor 1 (ATPIF1)	Millipore	ABC137
Rabbit	Anti-ATPase Inhibitory Factor 1 (ATPIF1)	Proteintech	12067-1-AP
Rabbit	GABRA1	Proteintech	12410-1-AP

Rabbit	GABRB1	Proteintech	20183-1-AP
Rabbit	GluR2	Proteintech	11994-1-AP
Rabbit	Synaptophysin	Proteintech	17785-1-AP
Rabbit	CCR5	Proteintech	17476-1-AP
Rabbit	CD45	Abcam	ab10558
Rabbit	IL2	Proteintech	26156-1-AP
Rabbit	IL-6	Proteintech	21865-1-AP
Rabbit	TCR alpha Antibody	Novus	NBP2-52684
	Wheat Germ Agglutinin (WGA) CF555	Biotium	29076
	Wheat Germ Agglutinin (WGA) CF640R	Biotium	29026
	NHS-ATTO-488	Sigma Aldrich	41698
	NHS-ATTO-532	Sigma Aldrich	88793
	Succinimidyl Ester (NHS) CF 555	Biotium	92130
	Cy3 NHS ester (non-sulfonated)	Glpbio Tech.	GC12618-25
	Vybrant™ DiD cell-labeling solution	Invitrogen	V-22887
	Vybrant™ DiO cell-labeling solution	Invitrogen	V-22886
	Vybrant™ DiI cell-labeling solution	Invitrogen	V-22885
	Lycopersicon Esculentum (Tomato) Lectin (LEL)	Vector Labs	DL-1174-1

Supplementary Table 5: ***Secondary antibodies used for pre- and post-MAGNIFY staining.***

Reactivity	Host	Conjugate	Vendor	Catalog Number
Rabbit	Goat	DyLight550	Invitrogen	SA5-10033
Rabbit	Goat	CF555	Biotium	20232
Rabbit	Goat	Alexa Fluor 488	Invitrogen	A11034
Rabbit	Goat	CF640R	Biotium	20202
Rabbit	Goat	Fab Fragment AF488	Jackson Immuno	111-547-003
Rabbit	Donkey	CF488A	Biotium	20015
Rabbit	Donkey	Fab Fragment AF488	Jackson Immuno	711-547-003
Mouse	Goat	Alexa Fluor 488	Invitrogen	A11001
Mouse	Goat	CF568	Biotium	20301
Mouse	Donkey	CF555	Biotium	20037
Mouse	Donkey	CF640R	Biotium	20177
Mouse	Donkey	Fab Fragment AF488	Jackson Immuno	715-547-003
Chicken	Goat	Alexa Fluor 488	Invitrogen	A11039
Chicken	Goat	DyLight 488	Invitrogen	SA5-10070
Chicken	Goat	DyLight 550	Invitrogen	SA5-10033
Chicken	Goat	CF488A	Biotium	20020
Chicken	Goat	CF555	Biotium	20034
Chicken	Goat	CF640	Biotium	20084
Goat	Donkey	CF647	Biotium	20829
Streptavidin		CF 640	Biotium	292037

Supplementary Table 6: ***Comparison of expansion factors for different hydrogel chemistries for different tissue types.***

Components Concentrations							Expansion Factor	
**DMAA**	**SA**	**AA**	**Bis**	**NaCl**	**PBS**	**Anchoring**	**Human Kidney**	**Mouse Brain**
4	34	10	0.01	1	1	Methacrolein	8.14	10.6
0	15	5	0.05	11.7	1	AcX	5.54	N/A
26.7	6.4	0	0	0	0	AcX	6.03	N/A

*PBS given in terms of 1x PBS concentration. N,N-Dimethylacrylamide (DMAA), Sodium acrylate (SA), Acrylamide (AA), N,N′-Methylenebisacrylamide (Bis), NaCl given in terms of w/v%.

Supplementary Table 7: ***Exploration of different hydrogel chemistries and their respective expansion factors of blank gels in water.***

Components Concentrations								
**DMAA**	**SA**	**AA**	**Bis**	**NaCl**	**PBS**	**APS**	**4HT**	**Ex Factor**
1.5	37	15	0.01	1	1	1:50	1:500	6.5 (n=2)
2	34	10	0.01	1	1	1:30	0	7.76
2	34	10	0.01	1	1	1:50	1:500	7.5 (n=2)
4	30	10	0.01	1	1	1:30	0	6.45
4	34	10	0.01	1	1	1:30	0	7.86
4	33	10	0.01	1	1	1:50	1:500	7.5 (n=2)
8	30	10	0.01	1	1	1:30	0	7.46
0	15	5	0.05	11.7	1	1:30	0	6.5
0	8.6	2.5	0.075	11.7	1	1:30	0	6

*PBS given in terms of 1x PBS concentration. N,N-Dimethylacrylamide (DMAA), Sodium acrylate (SA), Acrylamide (AA), N,N′-Methylenebisacrylamide (Bis), NaCl given in terms of w/v%.

Supplementary Table 8: ***Gelling Conditions for MAGNIFY Gel.***

Temp	APS	4HT	Ex Factor
45C	1:50	1:500	7
45C	1:40	1:500	8
45C	1:30	1:500	8
45C	1:50	1:250	7
45C	1:40	1:250	8
45C	1:30	1:250	7.5
37C	1:50	1:500	8
37C	1:40	1:500	8.5
37C	1:30	1:500	8
37C	1:50	1:250	7.5
37C	1:40	1:250	8
37C	1:30	1:250	7

Supplementary Table 9: ***Deformation testing of different gel chemistries***

Gel									
	**DMAA**	**SA**	**AA**	**Bis**	**NaCl**	**PBS**	**Deformation**	**EF**	**N**
**MAGNIFY**	4	34	10	0.01	1	1	0.03± 0.01	5.9±0.28	4
**TREx (Low Bis)**	0	11	14.5	0.005	0	1	N/A		
**TREx (Med. Bis)**	0	11	14.5	0.009	0	1	0.51± 0.10	6.6±0.10	4
**TREx (High Bis)**	0	11	14.5	0.015	0	1	0.28± 0.09	5.9±0.27	3
**ExPath**	0	15	5	0.05	11.7	1	0.04± 0.02	4.3±0.14	4

*Errors given in terms of s.e.m.

**PBS given in terms of 1x PBS concentration. N,N-Dimethylacrylamide (DMAA), Sodium acrylate (SA), Acrylamide (AA), N,N′-Methylenebisacrylamide (Bis), NaCl given in terms of w/v%.

Supplementary Table 10: ***Gelling Solution Recipe***

**Component**	**Stock Concentration ***	**Amount (mL)**	**Final Concentration**
N,N-dimethylacrylamide (DMAA)		0.416	4
Sodium acrylate (SA)	50	6.8	34
Acrylamide	66.7	1.499	10
N,N′-Methylenebisacrylamide	2	0.05	0.01
Sodium chloride	30	0.333	1
PBS	10x	1	1x
**Total Volume**		**10 mL**	
* Note All concentrations given in g/100 mL except PBS			

Supplementary Table 11: ***List of Chemicals and Reagents***

**Step**	**Reagent**	**Acronym**	**Vendor**	**Catalog Number**
**Gelling**	N,N-dimethylacrylamide	DMAA	Sigma Aldrich	274135
	Sodium acrylate	SA	AK Scientific	R624
	Sodium acrylate	SA	Santa Cruz Biotechnology	sc-236893B
	Acrylamide	AA	Sigma Aldrich	A8887
	N,N′-Methylenebisacrylamide	BIS	Sigma Aldrich	M7279
	4-hydroxy-TEMPO	4HT	Sigma Aldrich	176141
	Sodium chloride	NaCl	Sigma Aldrich	S6191
	Phosphate Buffered Saline, 10x Solution	PBS	Fischer Scientifc	BP399-1
	Acryloyl-X, SE	AcX	Invitrogen	A20770
	Ammonium persulfate	APS	Sigma Aldrich	A3678
	N,N,N′,N′-Tetramethylethylenediamine	TEMED	Sigma Aldrich	T9281
	Methacrolein		Sigma Aldrich	133035
**Homogenizing**	Ethylenediaminetetraacetic acid 0.5 M	EDTA	VWR	BDH7830-1
	Triton X-100		Sigma Aldrich	T8787
	Tris Base		Fischer Scientifc	BP152-1
	Sodium chloride	NaCl	Sigma Aldrich	S6191
	Proteinase K (Molecular Biology Grade)	ProK	Thermo Scientific	EO0491
	Phosphate Buffered Saline, 10x Solution	PBS	Fischer Scientifc	BP399-1
	Sodium dodecyl sulfate	SDS	Sigma Aldrich	L3771
	Urea		Sigma Aldrich	U5378
	Glycine		Sigma Aldrich	G8898
**Other**	Sodium citrate tribasic dihydrate		Sigma Aldrich	C8532-1KG
	Xylenes		Sigma Aldrich	214736
	Ethanol		Pharmco	111000200
	SuperBlock Bloacking Buffer in PBS		Thermo Scientific	37515
	Heparin		Sigma Aldrich	H3393
	DAPI		Thermo	62248

Supplementary Table 12: ***DNA FISH probes design***

Name	Target sequence	Oligo sequence
AKT1-1	ATGTCGATCCTAGAGTCCAGAACGGGGACTTCC	ATGTCGATCCTAGAGTCCAGAACGGGGACTTCCATAATCGCTAGGCACCTGGATT
AKT1-2	GGAACGTGTTCAAAGAGGAGGAGACTCCGGACA	GGAACGTGTTCAAAGAGGAGGAGACTCCGGACAATAATCGCTAGGCACCTGGATT
AKT1-3	AATCGCCAGAGAAGTAGAGAGTGTGTTTGGCGGG	AATCGCCAGAGAAGTAGAGAGTGTGTTTGGCGGGATAATCGCTAGGCACCTGGATT
AKT1-4	ACCGGATCATCTTCACAACAGTCCCATTAACTAGACGC	ACCGGATCATCTTCACAACAGTCCCATTAACTAGACGCATAATCGCTAGGCACCTGGATT
AKT1-5	GGTCTCATTCTTCTCTGCCTTGGAGTCCGGGA	GGTCTCATTCTTCTCTGCCTTGGAGTCCGGGAATAATCGCTAGGCACCTGGATT
AKT1-6	AATGGAGAATGTAGTGGAGGAGTCACCCCAGTCAAG	AATGGAGAATGTAGTGGAGGAGTCACCCCAGTCAAGATAATCGCTAGGCACCTGGATT
AKT1-7	AACTGCAGCTTGGACCCCTGGAATGACACT	AACTGCAGCTTGGACCCCTGGAATGACACTATAATCGCTAGGCACCTGGATT
AKT1-8	GCACTTACAGCCACTGAGACTAGCTTAGGGACGG	GCACTTACAGCCACTGAGACTAGCTTAGGGACGGATAATCGCTAGGCACCTGGATT
AKT1-9	GAACTCAGATGTGACTGCTCCCTTCCTGCCC	GAACTCAGATGTGACTGCTCCCTTCCTGCCCATAATCGCTAGGCACCTGGATT
AKT1-10	ACCCTTCACTGGTTTCTCTTCATCCCTGTCTCTGC	ACCCTTCACTGGTTTCTCTTCATCCCTGTCTCTGCATAATCGCTAGGCACCTGGATT
AKT1-11	TCACTTGGTGCTTAAAAGTTGGCAGGACGCAAGT	TCACTTGGTGCTTAAAAGTTGGCAGGACGCAAGTATAATCGCTAGGCACCTGGATT
AKT1-12	TGGGTCTCAAGTGCAGGAATGACAGGACACC	TGGGTCTCAAGTGCAGGAATGACAGGACACCATAATCGCTAGGCACCTGGATT
AKT1-13	TTTTGTCGGAAACATCTGGCCACAGAGCACCT	TTTTGTCGGAAACATCTGGCCACAGAGCACCTATAATCGCTAGGCACCTGGATT
AKT1-14	GGGGTGTTCTTGAAGAGAGGACTCTGCTTTCCCT	GGGGTGTTCTTGAAGAGAGGACTCTGCTTTCCCTATAATCGCTAGGCACCTGGATT
AKT1-15	GGCCTCCTTGGAGACAGCCAGTGCAAAATAAGC	GGCCTCCTTGGAGACAGCCAGTGCAAAATAAGCATAATCGCTAGGCACCTGGATT
AKT1-16	AAGAGGTCCAACCACTTCAAGAACAGCCCGC	AAGAGGTCCAACCACTTCAAGAACAGCCCGCATAATCGCTAGGCACCTGGATT
AKT1-17	CCCTCTTACCCTCTGCTTCCTCCCTGAATTCCT	CCCTCTTACCCTCTGCTTCCTCCCTGAATTCCTATAATCGCTAGGCACCTGGATT
AKT1-18	GGATGGTGGACAGATACCAGGAACTCTTCCTCGG	GGATGGTGGACAGATACCAGGAACTCTTCCTCGGATAATCGCTAGGCACCTGGATT
AKT1-19	TACAAAGTCTGAGCTGGGAGGGAGCAGAGGT	TACAAAGTCTGAGCTGGGAGGGAGCAGAGGTATAATCGCTAGGCACCTGGATT
AKT1-20	GGGGAGAGAGTCCTTCTCTTGGTCAGCCCC	GGGGAGAGAGTCCTTCTCTTGGTCAGCCCCATAATCGCTAGGCACCTGGATT
AKT1-21	AGAAAGGGGTCTGTGTCCCACTTACTCATTCCATACC	AGAAAGGGGTCTGTGTCCCACTTACTCATTCCATACCATAATCGCTAGGCACCTGGATT
AKT1-22	GGGGACATCCAGAGGTCTTTGAGTCCAGCC	GGGGACATCCAGAGGTCTTTGAGTCCAGCCATAATCGCTAGGCACCTGGATT
AKT1-23	GCTTTCCATCCTGCTAAGTACTTGGGGCATTTCCC	GCTTTCCATCCTGCTAAGTACTTGGGGCATTTCCCATAATCGCTAGGCACCTGGATT
AKT1-24	TCAGAGGGGAAATGAGGAAGCCATGCAGGATCA	TCAGAGGGGAAATGAGGAAGCCATGCAGGATCAATAATCGCTAGGCACCTGGATT
AKT1-25	AGGAACACCATGGACAGGGAGAGCAAACGG	AGGAACACCATGGACAGGGAGAGCAAACGGATAATCGCTAGGCACCTGGATT
AKT1-26	AGCCTAGAATAAGGAGAGGCCCAGGTCCAGG	AGCCTAGAATAAGGAGAGGCCCAGGTCCAGGATAATCGCTAGGCACCTGGATT

AKT1-27	TAGCCAGGCGTGGCCTCACATTCAGCT	TAGCCAGGCGTGGCCTCACATTCAGCTATAATCGCTAGGCACCTGGATT
AKT1-28	GTAGTAGCCCCAGGGTCTGTGAGTGCCTG	GTAGTAGCCCCAGGGTCTGTGAGTGCCTGATAATCGCTAGGCACCTGGATT
AKT1-29	TTTTGCTCCTCTGTCCTGCTAGGGTGGGC	TTTTGCTCCTCTGTCCTGCTAGGGTGGGCATAATCGCTAGGCACCTGGATT
AKT1-30	AAGTCACTCTCTTCTGCCCCAGGCGGAATC	AAGTCACTCTCTTCTGCCCCAGGCGGAATCATAATCGCTAGGCACCTGGATT
AKT1-31	GTTTCACATCTGGTAGTGGGGAGACCCCAAACAC	GTTTCACATCTGGTAGTGGGGAGACCCCAAACACATAATCGCTAGGCACCTGGATT
AKT1-32	AAAAGTAGGTGTCACAAGATGGGGCATTGTGGGATG	AAAAGTAGGTGTCACAAGATGGGGCATTGTGGGATGATAATCGCTAGGCACCTGGATT
AKT1-33	AGAGCAAGGTCATTGAGCTCCTTGGGCCT	AGAGCAAGGTCATTGAGCTCCTTGGGCCTATAATCGCTAGGCACCTGGATT
AKT1-34	CAGTTTCCCCATCTACACCAGGGAGCGGT	CAGTTTCCCCATCTACACCAGGGAGCGGTATAATCGCTAGGCACCTGGATT
AKT1-35	ATAGCCCCTCTTGTTGCCGAGAGCAGGT	ATAGCCCCTCTTGTTGCCGAGAGCAGGTATAATCGCTAGGCACCTGGATT
AKT1-36	TCCTTGGGAGGTGAGCGTCATCTCTGGGA	TCCTTGGGAGGTGAGCGTCATCTCTGGGAATAATCGCTAGGCACCTGGATT
AKT1-37	TGTGTGTGCTCTGAGTCAGAGGAGCTTCAGGG	TGTGTGTGCTCTGAGTCAGAGGAGCTTCAGGGATAATCGCTAGGCACCTGGATT
AKT1-38	GGCCTCTGGGTCTGCACATCTAACAGGGAG	GGCCTCTGGGTCTGCACATCTAACAGGGAGATAATCGCTAGGCACCTGGATT
AKT1-39	CTTTGGCCAGAGGCTGTGTGGCTCTTTTGAGG	CTTTGGCCAGAGGCTGTGTGGCTCTTTTGAGGATAATCGCTAGGCACCTGGATT
AKT1-40	TACTTTCCCCAAACAGCTTGCTCACCTTGCCC	TACTTTCCCCAAACAGCTTGCTCACCTTGCCCATAATCGCTAGGCACCTGGATT
AKT1-41	TTCCTACCCCAGTGTGTTACTTCGCTTTGGTGAGC	TTCCTACCCCAGTGTGTTACTTCGCTTTGGTGAGCATAATCGCTAGGCACCTGGATT
AKT1-42	AAAACAACAAAAGAGGAAGCCGAGGTGGCCTTGA	AAAACAACAAAAGAGGAAGCCGAGGTGGCCTTGAATAATCGCTAGGCACCTGGATT
AKT1-43	TGGAATGATTCCTGTGCTGGGGCCTAGACC	TGGAATGATTCCTGTGCTGGGGCCTAGACCATAATCGCTAGGCACCTGGATT
AKT1-44	CCCGTACACCTTCCACTCTCAGGAGAAGCCT	CCCGTACACCTTCCACTCTCAGGAGAAGCCTATAATCGCTAGGCACCTGGATT
AKT1-45	TGCCGCCTTTAGGTGTGTTTTCCTTGAACCTTAAGG	TGCCGCCTTTAGGTGTGTTTTCCTTGAACCTTAAGGATAATCGCTAGGCACCTGGATT
AKT1-46	AGGAGAATGGAAAGCTGAGACCCAGGTGCTTCC	AGGAGAATGGAAAGCTGAGACCCAGGTGCTTCCATAATCGCTAGGCACCTGGATT
AKT1-47	ATGGTGGAGCGGAAGGAGGTGAAGAATTTGCA	ATGGTGGAGCGGAAGGAGGTGAAGAATTTGCAATAATCGCTAGGCACCTGGATT
AKT1-48	GTTTGTGTCAAGTTACAACCCCTGCCTTGGCG	GTTTGTGTCAAGTTACAACCCCTGCCTTGGCGATAATCGCTAGGCACCTGGATT
AKT1-49	ACTTTGAGAGAATGTTCAGGGAGCTGAGGACGTGG	ACTTTGAGAGAATGTTCAGGGAGCTGAGGACGTGGATAATCGCTAGGCACCTGGATT
AKT1-50	CCTGGGGACCCTCAGGATAGTGAAGGAGAAGAC	CCTGGGGACCCTCAGGATAGTGAAGGAGAAGACATAATCGCTAGGCACCTGGATT
AKT1-51	CCCCGTGGCTCCCTGGAAAGTAGAATGCC	CCCCGTGGCTCCCTGGAAAGTAGAATGCCATAATCGCTAGGCACCTGGATT
AKT1-52	AGACCTCTTCCCAGCCTCCTGTCCATGGT	AGACCTCTTCCCAGCCTCCTGTCCATGGTATAATCGCTAGGCACCTGGATT
AKT1-53	AGAGATGAGAGACATGTGACGCTCCCTGCTCC	AGAGATGAGAGACATGTGACGCTCCCTGCTCCATAATCGCTAGGCACCTGGATT
AKT1-54	GCCCTGCTGTGGCTTTATTCCTTACCTGTAGCG	GCCCTGCTGTGGCTTTATTCCTTACCTGTAGCGATAATCGCTAGGCACCTGGATT

AKT1-55	GTTGGGCATGCAGGTGTGACTGAGTGGC	GTTGGGCATGCAGGTGTGACTGAGTGGCATAATCGCTAGGCACCTGGATT
AKT1-56	CTTTCTTTGAGGCCTGCGGTCCTCCGACT	CTTTCTTTGAGGCCTGCGGTCCTCCGACTATAATCGCTAGGCACCTGGATT
AKT1-57	CTGAAGCTTCTAGTTGGGAGGGGCAGAGGC	CTGAAGCTTCTAGTTGGGAGGGGCAGAGGCATAATCGCTAGGCACCTGGATT
AKT1-58	CTCGTAACCATGCAGGAGACTCACTGTGACTTGTCC	CTCGTAACCATGCAGGAGACTCACTGTGACTTGTCCATAATCGCTAGGCACCTGGATT
AKT1-59	TTCAGATTAGGCCATAGGTGAGTGGCCTGGAGC	TTCAGATTAGGCCATAGGTGAGTGGCCTGGAGCATAATCGCTAGGCACCTGGATT
AKT1-60	GTGTATTCCAGTGTGGCTGTGTCCCAGGTGT	GTGTATTCCAGTGTGGCTGTGTCCCAGGTGTATAATCGCTAGGCACCTGGATT
AKT1-61	ATGTATGTGTGCCCCAGGTGTGGCTGTGC	ATGTATGTGTGCCCCAGGTGTGGCTGTGCATAATCGCTAGGCACCTGGATT
AKT1-62	TGGGTGTGCTCCAGGTGGCTGTATTTCCC	TGGGTGTGCTCCAGGTGGCTGTATTTCCCATAATCGCTAGGCACCTGGATT
AKT1-63	AAAAGCAGGACATTTCTACACTAGGTGGGACAAGCAGG	AAAAGCAGGACATTTCTACACTAGGTGGGACAAGCAGGATAATCGCTAGGCACCTGGATT
AKT1-64	ATCTCCATACCCCTCATCCTCTGAGGCCTGG	ATCTCCATACCCCTCATCCTCTGAGGCCTGGATAATCGCTAGGCACCTGGATT
AKT1-65	CTGAGTGTATGTGGCCAGACCAGGTCAAGTGG	CTGAGTGTATGTGGCCAGACCAGGTCAAGTGGATAATCGCTAGGCACCTGGATT
AKT1-66	AGTTCCAGGTCACTGTGTTTGGGGACGATTCTCC	AGTTCCAGGTCACTGTGTTTGGGGACGATTCTCCATAATCGCTAGGCACCTGGATT
AKT1-67	CACCTTGCTCACCTTTCAAACACTCCTTGGCACC	CACCTTGCTCACCTTTCAAACACTCCTTGGCACCATAATCGCTAGGCACCTGGATT
AKT1-68	GGGTGACTTGTTCCTGCTGAGTTAGGGCTTCTG	GGGTGACTTGTTCCTGCTGAGTTAGGGCTTCTGATAATCGCTAGGCACCTGGATT
AKT1-69	AGATTGTGTCAGCCCTGGACTACCTGCACTCG	AGATTGTGTCAGCCCTGGACTACCTGCACTCGATAATCGCTAGGCACCTGGATT
AKT1-70	CCTTGCTTTCAGGGCTGCTCAAGAAGGACCC	CCTTGCTTTCAGGGCTGCTCAAGAAGGACCCATAATCGCTAGGCACCTGGATT
AKT1-71	GCGGTACCGACACTGTGGCCTTGTTTCC	GCGGTACCGACACTGTGGCCTTGTTTCCATAATCGCTAGGCACCTGGATT
AKT1-72	CATGCAGCATCGCTTCTTTGCCGGTATCGT	CATGCAGCATCGCTTCTTTGCCGGTATCGTATAATCGCTAGGCACCTGGATT
AKT1-73	CGCATGCTCCCCACATATCCACACTCACGC	CGCATGCTCCCCACATATCCACACTCACGCATAATCGCTAGGCACCTGGATT
AKT1-74	AGCCTGCTGCAGTCCTGGTACAAGGAGG	AGCCTGCTGCAGTCCTGGTACAAGGAGGATAATCGCTAGGCACCTGGATT
AKT1-75	TCATCTTTCAGGGACCCTAGGAGCCCTGGC	TCATCTTTCAGGGACCCTAGGAGCCCTGGCATAATCGCTAGGCACCTGGATT
AKT1-76	GCTGGGTTCGGAAGCCTGCACTCTGAGA	GCTGGGTTCGGAAGCCTGCACTCTGAGAATAATCGCTAGGCACCTGGATT
AKT1-77	GCCCAGCCTTGACCAGAGACCTTGCTAATTGA	GCCCAGCCTTGACCAGAGACCTTGCTAATTGAATAATCGCTAGGCACCTGGATT
AKT1-78	AGATCCAGGTGCTTTGAAGGTCTTGAGCACACTTG	AGATCCAGGTGCTTTGAAGGTCTTGAGCACACTTGATAATCGCTAGGCACCTGGATT
AKT1-79	AAAAGGTTCCTTTTGGAGAGTGCCAATGATCAGGGTG	AAAAGGTTCCTTTTGGAGAGTGCCAATGATCAGGGTGATAATCGCTAGGCACCTGGATT
AKT1-80	TGGGCTGTCTGTCACCAGCTATCTGTCATCTCT	TGGGCTGTCTGTCACCAGCTATCTGTCATCTCTATAATCGCTAGGCACCTGGATT
AKT1-81	TTTTGGCTCACTTTGCTGGGTGGAAGAGTGGG	TTTTGGCTCACTTTGCTGGGTGGAAGAGTGGGATAATCGCTAGGCACCTGGATT
AKT1-82	ATGAAGGAGACCATTGGACCACGTGGCCA	ATGAAGGAGACCATTGGACCACGTGGCCAATAATCGCTAGGCACCTGGATT

AKT1-83	GGTAGGGCAGTGAATGAGACAGACCACCAGGA	GGTAGGGCAGTGAATGAGACAGACCACCAGGAATAATCGCTAGGCACCTGGATT
AKT1-84	AGGACAGATAGGGCAGGGCCCAGAGTTGG	AGGACAGATAGGGCAGGGCCCAGAGTTGGATAATCGCTAGGCACCTGGATT
AKT1-85	CTGGCGATGGCAATTGGCCCCTCTCTAAGG	CTGGCGATGGCAATTGGCCCCTCTCTAAGGATAATCGCTAGGCACCTGGATT
AKT1-86	CCAAGAACAGCCACATCTGGAGCAGCCC	CCAAGAACAGCCACATCTGGAGCAGCCCATAATCGCTAGGCACCTGGATT
AKT1-87	CCCCTCTGACAATATAGGATTGCCTTAGGGCCATTTCT	CCCCTCTGACAATATAGGATTGCCTTAGGGCCATTTCTATAATCGCTAGGCACCTGGATT
AKT1-88	CTCTGAGAATTTCCCGACCCTCCTAGCAGCCC	CTCTGAGAATTTCCCGACCCTCCTAGCAGCCCATAATCGCTAGGCACCTGGATT
AKT1-89	GGGGAAAGTGTTCTAGGGCAAAGAAGCATTGAGAAGAT GT	GGGGAAAGTGTTCTAGGGCAAAGAAGCATTGAGAAGATGTATAATCGCTAGGCACCTGGA TT
AKT1-90	TTTTGGTGAGAGAGTTTAGGTAAGGTACCCAGAGCCATGT	TTTTGGTGAGAGAGTTTAGGTAAGGTACCCAGAGCCATGTATAATCGCTAGGCACCTGGAT T
AKT1-91	ATCAGGCAGTAAGTCACTGAACCACGATTTGAATTCAGCC	ATCAGGCAGTAAGTCACTGAACCACGATTTGAATTCAGCCATAATCGCTAGGCACCTGGATT
AKT1-92	TTTCACCGGATGGACTGCATCTTTATTTCCTAAACCTGGC	TTTCACCGGATGGACTGCATCTTTATTTCCTAAACCTGGCATAATCGCTAGGCACCTGGATT
AKT1-93	TCTTTCACAAAAGGGTTATTATCAACTGTGGGCCTCTGGA	TCTTTCACAAAAGGGTTATTATCAACTGTGGGCCTCTGGAATAATCGCTAGGCACCTGGATT
AKT1-94	TCCAGACTTGTTCCCTCCTAAGTTCTAGTGATCTCATGCC	TCCAGACTTGTTCCCTCCTAAGTTCTAGTGATCTCATGCCATAATCGCTAGGCACCTGGATT
AKT1-95	CCCCTCAGATGAAGCAGTTGCCTAATTAGTAAGTTCCCT	CCCCTCAGATGAAGCAGTTGCCTAATTAGTAAGTTCCCTATAATCGCTAGGCACCTGGATT
AKT1-96	CATAGACCATGAACGAGTTTGAGTACCTGAAGCTGCTGG	CATAGACCATGAACGAGTTTGAGTACCTGAAGCTGCTGGATAATCGCTAGGCACCTGGATT
AKT1-97	TCACCTTATAGTCACCCTTCATCCTGGGTCATTGAGAGT	TCACCTTATAGTCACCCTTCATCCTGGGTCATTGAGAGTATAATCGCTAGGCACCTGGATT
AKT1-98	AAAATAAAGTTATCACCTCCTGGTAGCAGGGAGGGTCTCT	AAAATAAAGTTATCACCTCCTGGTAGCAGGGAGGGTCTCTATAATCGCTAGGCACCTGGAT T
AKT1-99	TACACGAAACATGACACAGTAAATGATGTTCCGAGGGTG A	TACACGAAACATGACACAGTAAATGATGTTCCGAGGGTGAATAATCGCTAGGCACCTGGAT T
AKT1-100	AGCATTGCTGAAAGTAACCAAGGTATGGAAATGGTCAGG C	AGCATTGCTGAAAGTAACCAAGGTATGGAAATGGTCAGGCATAATCGCTAGGCACCTGGAT T
AKT1_488	/5Alex488N/AATCCAGGTGCCTAGCGATT	
CDH1-1	GCCCTCTCTTGGTTACTGGGCTGTGGCT	GCCCTCTCTTGGTTACTGGGCTGTGGCTTATGTGCGGCATTGACTAAGAG
CDH1-2	AGAGCAAGGCAGGGGCTAGAAACAAGCTTGT	AGAGCAAGGCAGGGGCTAGAAACAAGCTTGTTATGTGCGGCATTGACTAAGAG
CDH1-3	ATCTGAGCATGCGTCTGGGGTGTCCCA	ATCTGAGCATGCGTCTGGGGTGTCCCATATGTGCGGCATTGACTAAGAG
CDH1-4	GGGGACCCTGGGACTGGTGATTTAGTGGG	GGGGACCCTGGGACTGGTGATTTAGTGGGTATGTGCGGCATTGACTAAGAG
CDH1-5	TCCTTTGCTTGCTCTGCAATCTGGCCTTGG	TCCTTTGCTTGCTCTGCAATCTGGCCTTGGTATGTGCGGCATTGACTAAGAG
CDH1-6	AAAGAAGACCAGTGGGCCGCCCTCCT	AAAGAAGACCAGTGGGCCGCCCTCCTTATGTGCGGCATTGACTAAGAG
CDH1-7	AAGGCCATGTAAAGAAGGCAGGCTGCTGC	AAGGCCATGTAAAGAAGGCAGGCTGCTGCTATGTGCGGCATTGACTAAGAG

CDH1-8	GCACCTGTCCCAATCCAATCCTTGCTGGG	GCACCTGTCCCAATCCAATCCTTGCTGGGTATGTGCGGCATTGACTAAGAG
CDH1-9	AGGCTAGACCCTGAGGTTACGGCAGATTGGA	AGGCTAGACCCTGAGGTTACGGCAGATTGGATATGTGCGGCATTGACTAAGAG
CDH1-10	GGGCAGGCAGGGAGATCTCAAACCTGAGT	GGGCAGGCAGGGAGATCTCAAACCTGAGTTATGTGCGGCATTGACTAAGAG
CDH1-11	GCTCCCTCTCACCCAGCAAACCAGCC	GCTCCCTCTCACCCAGCAAACCAGCCTATGTGCGGCATTGACTAAGAG
CDH1-12	GGGTGGACCGGAACGGGTTTGTTGTGG	GGGTGGACCGGAACGGGTTTGTTGTGGTATGTGCGGCATTGACTAAGAG
CDH1-13	CAGAGGTTGCACTGCTTGCCACCAAGTCAC	CAGAGGTTGCACTGCTTGCCACCAAGTCACTATGTGCGGCATTGACTAAGAG
CDH1-14	GCTCAGGACCCCGTGGGATAATTGGCCA	GCTCAGGACCCCGTGGGATAATTGGCCATATGTGCGGCATTGACTAAGAG
CDH1-15	TTTGCTTAAATGTCACTCCCGCCCTCAGGGA	TTTGCTTAAATGTCACTCCCGCCCTCAGGGATATGTGCGGCATTGACTAAGAG
CDH1-16	AAAATTATTTGGCAGGGGCAGGGCACGGT	AAAATTATTTGGCAGGGGCAGGGCACGGTTATGTGCGGCATTGACTAAGAG
CDH1-17	AGTGGGAGAGCCTGGCCGTATGTCTGG	AGTGGGAGAGCCTGGCCGTATGTCTGGTATGTGCGGCATTGACTAAGAG
CDH1-18	CAAGCCCTCTGCTAGGCATTGGGGATGC	CAAGCCCTCTGCTAGGCATTGGGGATGCTATGTGCGGCATTGACTAAGAG
CDH1-19	GCCCTGAGTCACCGACATTGAGAGGCCT	GCCCTGAGTCACCGACATTGAGAGGCCTTATGTGCGGCATTGACTAAGAG
CDH1-20	TGCTTCCAGTGCCTGGTACGTAGCAACCA	TGCTTCCAGTGCCTGGTACGTAGCAACCATATGTGCGGCATTGACTAAGAG
CDH1-21	GGGGCGGGTAGTGTAAGTGTCAAAGGAGCC	GGGGCGGGTAGTGTAAGTGTCAAAGGAGCCTATGTGCGGCATTGACTAAGAG
CDH1-22	TCATGCCAAGGTCCACCCATGCAGCC	TCATGCCAAGGTCCACCCATGCAGCCTATGTGCGGCATTGACTAAGAG
CDH1-23	TTCCGCACAGTGTGTGAGTGCCTTCTGTGT	TTCCGCACAGTGTGTGAGTGCCTTCTGTGTTATGTGCGGCATTGACTAAGAG
CDH1-24	GGGGATGCCCCAAGCCTAGAGAGGTGG	GGGGATGCCCCAAGCCTAGAGAGGTGGTATGTGCGGCATTGACTAAGAG
CDH1-25	TGGCAGGACTGCAGAAGGACAATAGTGTGGC	TGGCAGGACTGCAGAAGGACAATAGTGTGGCTATGTGCGGCATTGACTAAGAG
CDH1-26	TGGTTGGGTGAGGCCCTTTTGGCCTG	TGGTTGGGTGAGGCCCTTTTGGCCTGTATGTGCGGCATTGACTAAGAG
CDH1-27	AGGGGAGGGCGTTCTAGATGGAGGGAACA	AGGGGAGGGCGTTCTAGATGGAGGGAACATATGTGCGGCATTGACTAAGAG
CDH1-28	CCTCTGGTCCTTTAGCCCAGATCCACCGG	CCTCTGGTCCTTTAGCCCAGATCCACCGGTATGTGCGGCATTGACTAAGAG
CDH1-29	TTTCCAATGGCGCTGCTGGCTCTGGC	TTTCCAATGGCGCTGCTGGCTCTGGCTATGTGCGGCATTGACTAAGAG
CDH1-30	CCCCAGCATTAAGCCTGGCATGTAGTTGCC	CCCCAGCATTAAGCCTGGCATGTAGTTGCCTATGTGCGGCATTGACTAAGAG
CDH1-31	GGGAGGATTAGGCTGCAGGGTTACCCCA	GGGAGGATTAGGCTGCAGGGTTACCCCATATGTGCGGCATTGACTAAGAG
CDH1-32	CAGGAGCCTTGGGAGTGGGTTTACACCTCAA	CAGGAGCCTTGGGAGTGGGTTTACACCTCAATATGTGCGGCATTGACTAAGAG
CDH1-33	AGCAACTCAGTGGTGGAGGAGGGATTCCAGT	AGCAACTCAGTGGTGGAGGAGGGATTCCAGTTATGTGCGGCATTGACTAAGAG
CDH1-34	TTCCCTTCCTTGAGAAAACTGGCCCAGGCT	TTCCCTTCCTTGAGAAAACTGGCCCAGGCTTATGTGCGGCATTGACTAAGAG
CDH1-35	CTGGGCCTGTCTTCCACAAGACATCGCCT	CTGGGCCTGTCTTCCACAAGACATCGCCTTATGTGCGGCATTGACTAAGAG

CDH1-36	GCAGCACATAGAGGGCCCTTAGGGGATGT	GCAGCACATAGAGGGCCCTTAGGGGATGTTATGTGCGGCATTGACTAAGAG
CDH1-37	TTCAGGTGGGTACCAGCCAGTTGACATCCAG	TTCAGGTGGGTACCAGCCAGTTGACATCCAGTATGTGCGGCATTGACTAAGAG
CDH1-38	ACCAGCCTCGCACCTTCAGGGAACGT	ACCAGCCTCGCACCTTCAGGGAACGTTATGTGCGGCATTGACTAAGAG
CDH1-39	AGTGAAACGGGGTAGGGAGTGCCTGGG	AGTGAAACGGGGTAGGGAGTGCCTGGGTATGTGCGGCATTGACTAAGAG
CDH1-40	GTAGTGGTGCAGGGCCCCTTTAGCTGAGG	GTAGTGGTGCAGGGCCCCTTTAGCTGAGGTATGTGCGGCATTGACTAAGAG
CDH1-41	CCATCAGCTTCCACCGAGACGAAGTGATGCA	CCATCAGCTTCCACCGAGACGAAGTGATGCATATGTGCGGCATTGACTAAGAG
CDH1-42	AACCAGACCGTGCAGCCAACTCCTGC	AACCAGACCGTGCAGCCAACTCCTGCTATGTGCGGCATTGACTAAGAG
CDH1-43	AGTCCCAGGCAACTCAGACCTTCCCTGG	AGTCCCAGGCAACTCAGACCTTCCCTGGTATGTGCGGCATTGACTAAGAG
CDH1-44	TGGAGAAGGTGGGTGTTTTGTGTGTTCCCGT	TGGAGAAGGTGGGTGTTTTGTGTGTTCCCGTTATGTGCGGCATTGACTAAGAG
CDH1-45	ATGAGCCACCGTGAATGGCCAGAAGCAC	ATGAGCCACCGTGAATGGCCAGAAGCACTATGTGCGGCATTGACTAAGAG
CDH1-46	TGGTGTTCTTGCCTTTGGTTTGCCTAAGGCC	TGGTGTTCTTGCCTTTGGTTTGCCTAAGGCCTATGTGCGGCATTGACTAAGAG
CDH1-47	CCAGTGATTCCACGTGGTCCTGCCCTATGT	CCAGTGATTCCACGTGGTCCTGCCCTATGTTATGTGCGGCATTGACTAAGAG
CDH1-48	GGTGGCCTTTCAGGTTTTGTCAGGGCCA	GGTGGCCTTTCAGGTTTTGTCAGGGCCATATGTGCGGCATTGACTAAGAG
CDH1-49	TGGTCAGACATCAACACCGCCTGCCTCA	TGGTCAGACATCAACACCGCCTGCCTCATATGTGCGGCATTGACTAAGAG
CDH1-50	GGCTTGACTGAGATGCCCCAGTGGCTTG	GGCTTGACTGAGATGCCCCAGTGGCTTGTATGTGCGGCATTGACTAAGAG
CDH1-51	TGTGTGCTGTTATAGCTACGTGGCCTTGGGC	TGTGTGCTGTTATAGCTACGTGGCCTTGGGCTATGTGCGGCATTGACTAAGAG
CDH1-52	GGGGTGTTGGAGGGAGGCTGGAGAATGA	GGGGTGTTGGAGGGAGGCTGGAGAATGATATGTGCGGCATTGACTAAGAG
CDH1-53	AAGGGAGGCAACTGAGGCCACAGTGTCT	AAGGGAGGCAACTGAGGCCACAGTGTCTTATGTGCGGCATTGACTAAGAG
CDH1-54	CAGCCACCACCCTGGATTGGCTTCCAAC	CAGCCACCACCCTGGATTGGCTTCCAACTATGTGCGGCATTGACTAAGAG
CDH1-55	TCCAGGGTGTTGGCTGAGCAGAGCAAGT	TCCAGGGTGTTGGCTGAGCAGAGCAAGTTATGTGCGGCATTGACTAAGAG
CDH1-56	GAGGCTTGTCATGGCCTGACCGAGGTTGT	GAGGCTTGTCATGGCCTGACCGAGGTTGTTATGTGCGGCATTGACTAAGAG
CDH1-57	TGCCTCCGCTTCATTCTCCACCCTCCT	TGCCTCCGCTTCATTCTCCACCCTCCTTATGTGCGGCATTGACTAAGAG
CDH1-58	CCACACACCCGCAGCACTCATTTTAACCCC	CCACACACCCGCAGCACTCATTTTAACCCCTATGTGCGGCATTGACTAAGAG
CDH1-59	GGGGCGTTCAGGAGGCTACAGCCTAGT	GGGGCGTTCAGGAGGCTACAGCCTAGTTATGTGCGGCATTGACTAAGAG
CDH1-60	CCCCTTCCCTCTCCCATCATGTGTGGGT	CCCCTTCCCTCTCCCATCATGTGTGGGTTATGTGCGGCATTGACTAAGAG
CDH1-61	ACCACTGCTGGAACAGGTGCCCCATCT	ACCACTGCTGGAACAGGTGCCCCATCTTATGTGCGGCATTGACTAAGAG
CDH1-62	GATTACAGGGTTGAGCCTGCACCTGACCCT	GATTACAGGGTTGAGCCTGCACCTGACCCTTATGTGCGGCATTGACTAAGAG
CDH1-63	TGGAATTTCTCTCCCTCCGCAAGCCTAGTGC	TGGAATTTCTCTCCCTCCGCAAGCCTAGTGCTATGTGCGGCATTGACTAAGAG

CDH1-64	GGGGCGAATGTTCCTTCAGAGGCTGACCT	GGGGCGAATGTTCCTTCAGAGGCTGACCTTATGTGCGGCATTGACTAAGAG
CDH1-65	AGGCTGGTCTTGACGGGACTTGGACTCAAG	AGGCTGGTCTTGACGGGACTTGGACTCAAGTATGTGCGGCATTGACTAAGAG
CDH1-66	GGGCCCAGGACTTGTTCTCCCACATCTCA	GGGCCCAGGACTTGTTCTCCCACATCTCATATGTGCGGCATTGACTAAGAG
CDH1-67	GCTGTGTTGTTACGCATGCAGCCACAGCT	GCTGTGTTGTTACGCATGCAGCCACAGCTTATGTGCGGCATTGACTAAGAG
CDH1-68	AACCCCAGCTTCTCCAGTCAAGGCAGCA	AACCCCAGCTTCTCCAGTCAAGGCAGCATATGTGCGGCATTGACTAAGAG
CDH1-69	GGCCCGCAGGAGCAAGAGCTAACCTTTCT	GGCCCGCAGGAGCAAGAGCTAACCTTTCTTATGTGCGGCATTGACTAAGAG
CDH1-70	AGCCAGTACGCCCTGCGACACTGACT	AGCCAGTACGCCCTGCGACACTGACTTATGTGCGGCATTGACTAAGAG
CDH1-71	AAGGGGACTTTGCTCCCTGCTTCCCATCC	AAGGGGACTTTGCTCCCTGCTTCCCATCCTATGTGCGGCATTGACTAAGAG
CDH1-72	TGGTGAGTCCTGGCGGCTAAGTAGCATCAGA	TGGTGAGTCCTGGCGGCTAAGTAGCATCAGATATGTGCGGCATTGACTAAGAG
CDH1-73	CCTTTGTATGCAGGGTGGGCAAAACACTGCT	CCTTTGTATGCAGGGTGGGCAAAACACTGCTTATGTGCGGCATTGACTAAGAG
CDH1-74	CTTGCCAGCGTGACAGTGAGCTTCCCAG	CTTGCCAGCGTGACAGTGAGCTTCCCAGTATGTGCGGCATTGACTAAGAG
CDH1-75	ATTTGCTAAGGCCACCCAGCAAGAACATGGC	ATTTGCTAAGGCCACCCAGCAAGAACATGGCTATGTGCGGCATTGACTAAGAG
CDH1-76	TTTTCTCTGGCAAGGGCAGGGCTATTGGCT	TTTTCTCTGGCAAGGGCAGGGCTATTGGCTTATGTGCGGCATTGACTAAGAG
CDH1-77	GGGGACAAATCAGCCCAAGTTGAGGGCTCT	GGGGACAAATCAGCCCAAGTTGAGGGCTCTTATGTGCGGCATTGACTAAGAG
CDH1-78	TGGATGGATGTCTGCCTGGGTTCTTGGCA	TGGATGGATGTCTGCCTGGGTTCTTGGCATATGTGCGGCATTGACTAAGAG
CDH1-79	AAAACTCAGCACAGTGAGTCCTTGCCAGGCA	AAAACTCAGCACAGTGAGTCCTTGCCAGGCATATGTGCGGCATTGACTAAGAG
CDH1-80	TTTTGCTCCGCCATCCTCAGCACATGACCT	TTTTGCTCCGCCATCCTCAGCACATGACCTTATGTGCGGCATTGACTAAGAG
CDH1-81	TGGGGACGCTGTCTGGCTAGGTTGGA	TGGGGACGCTGTCTGGCTAGGTTGGATATGTGCGGCATTGACTAAGAG
CDH1-82	CCATGTCCCCTCCTTTATCCCTCAGGGCAG	CCATGTCCCCTCCTTTATCCCTCAGGGCAGTATGTGCGGCATTGACTAAGAG
CDH1-83	TCACCACTGGGCTGGACCGAGAGGTC	TCACCACTGGGCTGGACCGAGAGGTCTATGTGCGGCATTGACTAAGAG
CDH1-84	CTACCGAACCCAGCGACATCTGAAGAGCCC	CTACCGAACCCAGCGACATCTGAAGAGCCCTATGTGCGGCATTGACTAAGAG
CDH1-85	GCTGCTGCCTCAGTGGACTCCAGGAGA	GCTGCTGCCTCAGTGGACTCCAGGAGATATGTGCGGCATTGACTAAGAG
CDH1-86	CCCCTGTCTGGTATGAGGGGTGCTCTGTG	CCCCTGTCTGGTATGAGGGGTGCTCTGTGTATGTGCGGCATTGACTAAGAG
CDH1-87	CCCCAGAGGATGACACCCGGGACAACG	CCCCAGAGGATGACACCCGGGACAACGTATGTGCGGCATTGACTAAGAG
CDH1-88	ACTCGTAACGACGTTGCACCAACCCTCATGA	ACTCGTAACGACGTTGCACCAACCCTCATGATATGTGCGGCATTGACTAAGAG
CDH1-89	ATGGTCCAGTGGCCCTCGGTGAGTCTTC	ATGGTCCAGTGGCCCTCGGTGAGTCTTCTATGTGCGGCATTGACTAAGAG
CDH1-90	TGGCAGCCCCACTCTGATCTATGGGGAC	TGGCAGCCCCACTCTGATCTATGGGGACTATGTGCGGCATTGACTAAGAG
CDH1-91	GGTGTGCCACAAGTCTGGGTGCATTGTCG	GGTGTGCCACAAGTCTGGGTGCATTGTCGTATGTGCGGCATTGACTAAGAG

CDH1-92	CGAGGACGACTAGGGGACTCGAGAGAGGC	CGAGGACGACTAGGGGACTCGAGAGAGGCTATGTGCGGCATTGACTAAGAG
CDH1-93	TGGCCTCAGTCAAAACGTGGAGAAAGAGGCC	TGGCCTCAGTCAAAACGTGGAGAAAGAGGCCTATGTGCGGCATTGACTAAGAG
CDH1--94	AGGAGTATTCCCGTTTTACAGTTGAGCAAACCGAGGC	AGGAGTATTCCCGTTTTACAGTTGAGCAAACCGAGGCTATGTGCGGCATTGACTAAGAG
CDH1--95	TAAGAGAGTGACGTCCACTTGCTCAGGGTCAGC	TAAGAGAGTGACGTCCACTTGCTCAGGGTCAGCTATGTGCGGCATTGACTAAGAG
CDH1--96	AGAGCAGATATCTGAACTGGGATCTGACTCCCAGGC	AGAGCAGATATCTGAACTGGGATCTGACTCCCAGGCTATGTGCGGCATTGACTAAGAG
CDH1--97	CCCATTAAGCACCATCTCTGGGATGACTGAAGAGCA	CCCATTAAGCACCATCTCTGGGATGACTGAAGAGCATATGTGCGGCATTGACTAAGAG
CDH1--98	CTGAGAGACTGTGCCTGGACCAAAACATCTTGATACAGG	CTGAGAGACTGTGCCTGGACCAAAACATCTTGATACAGGTATGTGCGGCATTGACTAAGAG
CDH1--99	AGCAAGGGAAGGGGCTAGTTTCTCTTCTGGTTTCA	AGCAAGGGAAGGGGCTAGTTTCTCTTCTGGTTTCATATGTGCGGCATTGACTAAGAG
CDH1--100	TTGGATATTTTGATGTCAGTGGGCATTGAGGAGTGGC	TTGGATATTTTGATGTCAGTGGGCATTGAGGAGTGGCTATGTGCGGCATTGACTAAGAG
CDH1--101	TTCTCGGGGAAGGCAAACTTGATCCAGTCTTGG	TTCTCGGGGAAGGCAAACTTGATCCAGTCTTGGTATGTGCGGCATTGACTAAGAG
CDH1--102	GTGCTAAGGCTGGTGACTTTGTTTTCCTGCACC	GTGCTAAGGCTGGTGACTTTGTTTTCCTGCACCTATGTGCGGCATTGACTAAGAG
CDH1--103	AAGATATTTGGTAGCACCAAGGGTGGGTTGGTCTGG	AAGATATTTGGTAGCACCAAGGGTGGGTTGGTCTGGTATGTGCGGCATTGACTAAGAG
CDH1--104	AATAGTGACACGTATTCACTTTTGCCTCAGACTCTGGGG	AATAGTGACACGTATTCACTTTTGCCTCAGACTCTGGGGTATGTGCGGCATTGACTAAGAG
CDH1--105	CCTCTGGTGTGGTTGGTTTCAGTTCCCTTTATGTTCAC	CCTCTGGTGTGGTTGGTTTCAGTTCCCTTTATGTTCACTATGTGCGGCATTGACTAAGAG
CDH1--106	TTTTGGACTGTGGGGTCTCCTTGTATCAGTCCAATGT	TTTTGGACTGTGGGGTCTCCTTGTATCAGTCCAATGTTATGTGCGGCATTGACTAAGAG
CDH1--107	GATTTTAAAAGGCCGCTAGGACTTGGGATGTGGCC	GATTTTAAAAGGCCGCTAGGACTTGGGATGTGGCCTATGTGCGGCATTGACTAAGAG
CDH1--108	TGACTCTCTTTGGACAGTGCACGGAAGTGAAGGG	TGACTCTCTTTGGACAGTGCACGGAAGTGAAGGGTATGTGCGGCATTGACTAAGAG
CDH1--109	ATGGATGTAACATTGGCACCAGGTTTAGACATTGGCTTC	ATGGATGTAACATTGGCACCAGGTTTAGACATTGGCTTCTATGTGCGGCATTGACTAAGAG
CDH1--110	TTTTGTGCACAGGCAGCTGTAGGGTCTTAACTCAG	TTTTGTGCACAGGCAGCTGTAGGGTCTTAACTCAGTATGTGCGGCATTGACTAAGAG
CDH1--111	GGGACTTTGGAGTGTTTGCTAGGGTCATTCCTCTGC	GGGACTTTGGAGTGTTTGCTAGGGTCATTCCTCTGCTATGTGCGGCATTGACTAAGAG
CDH1--112	ATGCCTGGCTAATAACCCAGTCCTTTAGAGTTGCTGC	ATGCCTGGCTAATAACCCAGTCCTTTAGAGTTGCTGCTATGTGCGGCATTGACTAAGAG

CDH1--113	AAAAGAAAAGCTGGCAGGCTTTCCCAGTGATTGTTTTCT	AAAAGAAAAGCTGGCAGGCTTTCCCAGTGATTGTTTTCTTATGTGCGGCATTGACTAAGAG
CDH1--114	AAACACAGGGCCTATATTTCAGTGTTGAGGTTTGGCTG	AAACACAGGGCCTATATTTCAGTGTTGAGGTTTGGCTGTATGTGCGGCATTGACTAAGAG
CDH1--115	TGAAGGATGTGCTGAACGTTTCAGAAAACAGTATGCAGC	TGAAGGATGTGCTGAACGTTTCAGAAAACAGTATGCAGCTATGTGCGGCATTGACTAAGAG
CDH1--116	AAAAGCAAACCCAGAAACCAGCTTTGGGATCACCT	AAAAGCAAACCCAGAAACCAGCTTTGGGATCACCTTATGTGCGGCATTGACTAAGAG
CDH1--117	CCCCTACCCCTCAAACTCATAAAAGTCACTTCCCCA	CCCCTACCCCTCAAACTCATAAAAGTCACTTCCCCATATGTGCGGCATTGACTAAGAG
CDH1--118	ACTCATGTCTGTGACAAAGGTTGATCTGAGCTTACTGGG	ACTCATGTCTGTGACAAAGGTTGATCTGAGCTTACTGGGTATGTGCGGCATTGACTAAGAG
CDH1--119	GGTTATGGAAATCCCTGTTTGCTCTCCAGGGCT	GGTTATGGAAATCCCTGTTTGCTCTCCAGGGCTTATGTGCGGCATTGACTAAGAG
CDH1--120	GTCTGTTACACATTCCCTTCCAAGGTAGCCCCAGA	GTCTGTTACACATTCCCTTCCAAGGTAGCCCCAGATATGTGCGGCATTGACTAAGAG
CDH1--121	AGAAGCTGCCCAAACCCTAAAATCCCCGAGAG	AGAAGCTGCCCAAACCCTAAAATCCCCGAGAGTATGTGCGGCATTGACTAAGAG
CDH1--122	TATCCTGCCTCAGCTCTATCTCCTTTGAGGGCAC	TATCCTGCCTCAGCTCTATCTCCTTTGAGGGCACTATGTGCGGCATTGACTAAGAG
CDH1--123	CGGAACATACATGCCAATGGGCAGGAATGTAAAGAGAG	CGGAACATACATGCCAATGGGCAGGAATGTAAAGAGAGTATGTGCGGCATTGACTAAGAG
CDH1--124	TTTTCCCTCTCAAACTCTATGGACTTGGTCCAGCCC	TTTTCCCTCTCAAACTCTATGGACTTGGTCCAGCCCTATGTGCGGCATTGACTAAGAG
CDH1--125	TTAAGGTTGATTGTAAATGGGCGGGGAAGAATTTGGTGG	TTAAGGTTGATTGTAAATGGGCGGGGAAGAATTTGGTGGTATGTGCGGCATTGACTAAGAG
CDH1--126	GTTAGAACCTTCCATTATGCTCTGTGTGTGCTGGGG	GTTAGAACCTTCCATTATGCTCTGTGTGTGCTGGGGTATGTGCGGCATTGACTAAGAG
CDH1--127	ATACTGTGTTCATAGCTTCCCTCTGAACAGCTGTGCA	ATACTGTGTTCATAGCTTCCCTCTGAACAGCTGTGCATATGTGCGGCATTGACTAAGAG
CDH1--128	CTCCAATCCTCCACTTCTGCCCAAGATTTGGGT	CTCCAATCCTCCACTTCTGCCCAAGATTTGGGTTATGTGCGGCATTGACTAAGAG
CDH1--129	AGCATGGAGACATCATAGGATGGTATGTCAAGCGTTCA	AGCATGGAGACATCATAGGATGGTATGTCAAGCGTTCATATGTGCGGCATTGACTAAGAG
CDH1--130	AAACCAGTTTCTTCACCCTGCCTGCTTCTGTGT	AAACCAGTTTCTTCACCCTGCCTGCTTCTGTGTTATGTGCGGCATTGACTAAGAG

CDH1--131	TAAGCAAACAGTGCACTGCAAACAGTGACTAAGCCT	TAAGCAAACAGTGCACTGCAAACAGTGACTAAGCCTTATGTGCGGCATTGACTAAGAG
CDH1--132	AAAATCAAATCACCTAGGGCACTTCATGCAGACTTGCC	AAAATCAAATCACCTAGGGCACTTCATGCAGACTTGCCTATGTGCGGCATTGACTAAGAG
CDH1--133	CTCTTGCAGATTCACTGAGAGCCAATCTCCAAGGGT	CTCTTGCAGATTCACTGAGAGCCAATCTCCAAGGGTTATGTGCGGCATTGACTAAGAG
CDH1--134	ATTGAGAACTCTATGGATGCAAGGAGGGGTGGATGG	ATTGAGAACTCTATGGATGCAAGGAGGGGTGGATGGTATGTGCGGCATTGACTAAGAG
CDH1--135	CCCCTACACTCAGGGAGATTCCAGAAAATCTCCAAGAGA	CCCCTACACTCAGGGAGATTCCAGAAAATCTCCAAGAGATATGTGCGGCATTGACTAAGAG
CDH1--136	GACCTCTCAGCAGGTAATTCCAGTAGGCTCCAAGA	GACCTCTCAGCAGGTAATTCCAGTAGGCTCCAAGATATGTGCGGCATTGACTAAGAG
CDH1--137	CTTGATGAGTAAGAGGGTGTCTTCCATGCAAGGAAAGGG	CTTGATGAGTAAGAGGGTGTCTTCCATGCAAGGAAAGGGTATGTGCGGCATTGACTAAGAG
CDH1--138	GTGGGTCATGGAGGGTTTTCTCTTTCTGGGCT	GTGGGTCATGGAGGGTTTTCTCTTTCTGGGCTTATGTGCGGCATTGACTAAGAG
CDH1--139	CACATCAGCGTTTAGAGACAGTCGGTTCCCACTG	CACATCAGCGTTTAGAGACAGTCGGTTCCCACTGTATGTGCGGCATTGACTAAGAG
CDH1--140	TCCCAGTGGAGGTTTTGAGAAAGAGGTTGGGAGA	TCCCAGTGGAGGTTTTGAGAAAGAGGTTGGGAGATATGTGCGGCATTGACTAAGAG
CDH1--141	ATGTTCCTTTGGACCAACACTGGAAACAGAGGAGAAAGA	ATGTTCCTTTGGACCAACACTGGAAACAGAGGAGAAAGATATGTGCGGCATTGACTAAGAG
CDH1--142	TTTCAAAGGGGTATTTGGGAATGTCAGCTCTGTGCTTG	TTTCAAAGGGGTATTTGGGAATGTCAGCTCTGTGCTTGTATGTGCGGCATTGACTAAGAG
CDH1--143	CGCTTGACAGTTAATCCTCCCTGTAGTCCAAGGTGG	CGCTTGACAGTTAATCCTCCCTGTAGTCCAAGGTGGTATGTGCGGCATTGACTAAGAG
CDH1--144	AAAACCAGGTCTCTCATCCTGTTCCCTCTACTACACTGT	AAAACCAGGTCTCTCATCCTGTTCCCTCTACTACACTGTTATGTGCGGCATTGACTAAGAG
CDH1--145	CAGTTTCAGCCGGTTTCCCTCTTGACCTTCACT	CAGTTTCAGCCGGTTTCCCTCTTGACCTTCACTTATGTGCGGCATTGACTAAGAG
CDH1--146	TTCAAGACTTGGGAGTGTAGGGACAGGGAAACCG	TTCAAGACTTGGGAGTGTAGGGACAGGGAAACCGTATGTGCGGCATTGACTAAGAG
CDH1--147	GCTCTTGCTGCTGAAAGAGGGAAAGTTACTACTGGGA	GCTCTTGCTGCTGAAAGAGGGAAAGTTACTACTGGGATATGTGCGGCATTGACTAAGAG
CDH1--148	TGTTCCACATGAGCAAGATCATAAACGGTGGCCA	TGTTCCACATGAGCAAGATCATAAACGGTGGCCATATGTGCGGCATTGACTAAGAG

CDH1--149	GATTCTGTGGTTTGGCATTCAGGTACGCCTGTACA	GATTCTGTGGTTTGGCATTCAGGTACGCCTGTACATATGTGCGGCATTGACTAAGAG
CDH1--150	CATGACGCCCAGCTGTTTCTCTATTCCCAAAAGAGAAG	CATGACGCCCAGCTGTTTCTCTATTCCCAAAAGAGAAGTATGTGCGGCATTGACTAAGAG
CDH1--151	TTTCAGCATGTCTTCACCTTCATTGTCGCAGCAGC	TTTCAGCATGTCTTCACCTTCATTGTCGCAGCAGCTATGTGCGGCATTGACTAAGAG
CDH1--152	TTCCTTCCTGGTCTCCCCACTTATTCCTTCCCC	TTCCTTCCTGGTCTCCCCACTTATTCCTTCCCCTATGTGCGGCATTGACTAAGAG
CDH1--153	TTCCTCCAAGCCAGTCTTTTCTCCATCCATCAGTCA	TTCCTCCAAGCCAGTCTTTTCTCCATCCATCAGTCATATGTGCGGCATTGACTAAGAG
CDH1--154	GATCTGTCCTTTGACTCATGTACCTGGGGTCCTCA	GATCTGTCCTTTGACTCATGTACCTGGGGTCCTCATATGTGCGGCATTGACTAAGAG
CDH1--155	TCTCTTCCCACTCTTCTGCATGCTCACTTTGCC	TCTCTTCCCACTCTTCTGCATGCTCACTTTGCCTATGTGCGGCATTGACTAAGAG
CDH1--156	AGCTCACTCTTTTCTGTCCTGAAAAGACTGCAAGCC	AGCTCACTCTTTTCTGTCCTGAAAAGACTGCAAGCCTATGTGCGGCATTGACTAAGAG
CDH1--157	CCCCTATTTATGATAGTAAACCCTGCCCTCAGCCCT	CCCCTATTTATGATAGTAAACCCTGCCCTCAGCCCTTATGTGCGGCATTGACTAAGAG
CDH1--158	AAAGACAGGGCTTTATGTATTAGCCACAGAGAAGGGAGG	AAAGACAGGGCTTTATGTATTAGCCACAGAGAAGGGAGGTATGTGCGGCATTGACTAAGAG
CDH1--159	CATTTCTACCCCACACTTCTGAGTGTAAGGAAGCACCA	CATTTCTACCCCACACTTCTGAGTGTAAGGAAGCACCATATGTGCGGCATTGACTAAGAG
CDH1--160	GATCAAAATCTAGGCAGATATCTGAGCCCGCCAGAGC	GATCAAAATCTAGGCAGATATCTGAGCCCGCCAGAGCTATGTGCGGCATTGACTAAGAG
CDH1--161	CCCTGGGTATCTCAGCATCCTTCAGACCCATGA	CCCTGGGTATCTCAGCATCCTTCAGACCCATGATATGTGCGGCATTGACTAAGAG
CDH1--162	AAAAGCAAGGCCTGCTTAATCAGATCTCGGCCA	AAAAGCAAGGCCTGCTTAATCAGATCTCGGCCATATGTGCGGCATTGACTAAGAG
CDH1--163	TATGTTGAACTCTTCCCGTATGCCATCTCCTGTAACCC	TATGTTGAACTCTTCCCGTATGCCATCTCCTGTAACCCTATGTGCGGCATTGACTAAGAG
CDH1--164	CAGGTGAAAGGGATGGATCATTCCTTCTCTGAAGGTCT	CAGGTGAAAGGGATGGATCATTCCTTCTCTGAAGGTCTTATGTGCGGCATTGACTAAGAG
CDH1--165	AAAACTATGCATTGGGGTGGAGAAATTTAGGGGCAGTC	AAAACTATGCATTGGGGTGGAGAAATTTAGGGGCAGTCTATGTGCGGCATTGACTAAGAG
CDH1--166	ATAGATGTTAGAACTGGACTGTGTCAGCTGGGTGTGG	ATAGATGTTAGAACTGGACTGTGTCAGCTGGGTGTGGTATGTGCGGCATTGACTAAGAG

CDH1--167	GCCAGGCCAGGCATTTTCTAGAGAAGGAATCCTG	GCCAGGCCAGGCATTTTCTAGAGAAGGAATCCTGTATGTGCGGCATTGACTAAGAG
CDH1--168	CACCCCTCAGCATGTTGCCCAAGAGGAATATTGC	CACCCCTCAGCATGTTGCCCAAGAGGAATATTGCTATGTGCGGCATTGACTAAGAG
CDH1--169	TTGGAATCTCACCTTCAGTACGAGGCTTCCTCCC	TTGGAATCTCACCTTCAGTACGAGGCTTCCTCCCTATGTGCGGCATTGACTAAGAG
CDH1--170	AAAAGGGAACAAGATGACCAACTTGTTGGTGGGGA	AAAAGGGAACAAGATGACCAACTTGTTGGTGGGGATATGTGCGGCATTGACTAAGAG
CDH1--171	CTGGGCACTTACTTTATGAACAGGTGGGGTGGG	CTGGGCACTTACTTTATGAACAGGTGGGGTGGGTATGTGCGGCATTGACTAAGAG
CDH1--172	ATAGTGACAGGTACTCTAGGTGCCGGCTCTTACTCA	ATAGTGACAGGTACTCTAGGTGCCGGCTCTTACTCATATGTGCGGCATTGACTAAGAG
CDH1--173	TAAGTCCCAGGAGATACTAGCCCTAGATCTCGTCTCTGT	TAAGTCCCAGGAGATACTAGCCCTAGATCTCGTCTCTGTTATGTGCGGCATTGACTAAGAG
CDH1--174	AGCTTCGTGCTAAAAGGTTTTGGAGAAATAACAGCTGCT	AGCTTCGTGCTAAAAGGTTTTGGAGAAATAACAGCTGCTTATGTGCGGCATTGACTAAGAG
CDH1--175	TGGTGACGTTCATGTGGCTGGTTTCATTACCACC	TGGTGACGTTCATGTGGCTGGTTTCATTACCACCTATGTGCGGCATTGACTAAGAG
CDH1--176	CTCACCTGTACAAAGGGGATAAACATAGCCCCTACCAC	CTCACCTGTACAAAGGGGATAAACATAGCCCCTACCACTATGTGCGGCATTGACTAAGAG
CDH1--177	AGTGCATGCTGATTGTGCCCGTGAATAGTTACTGC	AGTGCATGCTGATTGTGCCCGTGAATAGTTACTGCTATGTGCGGCATTGACTAAGAG
CDH1--178	ATCCTTCCTGTGCTCAGCGGCTCTATTTCTAAGAATGT	ATCCTTCCTGTGCTCAGCGGCTCTATTTCTAAGAATGTTATGTGCGGCATTGACTAAGAG
CDH1--179	TTCCATTCTAGATCCATCACTTTGGCCTAGAAAGGAGCC	TTCCATTCTAGATCCATCACTTTGGCCTAGAAAGGAGCCTATGTGCGGCATTGACTAAGAG
CDH1--180	GCTCTGTGTCAGTTACCAGCCTTGTTTCCAGGAG	GCTCTGTGTCAGTTACCAGCCTTGTTTCCAGGAGTATGTGCGGCATTGACTAAGAG
CDH1--181	TTTGGAGCTGACCCAGGGGTGATAGAAAACACTGG	TTTGGAGCTGACCCAGGGGTGATAGAAAACACTGGTATGTGCGGCATTGACTAAGAG
CDH1--182	TGTGAGAAAACTCCCTCGTTGGTTCTTCCTACAGGG	TGTGAGAAAACTCCCTCGTTGGTTCTTCCTACAGGGTATGTGCGGCATTGACTAAGAG
CDH1--183	AAGGCTAGCAAGCTGGGAGAACTTGGAGAACC	AAGGCTAGCAAGCTGGGAGAACTTGGAGAACCTATGTGCGGCATTGACTAAGAG
CDH1--184	GCCTGAGTGGTCTAGGCTGATACATCAGGCTTGG	GCCTGAGTGGTCTAGGCTGATACATCAGGCTTGGTATGTGCGGCATTGACTAAGAG

CDH1--185	CCCCACATGTTTATCCCAAATCCCCTTCTCCTCAAGC	CCCCACATGTTTATCCCAAATCCCCTTCTCCTCAAGCTATGTGCGGCATTGACTAAGAG
CDH1--186	TTGCTTCTAGGTGCCCATCTCACCCATCCTACC	TTGCTTCTAGGTGCCCATCTCACCCATCCTACCTATGTGCGGCATTGACTAAGAG
CDH1--187	AGGAAAGATGAATCGGAATGGAGCATTGCTGGGAAG	AGGAAAGATGAATCGGAATGGAGCATTGCTGGGAAGTATGTGCGGCATTGACTAAGAG
CDH1--188	TACTAGGGTGATCTGAGAAGGCCTCACTGAAGGGG	TACTAGGGTGATCTGAGAAGGCCTCACTGAAGGGGTATGTGCGGCATTGACTAAGAG
CDH1--189	CCATGTGACTAGGTAGGGAAGGGTGTTCTAGACGG	CCATGTGACTAGGTAGGGAAGGGTGTTCTAGACGGTATGTGCGGCATTGACTAAGAG
CDH1--190	GCAAAGACACACCCCTGACACTGAGCAGAAGG	GCAAAGACACACCCCTGACACTGAGCAGAAGGTATGTGCGGCATTGACTAAGAG
CDH1--191	GTACCCATCACCAGGACTTTGTCTTTCACTTGGGACT	GTACCCATCACCAGGACTTTGTCTTTCACTTGGGACTTATGTGCGGCATTGACTAAGAG
CDH1--192	GCCATCTTCGTTGTTGCTACCTCTAGGAAGCCTTCT	GCCATCTTCGTTGTTGCTACCTCTAGGAAGCCTTCTTATGTGCGGCATTGACTAAGAG
CDH1--193	GATACGTCCTACTCCTTCCCTTTGCCAGTTGGGA	GATACGTCCTACTCCTTCCCTTTGCCAGTTGGGATATGTGCGGCATTGACTAAGAG
CDH1--194	ACTCAGTGAGTAGTTTGATGAGGGGCTCTTTGCTGT	ACTCAGTGAGTAGTTTGATGAGGGGCTCTTTGCTGTTATGTGCGGCATTGACTAAGAG
CDH1--195	CCCCAGTGGAGGTGACTTATGTCCATCTTTTGGGG	CCCCAGTGGAGGTGACTTATGTCCATCTTTTGGGGTATGTGCGGCATTGACTAAGAG
CDH1--196	ACTTTTGCAGTGAGGGAGTGCATTATAATGGAGAGGAGG	ACTTTTGCAGTGAGGGAGTGCATTATAATGGAGAGGAGGTATGTGCGGCATTGACTAAGAG
CDH1--197	AAAAGCCCCAAACCAGCTGAGCTTAGATGGATCTC	AAAAGCCCCAAACCAGCTGAGCTTAGATGGATCTCTATGTGCGGCATTGACTAAGAG
CDH1--198	GTGCCAGAAGAAAATCCCCACTCCCTACAATGTGTG	GTGCCAGAAGAAAATCCCCACTCCCTACAATGTGTGTATGTGCGGCATTGACTAAGAG
CDH1--199	GGGGAAGAAGTCCAATTTCAGCTGGGAAAATGGGA	GGGGAAGAAGTCCAATTTCAGCTGGGAAAATGGGATATGTGCGGCATTGACTAAGAG
CDH1_546	/5Alex546N/CTCTTAGTCAATGCCGCACA	

References

1. Zhao, Y. *et al.* Nanoscale imaging of clinical specimens using pathology-optimized expansion microscopy. *Nat. Biotechnol.* **35**, 757–764 (2017).
2. Tillberg, P. W. *et al.* Protein-retention expansion microscopy of cells and tissues labeled using standard fluorescent proteins and antibodies. *Nat. Biotechnol.* **34**, 987–992 (2016).
3. Bucur, O. *et al.* Nanoscale imaging of clinical specimens using conventional and rapid-expansion pathology. *Nat. Protoc.* **15**, 1649–1672 (2020).
4. Ku, T. *et al.* Multiplexed and scalable super-resolution imaging of three-dimensional protein localization in size-adjustable tissues. *Nat Biotech* **34**, 973–981 (2016).
5. Susaki, E. A. *et al.* Whole-brain imaging with single-cell resolution using chemical cocktails and computational analysis. *Cell* **157**, 726–739 (2014).
6. Truckenbrodt, S. *et al.* X10 expansion microscopy enables 25-nm resolution on conventional microscopes. *EMBO Rep.* **19**, e45836 (2018).
7. Damstra, H. G. J. *et al.* Visualizing cellular and tissue ultrastructure using Ten-fold Robust Expansion Microscopy (TREx). *bioRxiv* 2021.02.03.428837 (2021). doi:10.1101/2021.02.03.428837
8. Ijdo, J. W., Wells, R. A., Baldini, A. & Reeders, S. T. Improved telomere detection using a telomere repeat probe (TTAGGG)n generated by PCR. *Nucleic Acids Res.* **19**, 4780 (1991).
9. Suntronpong, A. *et al.* CENP-B box, a nucleotide motif involved in centromere formation, occurs in a New World monkey. *Biol. Lett.* **12**, (2016).
10. Dertinger, T. *et al.* Fast, background-free, 3D super-resolution optical fluctuation imaging (SOFI). *Proc. Natl. Acad. Sci. U. S. A.* **106**, 22287–22292 (2009).
11. Dertinger, T., Xu, J., Naini, O. F., Vogel, R. & Weiss, S. SOFI-based 3D superresolution sectioning with a widefield microscope. *Opt. Nanoscopy* **1**, 1–5 (2012).
12. Moser, F. *et al.* Cryo-SOFI enabling low-dose super-resolution correlative light and electron cryo-microscopy. *Proc. Natl. Acad. Sci. U. S. A.* **116**, 4804–4809 (2019).
13. Wijesekara, P. *et al.* Engineering rotating apical-out airway organoid for assessing respiratory cilia motility. *iScience* **25**, (2022). doi: 10.1016/j.isci.2022.104730
14. Hua, K. & Ferland, R. J. Fixation methods can differentially affect ciliary protein immunolabeling. *Cilia* **6**, 5 (2017).
15. Halpern, A. R., Howard, M. D. & Vaughan, J. C. Point by Point: An Introductory Guide to Sample Preparation for Single-Molecule, Super-Resolution Fluorescence Microscopy. *Curr. Protoc. Chem. Biol.* **7**, 103–120 (2015).
16. Ponjavić, I., Vukušić, K. & Tolić, I. M. Expansion microscopy of the mitotic spindle. *Methods Cell Biol.* **161**, 247–274 (2021).
17. Whelan, D. R. & Bell, T. D. M. Image artifacts in single molecule localization microscopy: Why optimization of sample preparation protocols matters. *Sci. Rep.* **5**, 1–10 (2015).
