# Supplementary material for: Magnify is a universal molecular anchoring strategy for expansion microscopy
Source: Nat Biotechnol. 2023 Jan 2;41(6):858–69. doi: 10.1038/s41587-022-01546-1 (PMC10264239; doi:10.1038/s41587-022-01546-1)
Supplement: Supplementary file 2 [file 41587_2022_1546_MOESM2_ESM.pdf]

nature portfolio

Corresponding author(s): Yongxin Zhao

Last updated by author(s): Sep 26, 2022

Reporting Summary

Nature Portfolio wishes to improve the reproducibility of the work that we publish. This form provides structure for consistency and transparency in reporting. For further information on Nature Portfolio policies, see our Editorial Policies and the Editorial Policy Checklist.

Statistics

For all statistical analyses, confirm that the following items are present in the figure legend, table legend, main text, or Methods section.

n/a	Confirmed	
☐	☒	The exact sample size (*n*) for each experimental group/condition, given as a discrete number and unit of measurement
☐	☒	A statement on whether measurements were taken from distinct samples or whether the same sample was measured repeatedly
☐	☒	The statistical test(s) used AND whether they are one- or two-sided<br>*Only common tests should be described solely by name; describe more complex techniques in the Methods section.*
☐	☒	A description of all covariates tested
☒	☐	A description of any assumptions or corrections, such as tests of normality and adjustment for multiple comparisons
☐	☒	A full description of the statistical parameters including central tendency (e.g. means) or other basic estimates (e.g. regression coefficient) AND variation (e.g. standard deviation) or associated estimates of uncertainty (e.g. confidence intervals)
☐	☒	For null hypothesis testing, the test statistic (e.g. *F*, *t*, *r*) with confidence intervals, effect sizes, degrees of freedom and *P* value noted<br>*Give P values as exact values whenever suitable.*
☒	☐	For Bayesian analysis, information on the choice of priors and Markov chain Monte Carlo settings
☒	☐	For hierarchical and complex designs, identification of the appropriate level for tests and full reporting of outcomes
☒	☐	Estimates of effect sizes (e.g. Cohen's *d*, Pearson's *r*), indicating how they were calculated

Our web collection on statistics for biologists contains articles on many of the points above.

Software and code

Policy information about availability of computer code

Data collection	Imaging data was collected using the commercial Nikon NIS-Elements AR 5.21.03 64-bit software
Data analysis	FIJI ImageJ 1.53c AND https://github.com/zhao-biophotonics/MAGNIFY

For manuscripts utilizing custom algorithms or software that are central to the research but not yet described in published literature, software must be made available to editors and reviewers. We strongly encourage code deposition in a community repository (e.g. GitHub). See the Nature Portfolio guidelines for submitting code & software for further information.

Data

Policy information about availability of data

All manuscripts must include a data availability statement. This statement should provide the following information, where applicable:

- Accession codes, unique identifiers, or web links for publicly available datasets
- A description of any restrictions on data availability
- For clinical datasets or third party data, please ensure that the statement adheres to our policy

Sample data can be found at https://github.com/zhao-biophotonics/MAGNIFY. All other data can be provided upon reasonable request from the corresponding author.

Human research participants

Policy information about studies involving human research participants and Sex and Gender in Research.

Reporting on sex and gender	We have only used human tissue sections for technical demonstration. As biological conclusions were not drawn, the population characteristics are not relevant for this study. FFPE sex/gender information can be obtained from the supplier, USBiomax.
Population characteristics	We have only used human tissue for technical demonstration. As biological conclusions were not drawn, the population characteristics are not relevant for this study.
Recruitment	Human FFPE samples were commercially obtained from USBiomax and thus recruitment was not relevant for this study.
Ethics oversight	FFPE tissues were commercially obtained from USBiomax who collects human tissues under approved protocols.

Note that full information on the approval of the study protocol must also be provided in the manuscript.

Field-specific reporting

Please select the one below that is the best fit for your research. If you are not sure, read the appropriate sections before making your selection.

☒ Life sciences ☐ Behavioural & social sciences ☐ Ecological, evolutionary & environmental sciences

For a reference copy of the document with all sections, see nature.com/documents/nr-reporting-summary-flat.pdf

Life sciences study design

All studies must disclose on these points even when the disclosure is negative.

Sample size	No sample size calculation was performed. Experiments were repeated in triplicates at minimum, unless noted otherwise in the corresponding figure legend.
Data exclusions	No data was excluded.
Replication	All reported data has been reproduced over at least 3 technical and/or biological replicates. ROIs from different parts of tissue sections across multiple sections were used for quantification of biomolecule retention. Sequential tissue sections were used for quantification of FFPE tissue.
Randomization	Randomization was not necessary for this study.
Blinding	Blinding was not necessary for this study. In most cases, experimental conditions were discernible from obtained image data.

Reporting for specific materials, systems and methods

We require information from authors about some types of materials, experimental systems and methods used in many studies. Here, indicate whether each material, system or method listed is relevant to your study. If you are not sure if a list item applies to your research, read the appropriate section before selecting a response.

Materials & experimental systems

n/a	Involved in the study	
☐	☒	Antibodies
☐	☒	Eukaryotic cell lines
☒	☐	Palaeontology and archaeology
☐	☒	Animals and other organisms
☒	☐	Clinical data
☒	☐	Dual use research of concern

Methods

n/a	Involved in the study	
☒	☐	ChIP-seq
☒	☐	Flow cytometry
☒	☐	MRI-based neuroimaging

Antibodies

Antibodies used	See supplementary Tables 4-5<br>Primary Antibodies<br>Host Target Vendor Cat. Number

Chicken GFAP Abcam ab4674
Chicken GFP Abcam ab13970
Chicken NeuN Millipore ABN91
Chicken Tyrosine Hydroxalase Abcam ab76442
Chicken Vimentin Abcam ab24525
Goat PSD-95 Abcam ab12093
Goat CD4 R&D Systems AF-379-SP
Goat Talin-1 Novus AF5456-SP
Mouse Anti-Actin, α-Smooth Muscle Sigma Aldrich A5228
Mouse α-Tubulin Sigma Aldrich T6199
Mouse Bassoon Abcam ab82958
Mouse Cytokeratin Pan Type I/II Invitrogen MA5-13156
Mouse Synaptophysin Invitrogen MA1-213
Mouse CD11c Abcam ab11029
Mouse CD4 Origene UM870010
Mouse CD8 Invitrogen MA1-80231
Mouse CD8a (Alexa 488) Invitrogen 53-0008-82
Mouse VDACI Abcam ab14734
Rabbit α-Tubulin Proteintech 11224-1-AP
Rabbit ACTN4 Sigma Aldrich HPA001873
Rabbit Alpha-Internexin (INA) Sigma Aldrich HPA008057
Rabbit Amyloid Precursor Protein (APP) Sigma Aldrich HPA001462
Rabbit Anti-ATPase Inhibitory Factor 1 (ATPIF1) Millipore ABC137
Rabbit Anti-ATPase Inhibitory Factor 1 (ATPIF1) Proteintech 12067-1-AP
Rabbit GABRA1 Proteintech 12410-1-AP
Rabbit GABRB1 Proteintech 20183-1-AP
Rabbit GluR2 Proteintech 11994-1-AP
Rabbit Synaptophysin Proteintech 17785-1-AP
Rabbit CCR5 Proteintech 17476-1-AP
Rabbit CD45 Abcam ab10558
Rabbit IL2 Proteintech 26156-1-AP
Rabbit IL-6 Proteintech 21865-1-AP
Rabbit TCR alpha Antibody Novus NBP2-52684
Wheat Germ Agglutinin (WGA) CF555 Biotium 29076
Wheat Germ Agglutinin (WGA) CF640R Biotium 29026
NHS-ATTO-488 Sigma Aldrich 41698
NHS-ATTO-532 Sigma Aldrich 88793
Succinimidyl Ester (NHS) CF 555 Biotium 92130
Cy3 NHS ester (non-sulfonated) Glpbio Tech. GC12618-25
Vybrant™ DiD cell-labeling solution Invitrogen V-22887
Vybrant™ DiO cell-labeling solution Invitrogen V-22886
Vybrant™ DiI cell-labeling solution Invitrogen V-22885
Lycopersicon Esculentum (Tomato) Lectin (LEL) Vector Labs DL-1174-1

Secondary Antibodies
Reactivity Host Conjugate Vendor Catalog Number
Rabbit Goat DyLight550 Invitrogen SA5-10033
Rabbit Goat CF555 Biotium 20232
Rabbit Goat Alexa Fluor 488 Invitrogen A11034
Rabbit Goat CF640R Biotium 20202
Rabbit Goat Fab Fragment AF488 Jackson Immuno 111-547-003
Rabbit Donkey CF488A Biotium 20015
Rabbit Donkey Fab Fragment AF488 Jackson Immuno 711-547-003
Mouse Goat Alexa Fluor 488 Invitrogen A11001
Mouse Goat CF568 Biotium 20301
Mouse Donkey CF555 Biotium 20037
Mouse Donkey CF640R Biotium 20177
Mouse Donkey Fab Fragment AF488 Jackson Immuno 715-547-003
Chicken Goat Alexa Fluor 488 Invitrogen A11039
Chicken Goat DyLight 488 Invitrogen SA5-10070
Chicken Goat DyLight 550 Invitrogen SA5-10033
Chicken Goat CF488A Biotium 20020
Chicken Goat CF555 Biotium 20034
Chicken Goat CF640 Biotium 20084
Goat Donkey CF647 Biotium 20829
Streptavidin CF 640 Biotium 292037

Validation

All validation data is available on the manufacturers' website. No further validation was performed.

Eukaryotic cell lines

Policy information about cell lines and Sex and Gender in Research

Cell line source(s)

HEK-293FT cells were purchased from ThermoFisher Scientific, Bronchus-derived human airway basal stem cells (hABSCs)

Cell line source(s)	were purchased from Lonza. Additional hABSCs were obtained from surgical excess of de-identified tissues of healthy lung donors or donors carrying variants in CCDC39 gene (c.830_831delCA [p.Thr277Argfs*3) and c.1871_1872delTA(p.Ile624Lysfs*3), IRB ID# 201103213 with permission of the institutional review board at Washington University in Saint Louis. The U2OS cells were purchased from ATCC and a gift from the Lee lab at Carnegie Mellon University.
Authentication	Cell lines were not authenticated.
Mycoplasma contamination	Cells were not tested for Mycoplasma contamination.
Commonly misidentified lines (See ICLAC register)	No commonly misidentified lines were used.

Animals and other research organisms

Policy information about studies involving animals; ARRIVE guidelines recommended for reporting animal research, and Sex and Gender in Research

Laboratory animals	Male and female mice were used. C57BL/6 mice, SST-Cre mouse line: Jax 013044 crossed to Ai3 (Jax 007903), DAT-Cre mice with C57 background expressing AAV2/5-hSynapsin1-FLEX-axon-GCaMP6 and AAV5-Syn-FLEX-rc[ChrimsonR-tdTomato].
Wild animals	No wild animals were used.
Reporting on sex	Sex was not relevant to this study.
Field-collected samples	No field collected samples were used.
Ethics oversight	All experimental procedures involving animals were conducted in accordance with the NIH guidelines and were approved by the Institutional Animal Care and Use Committee at Carnegie Mellon University under the protocols AR202000020 (SST-Cre Mice) and PROTO201600011 (C57BL/6), and by Brown University Institutional Animal Care and Use Committee (DAT-Cre).

Note that full information on the approval of the study protocol must also be provided in the manuscript.
